# In-depth transcriptome profiling of Cherry Valley duck lungs exposed to chronic heat stress

**DOI:** 10.3389/fvets.2024.1417244

**Published:** 2024-07-22

**Authors:** Yi Liu, Dongyue Sun, Congcong Xu, Xiaoyong Liu, Min Tang, Shijia Ying

**Affiliations:** ^1^School of Life Sciences, Jiangsu University, Zhenjiang, Jiangsu, China; ^2^Institute of Animal Science, Jiangsu Academy of Agricultural Sciences, Nanjing, China; ^3^College of Animal Science and Technology, Nanjing Agricultural University, Nanjing, China; ^4^College of Animal Science and Technology, Beijing University of Agriculture, Beijing, China

**Keywords:** chronic heat stress, duck rearing, environmental temperature, high-throughput sequencing technology, ceRNA

## Abstract

Amidst rising global temperatures, chronic heat stress (CHS) is increasingly problematic for the poultry industry. While mammalian CHS responses are well-studied, avian-specific research is lacking. This study uses in-depth transcriptome sequencing to evaluate the pulmonary response of Cherry Valley ducks to CHS at ambient temperatures of 20°C and a heat-stressed 29°C. We detailed the CHS-induced gene expression changes, encompassing mRNAs, lncRNAs, and miRNAs. Through protein–protein interaction network analysis, we identified central genes involved in the heat stress response—*TLR7*, *IGF1*, *MAP3K1*, *CIITA*, *LCP2*, *PRKCB*, and *PLCB2*. Subsequent functional enrichment analysis of the differentially expressed genes and RNA targets revealed significant engagement in immune responses and regulatory processes. KEGG pathway analysis underscored crucial immune pathways, specifically those related to intestinal IgA production and Toll-like receptor signaling, as well as Salmonella infection and calcium signaling pathways. Importantly, we determined six miRNAs—miR-146, miR-217, miR-29a-3p, miR-10926, miR-146b-5p, and miR-17-1-3p—as potential key regulators within the ceRNA network. These findings enhance our comprehension of the physiological adaptation of ducks to CHS and may provide a foundation for developing strategies to improve duck production under thermal stress.

## 1 Introduction

The exponential growth of the global population necessitates substantial protein production, placing escalating demands on animal production systems, particularly within tropical and subtropical regions. This factor significantly contributes to ensuring global food security (1, 2). Among these production sectors, the poultry industry emerges as a crucial subsector that substantially fuels economic growth (3, 4). Nevertheless, the implications of global warming have engendered prolonged hyperthermia during the summer months, presenting a formidable challenge to the industry. This challenge has manifested in reduced productivity and substantial economic losses (5). This deleterious phenomenon, recognized as heat stress, has garnered extensive global attention and scrutiny (6).

Heat stress entails an imbalance between heat acquisition and dissipation, resulting in an elevation of poultry body temperature (7–9). It is typically categorized into intense or chronic heat stress (CHS), pertaining to brief or prolonged exposure to elevated ambient temperatures, respectively (10). Unfortunately, both intense and chronic heat stress can exact a toll on avian health (11, 12). This consequence extends to other domestic animals as well. Poultry, in particular, faces heightened vulnerability due to the absence of sweat glands and the majority of their body surface being covered in feathers. Consequently, the role of heat dissipation is largely assumed by the lungs in poultry (13, 14). When the ambient temperature falls within the thermal comfort zone, birds can sustain their body temperature with minimal effort. Typically, the optimal temperature for growing Pekin ducks ranges from 18 to 20°C (15). However, deviations from this range trigger behavioral, physiological, and metabolic adaptations aimed at temperature regulation and mitigating the impact of high temperatures (16–18). These adaptations include panting, reduced food intake, weight loss, and can culminate in undesirable outcomes such as increased feed conversion ratios (FCRs), stunted growth, and compromised meat quality. In cases where efficient heat dissipation is unattainable, multi-organ dysfunction may ensue, potentially leading to fatality (19–21).

Recent strides in high-throughput screening technology have culminated in the routine utilization of transcriptome sequencing for the quantification and identification of RNAs across diverse tissues and cells (22). RNA molecules bear transcribed genetic information that can be translated into proteins or directly/indirectly modulate gene expression levels (23). The variation in the number of different transcripts in response to temperature changes can offer valuable insights into cellular states and stress mechanisms (22, 24). Notably, heat shock proteins (HSPs) and phosphoinositide 3-kinase (PIK3) emerge as key participants in heat stress acclimation, with the genes encoding these proteins showing significant upregulation in heat-treated Fujian shelducks and Shan Ma, Pekin, Muscovy ducks (25–29). Furthermore, non-coding RNAs that play immunoregulatory roles during *Salmonella enteritidis* infection have been revealed through competing endogenous RNA (ceRNA) regulatory networks in Shaoxing ducks (30). Yet, the landscape of ceRNA networks under heat stress conditions remains largely uncharted.

In this pioneering study, we have created a CHS model in CVds—a breed with significant economic value—to explore the comprehensive transcriptomic alterations (31). Our innovative approach employs environmental control chambers for a precise induction of CHS, facilitating the in-depth examination of gene, miRNA, mRNA, and lncRNA expression variations using advanced sequencing techniques. Furthermore, we have delineated lncRNA-miRNA-mRNA networks to elucidate the intricate molecular dialogs during CHS, marking a novel foray into the full-spectrum transcriptomic impact of heat stress on these ducks.

## 2 Materials and methods

### 2.1 Animal resources, ethical approval and sample collection

This study was conducted in strict accordance with the regulations outlined by the Administration of Affairs Concerning Experimental Animals (Decree No. 63 of the Jiangsu Academy of Agricultural Science on 8 July 2014). All experimental procedures involving animals received ethical approval from the Research Committee of the Jiangsu Academy of Agricultural Sciences (Nanjing, China).

**Figure 1:**
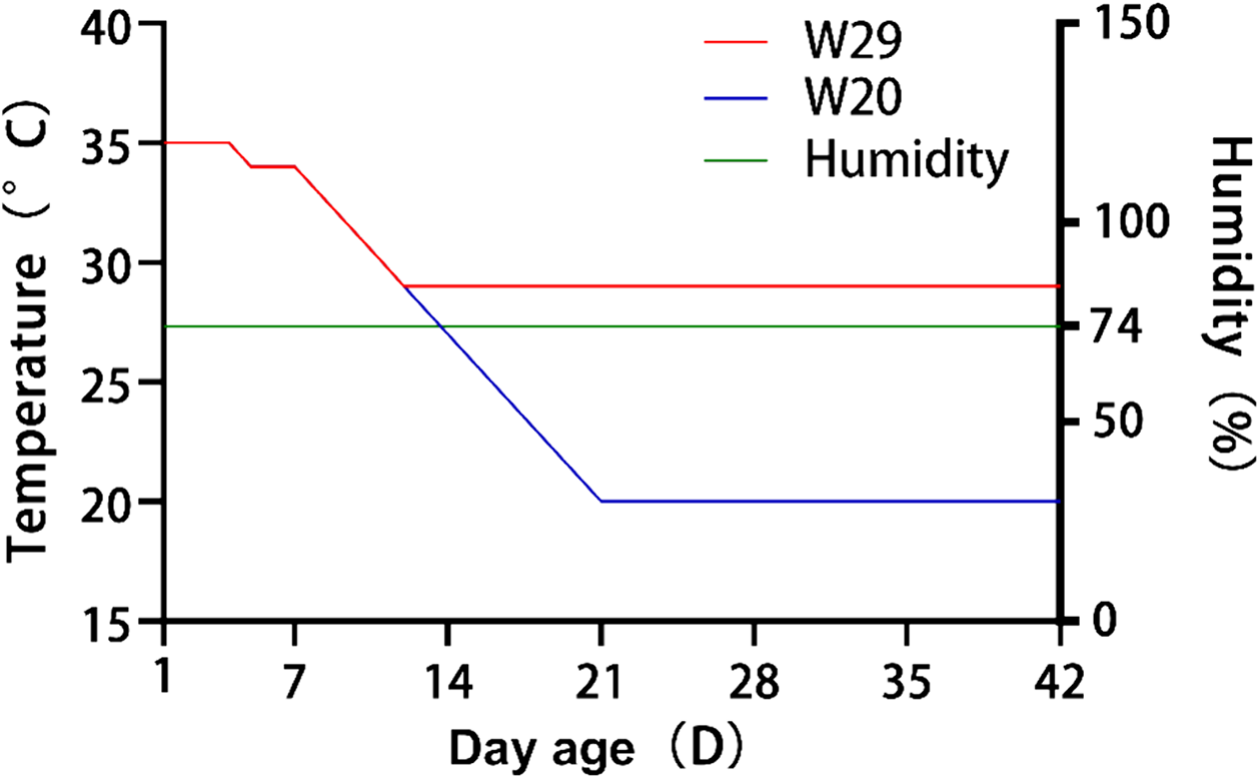
Detailed temperature regimens for W20 and W29 cohorts over the course of the investigation.

Following the approach detailed in (15, 32), a batch of 72 newly-hatched CVds with identical genetic backgrounds were procured from a commercial farm. These ducks were subsequently divided into two groups through random allocation, ensuring that there were no significant disparities in phenotypes or weights among any pair of groups. For brevity, these groups were subsequently referred to as W20 and W29. During the initial phase of rearing, all ducks were individually housed in separate pens equipped with an environmental control chamber, providing access to *ad libitum* feed and water (Supplementary Figure S1). Figure 1 illustrates that the ambient temperature was sustained at 35°C for the first 4 days and was subsequently decreased to 34°C for the following 3 days. Over days 8–12, the temperature in the W29 enclosure was gradually lowered by 1°C daily to 29°C, which was then sustained for 30 days. In parallel, the ambient temperature for W20 was similarly reduced by 1°C daily throughout this interval, leading to a steady state at 20°C (33). During the entire rearing phase, humidity was consistently maintained at 74% (34), with all ducks having free access to standard commercial pellet feed and water from a drip-nipple system.

Ultimately, at the market-age of day 43, three samples were collected from each group were humanely stunned with a 36 V electrified pool and then euthanized via jugular vein dissection (35). As the second key organ for heat dissipation, their lungs were carefully excised, promptly frozen in liquid nitrogen and then stored at −80°C prior to dispatch to the sequencing company.

### 2.2 Microscopic observation of duck lungs

The lungs of each selected duck from both groups were examined under a microscope at a magnification of 20×. Lung tissue samples from CVds were carefully extracted and subsequently fixed in 4% paraformaldehyde for a duration of 24 h to preserve the cellular structure for detailed histological analysis. Subsequently, the samples underwent dehydration using a graded alcohol series. These specimens were then infiltrated and embedded in paraffin, before being sectioned into thin serial slices of approximately 5 μm thickness. These sections were mounted onto glass slides, subjected to hematoxylin and eosin staining, and observed using an Olympus microscope (BX53).

### 2.3 RNA extraction, library preparation and full transcriptome sequencing

For each group, total RNA was isolated from three biological replicates of lung tissue using TRIzol^®^ reagent (Invitrogen, United States), following the manufacturer’s protocol, and genomic DNA was removed with DNase I RNase-free (TaKara, Japan). The RNA concentration and integrity were verified using a NanoDrop 2,100 spectrophotometer (Thermo Fisher Scientific, United States) and an Agilent Bioanalyzer 2,100 (Agilent Technologies, United States), respectively. Only high-quality RNA samples (OD260/280 = 1.8 ~ 2.2, OD260/230 ≥ 2.0, RIN ≥ 6.5, 28S:18S ≥ 1.0, >1 μg) were used for sequencing library construction.

Libraries for mRNA and lncRNA were prepared using a ribosomal RNA depletion strategy with the NEBNext^®^ Ultra™ Directional RNA Library Prep Kit for Illumina (New England Biolabs, United States), starting with 3 μg of RNA per sample. miRNA libraries were generated using the QIAseq miRNA Library Kit (Qiagen, Germany), adhering to the supplier’s instructions. Based on the length distribution characteristic of miRNA, target fragments (16–35 nt) were isolated by gel excision on a 6% Novex TBE PAGE gel (1.0 mm, 10 well) (Invitrogen, United States). Quantification was performed using the Qubit 4.0 fluorometer (Thermo Fisher Scientific, United States), and sequencing was carried out on an Illumina NovaSeq 6,000 system (Illumina, United States) by Shanghai Majorbio Bio-pharm Biotechnology Co., Ltd. (Shanghai, China).

### 2.4 Data preprocessing and quality control

After the above deep sequencing, the raw paired-end reads were preprocessed using SeqPrep^1^ and Sickle^2^ by removing adaptor sequences and filtering low-quality reads (Supplementary Table S1). Clean reads were aligned using HISAT to the high-resolution reference genome of *Anas platyrhynchos* (*A. platyrhynchos* GCF_015476345.1, https://www.ncbi.nlm.nih.gov/genome/2793?genome_assembly_id=1498951) (36, 37). The mapped reads were assembled by StringTie in a reference-based approach (38). Also, the number of reads mapped to each transcript was calculated using RSEM, and the transcripts per million reads (TPM) was estimated to measure the expression level of each gene/transcript (39).

### 2.5 Bioinformatics analysis at gene expression level and mRNA expression level

Gene expression level is a broader term that encompasses the levels of all products of gene expression, including mRNA, non-coding RNAs, and proteins. mRNA expression level is a subset of this, focusing only on the messenger RNA produced during transcription. The DESeq2 package within R software was employed to identify genes and mRNAs that were differentially expressed between W20 and W29, with an emphasis on those exhibiting a greater than 1.5-fold change and an adjusted padj below 0.05, marking them as statistically significant (40). These findings were further examined for functional relevance using GO and KEGG pathway enrichment via GOATOOLS and KOBAS (41, 42). Additionally, differentially expressed genes (DEGs) were integrated into STRING (version 12.0) and Cytoscape (version 11.0.13) to delineate protein–protein interaction (PPI) networks, highlighting key modules using the cytoHubba plugin (43–45). Enrichment analysis of interactive gene targets was conducted using the BINGO plugin and the clusterProfiler package (46, 47).

### 2.6 Identification of lncRNA and prediction of target RNAs

Using the StringTie tool (38) with its default settings, we reassembled transcripts from the aligned clean reads. Subsequently, we cross-referenced these merged transcripts against known reference transcripts annotated in GFF/GTF formats and existing lncRNA databases to identify recognized lncRNAs. In addition, we pinpointed putative novel lncRNA transcripts by their length, requiring over 200 base pairs, and by the presence of two or more exons. To assess the coding potential of these transcripts, we employed a suite of tools: CNCI (48) with a score threshold below zero, CPC (49) with a score under 0.5, CPAT (50) with a score below 0.5, and Pfam with an e-value stricter than 1e-3 but not passing the threshold. Transcripts that were consistently predicted to lack protein-coding potential by these metrics were classified as novel lncRNAs. Potential *cis*- and *trans*-acting target mRNAs of lncRNAs were identified through an examination of gene expression patterns and chromosomal positioning, as outlined by (51). For *cis*-acting targets, genes situated within a 100,000 base-pair range flanking the lncRNA were pinpointed utilizing BEDTOOLS software as described by (52). The analysis of lncRNA-mediated trans-regulation was predicated on the correlation coefficient between the expressions of lncRNA and mRNA, with coefficients exceeding 0.9 signifying a potential trans-regulatory interaction.

### 2.7 Identification of miRNA and prediction of target RNAs

Initially, all clean mapped tags were matched to known miRNAs using the miRBase (version 22.1) database^3^. Subsequently, the remaining tags were cross-referenced with the Rfam and Repbase databases to filter out ribosomal RNA (rRNA), transfer RNA (tRNA), small nuclear RNA (snRNA), small nucleolar RNA (snoRNA), other non-coding RNAs, and repeats. Finally, unannotated tags were evaluated for potential novel miRNAs with miRdeep2 software (53), based on their genomic location and the formation of hairpin structures. The miRanda algorithm (54) was applied to animal samples, while psRobot (55) was utilized for insect samples to forecast miRNA targets. Predicted miRNA-target RNAs were then determined by identifying the overlap in the outcomes from both tools.

### 2.8 Differentially expressed RNAs analysis

The refined datasets for lncRNAs, miRNAs, and mRNAs were obtained by discarding low-quality reads from the initial raw data, ensuring a Phred quality score of at least 20. RNAs that showed no expression in over three samples were excluded from subsequent analyses to maintain data integrity. Consequently, only high-quality filtered datasets were utilized for further analysis. To quantify RNA expression, the polished reads from the lncRNA and mRNA libraries were mapped to the reference genome via the STAR aligner, while the miRNA sequences were aligned to miRBase using the BOWTIE tool. The R software’s limma package (56) was utilized for identifying differentially expressed mRNAs, lncRNAs, and miRNAs (DEmRNAs, DElncRNAs, and DEmiRNAs). Significant DEmRNAs and DElncRNAs were detected within the comparison groups, applying a threshold of padj <0.05 and an absolute log2FC greater than 1. For DEmiRNAs, the criteria of an absolute Log2FC greater than 0.585 and a *p*-adjust value below 0.05 were adopted. Functional enrichment analyses for GO terms and KEGG pathways were conducted on the target genes of DEmiRNAs and DElncRNAs, as well as on DEmRNAs, using the GOATOOLS and KOBAS tools (42).

### 2.9 Construction of the lncRNA-miRNA-mRNA regulatory network

To elucidate the relationships between DEmRNAs, DElncRNAs, and DEmiRNAs, a lncRNA-miRNA-mRNA regulatory network was established rooted in the ceRNA hypothesis. Predictions for miRNA-lncRNA and miRNA-mRNA pairings were conducted utilizing Miranda and Targetscan (57), respectively, while the Spearman correlation coefficient, hinged on expression levels, was employed to assess the interplay among these pairings. Visualization of the intricate network was achieved through Cytoscape software.

### 2.10 RT-qPCR validation for the expression level of DEmRNAs, DEmiRNAs, and DElncRNAs

The cDNA synthesis for mRNA involved reverse transcription using HiScript III RT SuperMix with gDNA wiper (Vazyme, China) in a thermal cycler, following the protocol provided by the manufacturer. The inverse transcription reaction (ITR) for mRNA was executed in a 20 μL reaction mix, incubated at 37°C for 15 min and 85°C for 5 s, then cooled to 4°C. For lncRNA, cDNA synthesis was performed using the lnRcute lncRNA cDNA First-Strand Synthesis Kit and FastKing One Step First-Strand Synthesis Kit (both from Tiangen, Beijing, China). The cDNA for miRNA was synthesized using the miRNA First Strand cDNA Synthesis (Stem-loop Method) Kit (Sangon Biotech, China), with the ITR for both lncRNA and miRNA conducted as per their respective kits’ guidelines.

Primer sets for mRNA were crafted using Primer Premier 6 software, while primer sets for lncRNA and miRNA were designed via Sangon Biotech’s online tools^4^. All primers used in this study were synthesized by Genewiz^@^ (China), based on sequences in GenBank and the reference genome. Then, quantitative real-time PCR (RT-qPCR) was carried out using SYBR Green Master Mix (Yeasen, Shanghai, China) on an ABI 7500 Sequence Detector (Applied Biosystem, United States) according to the manufacturer’s instructions. The reaction mixture included 10 μL of first-strand cDNA, 0.4 μM each of forward and reverse primers, and 10 μL of 2× SYBR Green Master Mix, totalling 20 μL. For miRNA, the MicroRNAs qPCR Kit (SYBR Green Method) from Sangon Biotech was employed. The RT-qPCR protocol consisted of an initial denaturation at 95°C for 2 min, followed by 34 cycles of 95°C for 10 s and 60°C for 30 s. All assays were performed in triplicate, with GAPDH as the internal control gene for mRNA and lncRNA, and U6 snRNA as the internal control for miRNA expression studies (37, 58). Melting curve analysis confirmed the specificity of the amplification, and relative gene expression was quantified using the 2−^ΔΔCt^ method.

## 3 Results

### 3.1 Growth performance and carcass traits

Supplementary Figure S2 reveals that at the 43-day collection point, W20 exhibited superior performance over W29 in the majority of carcass characteristics. For a more comprehensive analysis, please consult our previously published work (1). These findings suggest that the growth rate of CVds decelerates when ambient temperatures rise from 20°C to 29°C, aligning with the findings reported in (4).

### 3.2 Histopathological examination

**Figure 2:**
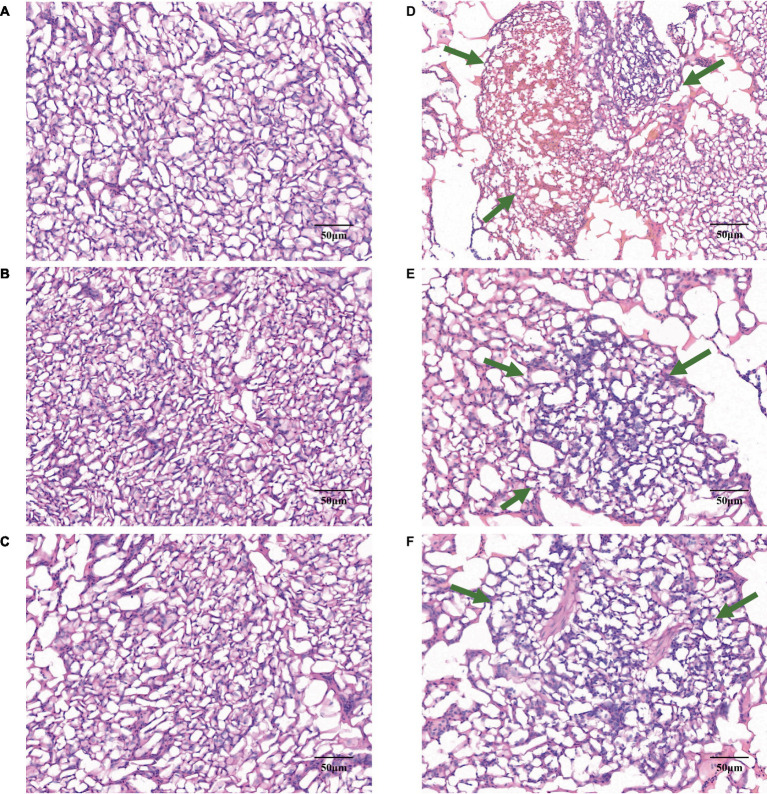
Gene expression profiles in CVds at 43 days of age. The cross-sectional areas of CVds lung tissue reared in an environmental control chamber at **(A–C)** 20°C and **(D–F)** 29°C. In panels **(D–F)**, the green arrows point to areas of inflammatory tissue.

In the comparative illustration provided by Figure 2, the lungs of W20 exhibited a robust, reddish color and no exudate, with the alveoli retaining their proper form and exhibiting only minor signs of inflammation. The lung interstitium was also free from any signs of excess fluid. In stark contrast, the W29 samples showed significant exudation and altered alveoli that no longer maintained their usual shape. These samples demonstrated considerable changes including thickened alveolar walls, capillary closure, and the presence of inflammatory cells within the alveolar passages, along with interstitial swelling. Yet, these issues were less pronounced than in previous W29 samples.

### 3.3 Gene expression divergence and subsequent analysis

**Figure 3:**
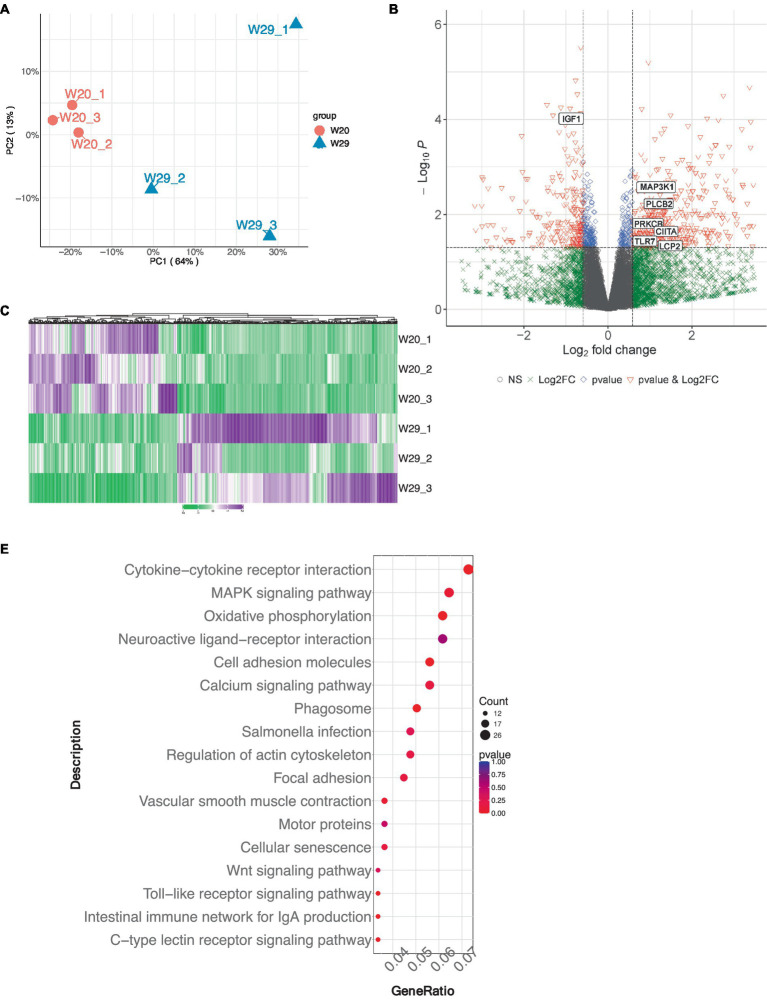
Gene expression profile analysis in CVds at 43 days of age. **(A)** Principal Component analysis plot for six samples. **(B)** Volcano plot visualizing the gene expression profile, created using the ComplexHeatmap package in R. **(C)** Heatmap representation of selected differentially expressed genes. **(D)** Gene Ontology enrichment analysis of DEGs. **(E)** Kyoto Encyclopedia of Genes and Genomes pathway enrichment analysis for DEGs.

To elucidate the genetic underpinnings of heat tolerance in CVds, transcriptomic analyses were conducted on individual lung samples from both W20 and W29 groups using the Illumina NovaSeq 6,000 system. Each of the three cDNA libraries yielded a substantial number of clean reads, amassing billions of nucleotides in total. More than 91% of the clean reads from each library can be mapped to the reference genome of *A. platyrhynchos*. The expression level of each transcript was measured using TPM method. For the evaluation of gene expression variance, the profiles of the longest transcript isoforms were considered. Followed Principal Component Analysis (PCA) demonstrated a marked distinction between the W20 and W29 sample groups (Figure 3A).

**Figure 4:**
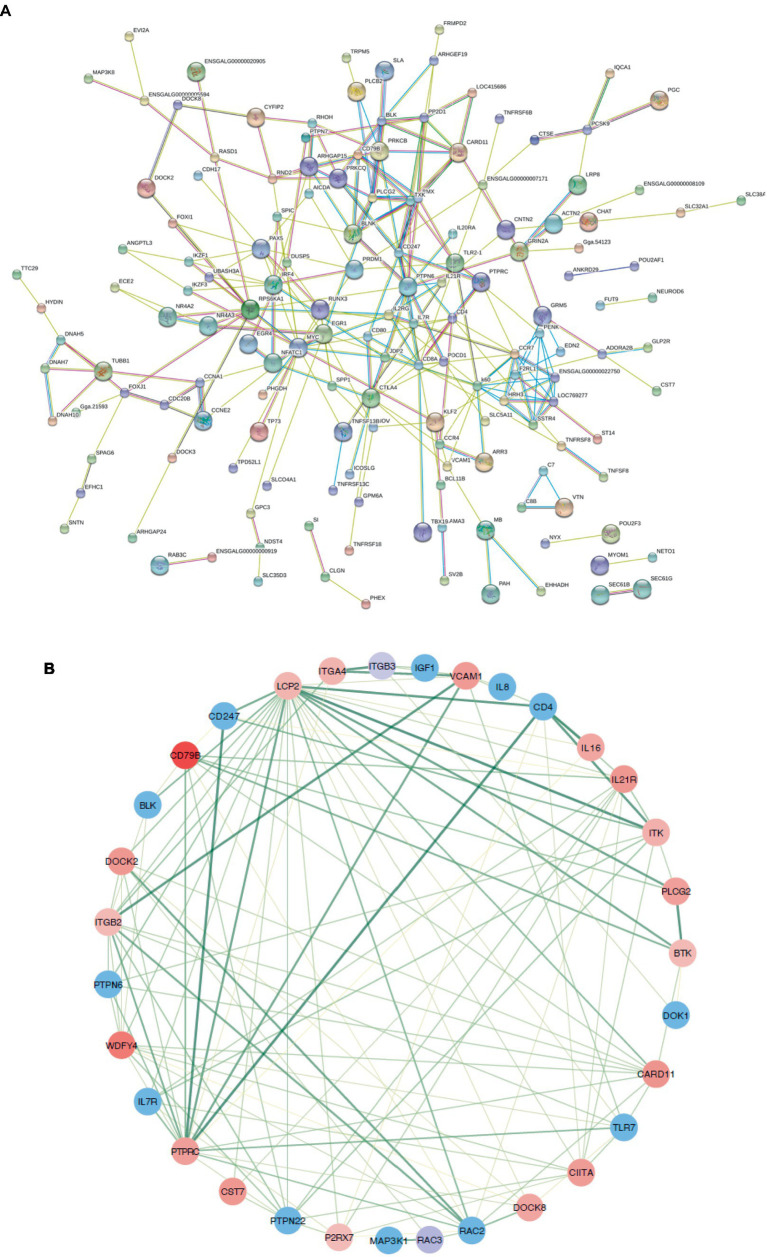
Protein–protein interaction networks. **(A)** A comprehensive PPI network for all differentially expressed genes, purged of any isolated nodes; **(B)** a focused subnetwork displayed in a circular layout to emphasize connectivity.

Genes exhibiting an absolute fold change of at least 1.5, coupled with a padj under 0.05, were classified as DEGs (Supplementary Table S2). From the W20/W29 comparison, a total of 1,013 DEGs were discovered, among which were 605 up-regulated and 408 down-regulated, as shown in Figures 3B,C. Based on the MCC algorithm and literature research, the top genes were identified as potential hub genes, which were *TLR7*, *IGF1*, *MAP3K1*, *CIITA*, *LCP2*, *PRKCB*, and *PLCB2* (Figure 4).

Subsequently, the GOATOOLS software was employed to pinpoint GO terms significantly enriched within these DEGs. A Fisher’s exact test, adjusted for multiple testing with the Benjamin-Hochberg method, flagged GO terms with a false discovery rate less than 0.05 as significantly enriched. Functional annotation of GO terms revealed DEGs predominantly engaged in four biological processes: ‘immune response,’ ‘immune system process,’ ‘regulation of immune system process,’ and ‘response to external stimulus,’ mirroring findings from multiple other studies. In terms of cellular components, the majority of associated GO terms pertained to membrane-related structures, including ‘membrane,’ ‘mitochondrial inner membrane,’ and ‘organelle membrane’ (Figure 3D). These DEGs were predominantly found to be connected to immune system pathways, including ‘intestinal immune network for IgA production,’ ‘Toll-like receptor signaling pathway,’ and ‘C-type lectin receptor signaling pathway.’ Notably, a significant number of these DEGs were also enriched in pathways related to ‘oxidative phosphorylation’ and were implicated in ‘Salmonella infection’ (Figure 3E).

### 3.4 Characterization and analysis of differentially expressed mRNA

**Figure 5:**
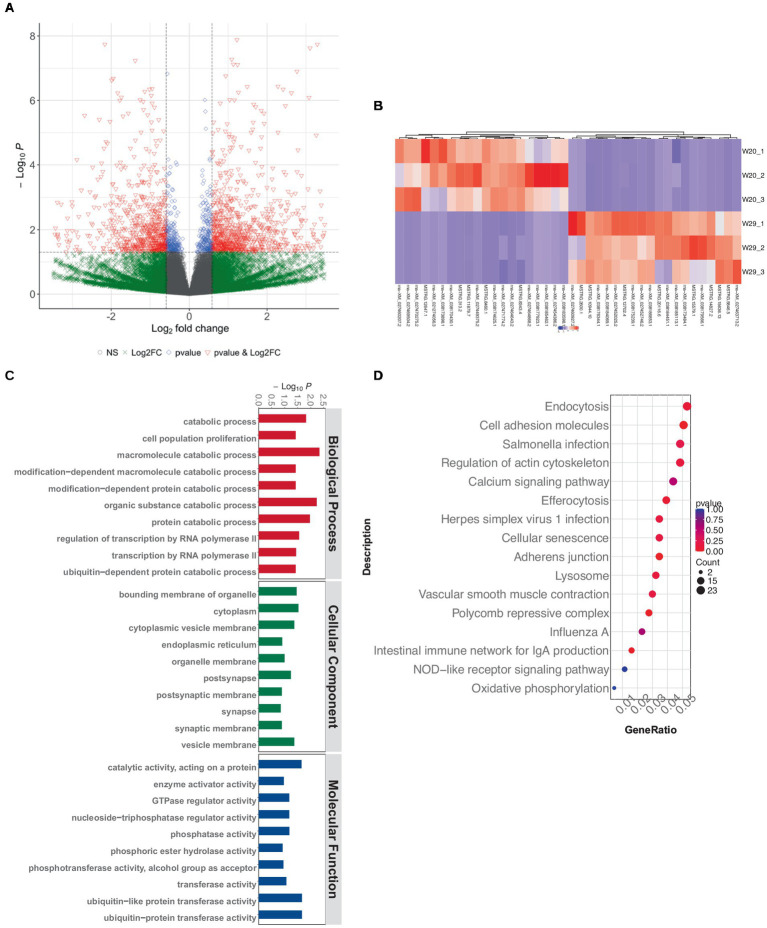
Differentially expressed mRNA profiling in CVds at 43 days. **(A)** Volcano plot visualization of mRNA expression profiles generated with the ComplexHeatmap package in RStudio. **(B)** Heatmap depiction of top DEmRNAs. **(C)** GO enrichment analysis of DE-mRNAs. **(D)** KEGG pathway enrichment for DEmRNAs.

Utilizing quality-assured RNAseq data, we delineated 34,363 protein-encoding mRNAs via custom shell scripts developed in-house. Of which, there were 1,434 mRNA observed to be significantly differentially expressed, with 803 up-regulated and 631 down-regulated (Figures 5A,B).

The GO term functional annotation highlighted that DEmRNAs were actively involved in breakdown processes, including the catabolism of macromolecules and organic substances, as well as protein metabolism. Cellular component analysis showed an abundance of GO terms related to ubiquitin-related enzyme activities, specifically ‘ubiquitin-protein transferase activity’ and ‘ubiquitin-like protein transferase activity’ (Figure 5C). Additionally, these DEmRNAs showed a significant presence in immune system pathways, like ‘Toll-like receptor signaling’ and ‘C-type lectin receptor signaling.’ Notably, a greater number of terms were associated with infectious diseases, such as those related to ‘Herpes simplex virus 1 infection,’ ‘Influenza A’ and ‘Salmonella infection’ (Figure 5D).

### 3.5 Characterization and analysis of differentially expressed lncRNA and functional enrichment analysis of predicted targets

**Figure 6:**
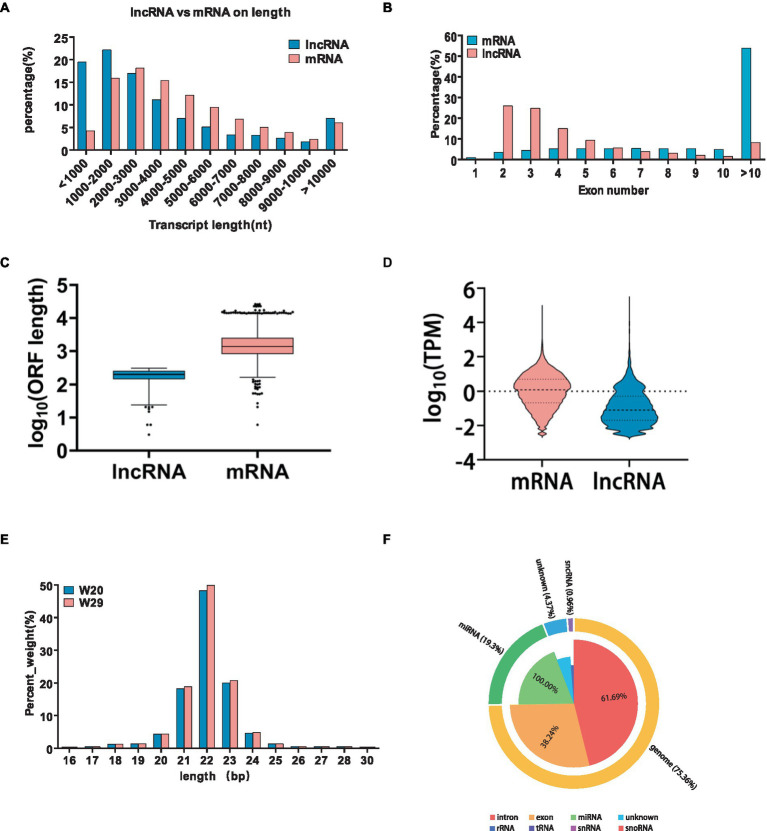
Characterization and comparative analysis of differentially expressed lncRNAs under CHS. **(A–D)** Comparative metrics between lncRNAs and mRNAs, including transcript length, exon count, open reading frame size, and expression levels measured as transcripts per million. **(E)** Categorization of the identified small RNAs. **(F)** Distribution of transcript lengths for all identified miRNAs in the two groups.

In the realm of gene regulation, lncRNAs act as *cis*-regulators, often influencing proximate protein-coding genes. Utilizing refined RNAseq data subjected to quality control, our tailored shell scripts facilitated the identification of 5,352 (4,387 known and 965 novel) lncRNA transcripts. The genomic analysis revealed that while lncRNAs and mRNAs share similar transcript lengths, lncRNAs are more likely to have longer sequences exceeding 3,000 bp. LncRNAs typically feature a greater proportion with 2–5 exons and possess shorter open reading frames (ORFs) and lower expression levels as quantified by FPKM (Figures 6A–D).

**Figure 7:**
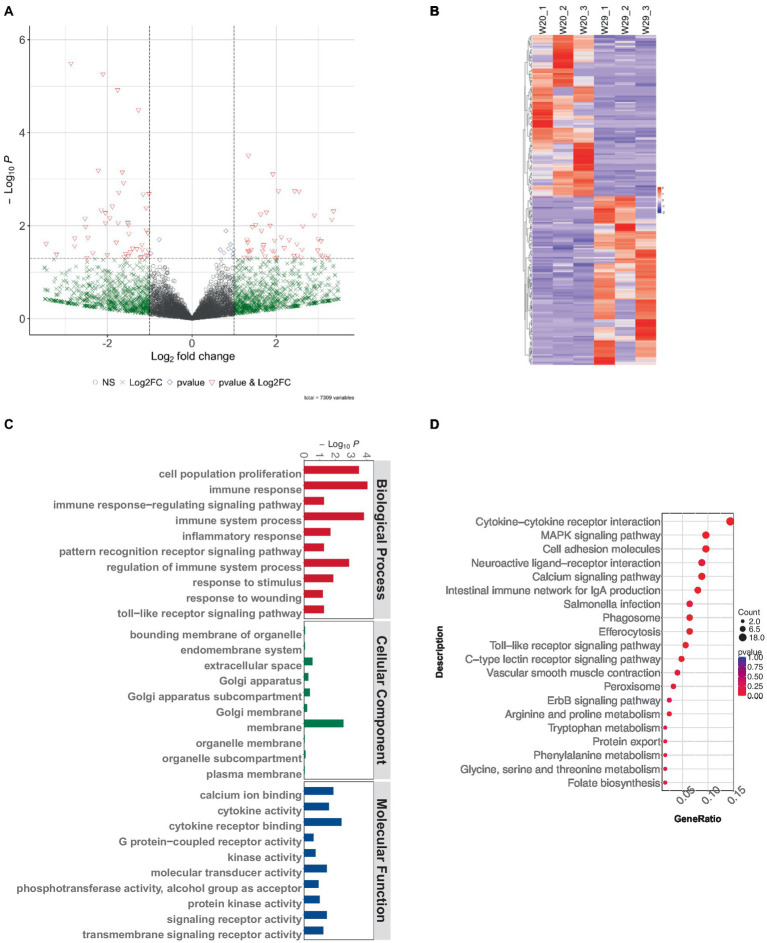
Exploring lncRNA variances in CVds at 43 days. **(A)** Visualization of lncRNA expression variations using a volcano plot from the ComplexHeatmap package. **(B)** A heatmap delineating notable DElncRNAs. **(C)** Analysis of GO term enrichment among DElncRNA targets. **(D)** Examination of DElncRNA targets within KEGG pathway frameworks.

Subsequent analysis revealed 217 differentially expressed lncRNAs within the W29 profile, comprising 111 that were up-regulated and the rest displaying down-regulation (Figures 7A,B). Plus, GO and KEGG pathway enrichment analyses were undertaken to decipher the functions and pathways associated with the predicted targets. The enriched GO terms were principally connected to immunoreaction, such as ‘immune response,’ ‘immune system process,’ and ‘regulation of immune system process’ (Figure 7C). The KEGG pathway analysis revealed involvement in immune system (e.g., Intestinal immune network for IgA production) and Infectious disease (e.g., *Salmonella* infection) (Figure 7D).

### 3.6 Characterization and analysis of differentially expressed miRNA and functional enrichment analysis of predicted targets

In this study, we generated a substantial number of raw reads, totaling 12,706,326, 10,121,409, and 9,982,953 for W20, and 12,311,631, 12,412,586, and 10,192,859 for W29, respectively. Following the removal of adaptor sequences, low-quality sequences, and reads outside the length range of 18 to 32 nucleotides, we obtained high-quality clean reads: 12,553,092, 10,032,226, and 9,858,879 for W20, and 11,971,885, 12,250,135, and 9,864,788 for W29. The majority of these clean reads ranged in length from 20 to 24 nucleotides (Figure 6E). Upon classifying the small RNAs, we discovered that 75.3% of the clean reads were attributed to intronic (61.69%) and exonic (38.24%) regions. Additionally, miRNAs accounted for 19.3% of the reads, small non-coding RNAs (sncRNAs) comprised 0.96, and 4.37% were categorized as other types (Figure 6F).

**Figure 8:**
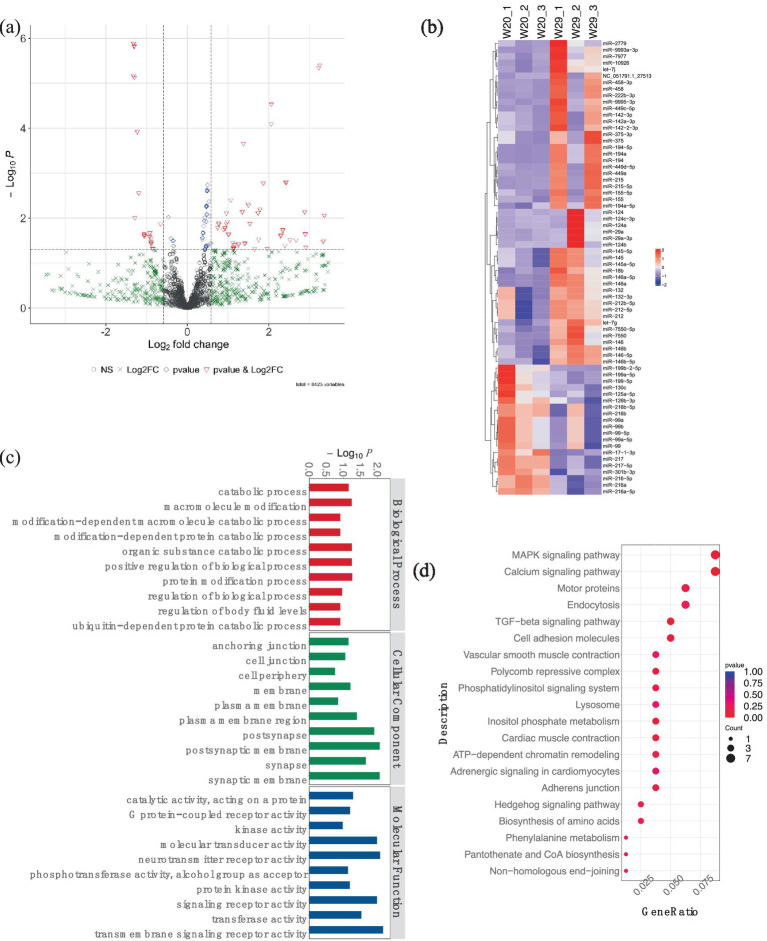
Analysis of differentially expressed miRNAs in CVds at 43 days. **(A)** A volcano plot generated with the ComplexHeatmap package in RStudio to visualize miRNA expression profiles. **(B)** Heatmap representation highlighting top DEmiRNAs. **(C)** GO enrichment analysis for predicted targets of DEmiRNAs. **(D)** Assessment of DEmiRNAs within the context of KEGG pathways.

Additionally, for the miRNA expression profiles, 543 DEmiRNAs were identified in W29, including 413 up-regulated and 130 down-regulated miRNAs (Figures 8A,B). In the target study of W29-specific DEmiRNAs, GO annotation revealed a preponderance linked to molecular functions like receptor activities such as ‘neurotransmitter receptor activity,’ ‘signaling receptor activity,’ and ‘transmembrane signaling receptor activity.’ Cellular component annotations centered around structures such as membranes and organelles, while molecular functions primarily involved binding and catalysis. The enriched GO terms for known DEmiRNAs were associated with catabolic processes (Figure 8C). In KEGG pathway analysis, the targets of DEmiRNAs were involved in MAPK signaling pathway, Calcium signaling pathway, and TGF-beta signaling pathway (Figure 8D).

### 3.7 CeRNA regulatory network in response to CHS

**Figure 9:**
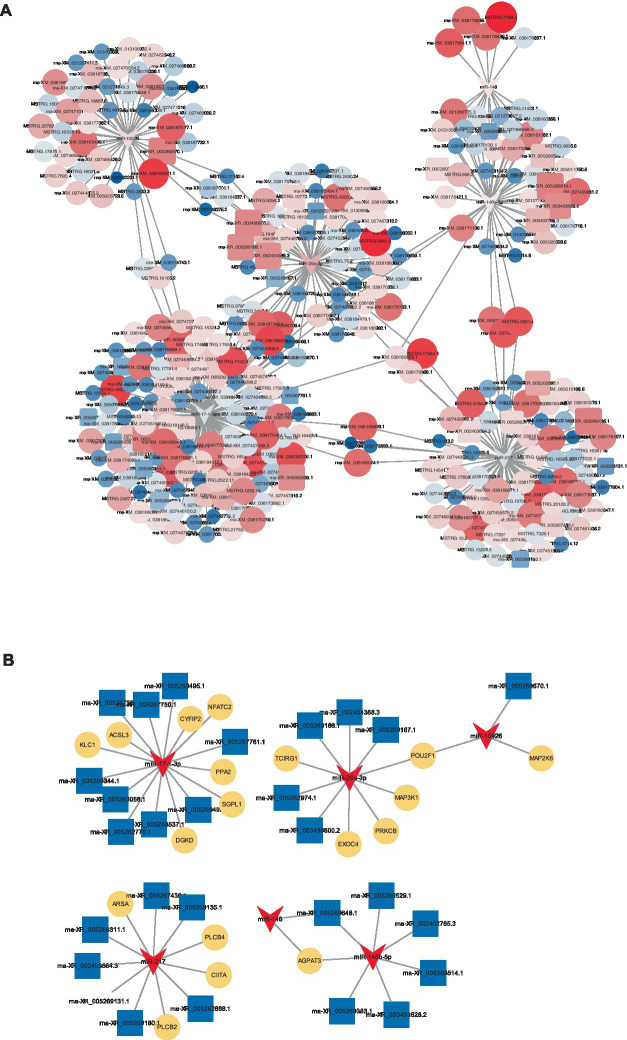
CeRNA network. **(A)** A constructed ceRNA network centered around miR-217, miR-146b-5p, miR-29a-3p, miR-10926, miR-17-1-3p, and miR-146. In this network, dark blue arrows represent miRNAs, pink triangles indicate mRNAs, light blue circles denote lncRNAs. **(B)** Depiction of mini-ceRNA network involving six DE-miRNAs and their associated target genes.

To elucidate the comprehensive regulatory matrix involving protein-coding RNAs and non-coding RNAs in response to CHS, a complex ceRNA network was established, integrating differentially expressed miRNAs, mRNAs and lncRNAs. This expansive network predicted thousands of mRNAs and dozens of lncRNAs as miRNA targets in the lungs of W29. Filtering interactions by a strong negative correlation revealed several thousand potential miRNA-mRNA and a handful of miRNA-lncRNA linkages. Certain miRNAs (miR-146, miR-217, miR-29a-3p, miR-10926, miR-146b-5p, and miR-17-1-3p) emerged as central hubs, potentially key to regulatory mechanisms, while multiple lncRNAs (LOC101804558, LOC113841824, LOC101798355, LOC119717605, and LOC110353088) were also pinpointed as significant network participants (Figure 9).

### 3.8 RT-qPCR confirmation of miRNA-ceRNA correlation in CHS responses

**Figure 10:**
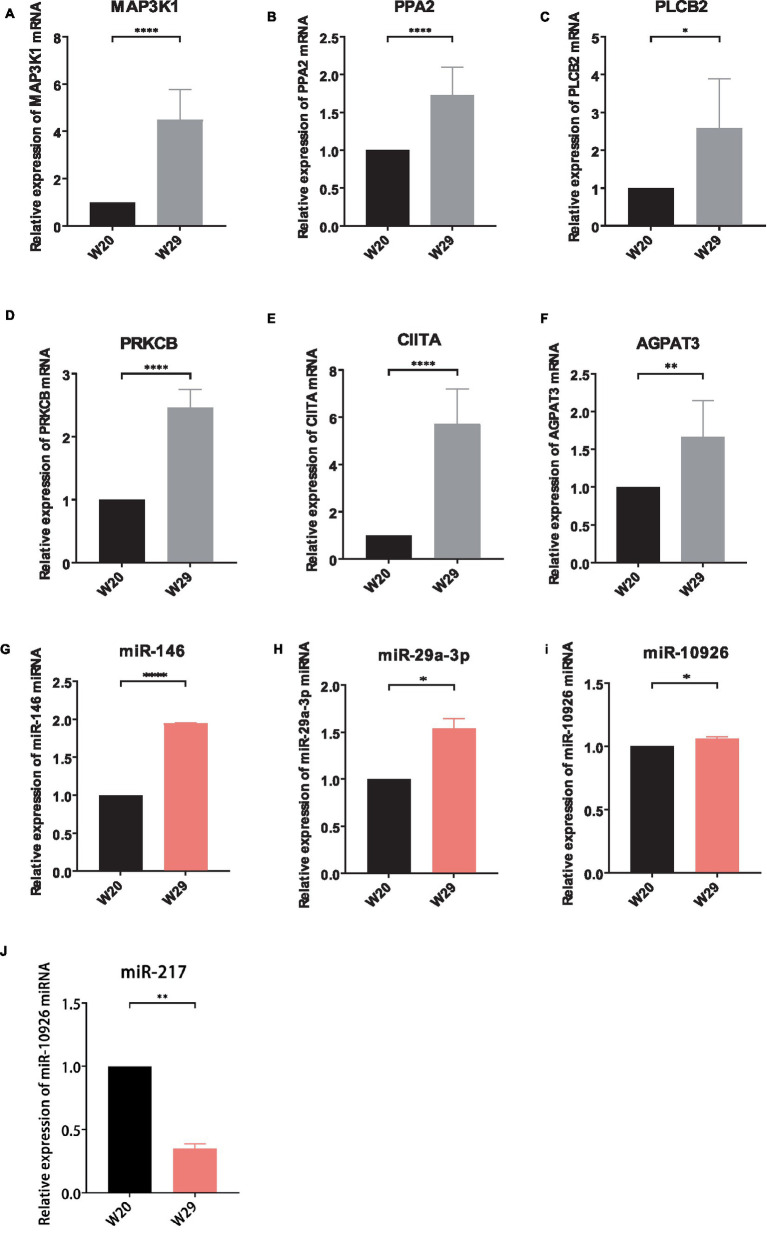

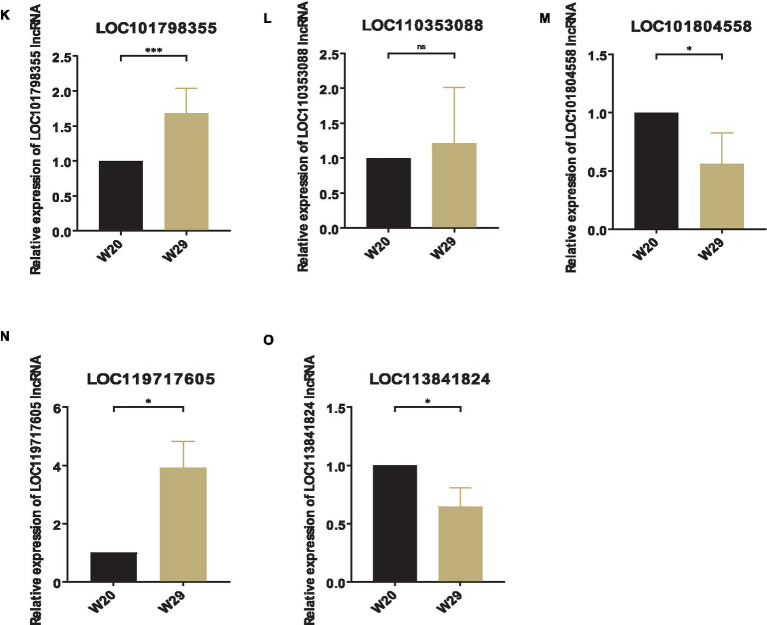
Validation of different expressed RNAs via RT-qPCR. **(A–F)** mRNA, **(G–J)** miRNA, **(K–O)** lncRNA. The relative expressions were calculated in triplicate using the method of 2^−△△^Ct and presented as mean ± SD. *p-value <0.05, **p-value <0.01, ***p-value <0.001, ****p-value <0.0001.

To substantiate the RNAseq data and examine the expression correlation between miRNAs and their targets, we selected four key miRNAs—miR-146, miR-217, miR-29a-3p, and miR-10926—and their associated mRNAs and lncRNAs from the ceRNA network for RT-qPCR analysis. The primer sequences for the mRNAs and lncRNAs are itemized in Tables 1, 2, respectively, incorporating GAPDH as the internal reference gene. The primers for the miRNAs are cataloged in Table 3, with U6 employed as the internal normalization gene. The analysis affirmed a predominantly accurate reflection of regulatory dynamics, with miRNAs and their respective targets displaying the expected regulatory trends of either up- or down-regulation (Figure 10). Notably, miR-146 was observed to be up-regulated, with its predicted targets being up-regulated, while miR-217 demonstrated down-regulation, accompanied by up-regulation of all its targets. These results not only validate the RNAseq data’s accuracy but also the postulated inverse relationship between the expression levels of miRNAs and their corresponding ceRNAs.

**Table 1:** Primer sequences for mRNA quantification via RT-qPCR.

mRNA name	GenBank accession No.	Primer sequences (5′–3′)	Product size (bp)
AGPAT3	rna-XM_038172299.1	F: CACAGTTCTCCTCTCGCCTCTC	170
		R: ATTCTTGGTTGCCGTAGCTGGA	
PPA2	MSTRG.7142.3	F: GCCACTGAGGAGCCGTTGAATC	119
		R: GTCTGAGGGAGGGCACCGTAAT	
CIITA	rna-XM_027469345.2	F: AGCAGGAGAAGCAAGTGGAAGA	266
		R: CTGGTGAGTTAGCGAGGTGGAG	
PLCB2	rna-XM_005019168.5	F: GCGATGTGGCTGAAGAGGAACC	295
		R: CGGCTCATCTGTCGCTTGTTGT	
MAP3K1	rna-XM_038170332.1	F: TGCCAACAGTCGAACGAGTCAA	188
		R: CCAGTAGTGCTTGCCAGTTGCT	
PRKCB	rna-XM_038186960.1	F: CCTGACTACATCGCACCTGAGA	202
		R: AGATCGCCACTGCCTCCTTG	
GAPDH	rna-XM_03818058.4	F: GGTTGTCTCCTGCGACTTCA	165
		R: TCCTTGGATGCCATGTGGAC	

F, forward primer sequence; R, reverse primer sequence. GAPDH, internal control gene.

**Table 2:** Primer sequences for miRNA quantification via RT-qPCR.

miRNA name	Target name	GenBank accession No.	Primer sequences (5′–3′)
miR-146	AGPAT3	rna-XM_005012470.5	F: GCGCTGAGAACTGAATTCCA
	AGPAT3	rna-XM_038172299.1	R: GTGCAGGGTCCGAGGT
miR-29a-3p	EXOC4	MSTRG.75.2	F: GCGCTAGCACCATCTGAAAT
	MAP3K1	rna-XM_038170337.1	
	MAP3K1	rna-XM_038170332.1	
	POU2F1	rna-XM_027449375.2	
	POU2F1	rna-XM_038184037.1	R: GTGCAGGGTCCGAGGT
	PRKCB	rna-XM_038186960.1	
	TCIRG1	rna-XM_038179683.1	
miR-10926	MAP2K6	rna-XM_038165339.1	F: GCGCGCATCCCAGCGGTG
	POU2F1	rna-XM_038184037.1	R: GTGCAGGGTCCGAGGT
	POU2F1	rna-XM_027449375.2	
miR-146	LOC101805192	rna-XR_005269648.1	F: GCGCTGAGAACTGAATTCCA
			R: GTGCAGGGTCCGAGGT
miR-29a-3p	LOC119718278	rna-XR_005269166.1	F: GCGCTAGCACCATCTGAAAT
	LOC119715672	rna-XR_005262974.1	
	LOC101791220	rna-XR_003496600.2	R: GTGCAGGGTCCGAGGT
	LOC119718278	rna-XR_005269167.1	
	LOC106018689	rna-XR_002404388.3	
miR-10926	LOC110351219	rna-XR_005260670.1	F: GCGCGCATCCCAGCGGTG
			R: GTGCAGGGTCCGAGGT
U6			F: CTCGCTTCGGCAGCACA
			R: AACGCTTCACGAATTTGCGT

F, forward primer sequence; R, reverse primer sequence. U6, internal control gene.

**Table 3:** Primer sequences for lncRNA quantification via RT-qPCR.

lncRNA name	GenBank accession No.	Primer sequences (5′–3′)	Product size (bp)
LOC101804558	rna-XR_005269495.1	F: CTGCCTCACCTCTTCGTCTTGG	96
		R: GCTGCTTCTGTCCTTCTCACTCC	
LOC113841824	rna-XR_005267325.1	F: GCAAAGCACAGCCAGCAGTTAC	107
		R: ACAGATACCGCATCCAGAGAAGAAG	
LOC101798355	rna-XR_005260529.1	F: GCAGAGGGTGAGGTGGTTGTC	114
		R: TTCTGGAGGAGCCTTGCATAAGC	
LOC119717605	rna-XR_005267439.1	F: GGAGCAGGACACAGCCACTAAC	127
		R: GCACAGGACAGACGGACAGAC	
LOC110353088	rna-XR_005266311.1	F: CCTGCTGCTGCTGCTCCTG	130
		R: GGGTGGCATCTTCCTCCTTCTTC	
GAPDH	rna- XM_03818058.4	F: GGTTGTCTCCTGCGACTTCA	165
		R: TCCTTGGATGCCATGTGGAC	

F, forward primer sequence; R, reverse primer sequence. GAPDH, internal control gene.

## 4 Discussion

The detrimental physiological effects of CHS on poultry and livestock industries are well-documented. These effects encompass reductions in feed intake, feed efficiency, growth performance, meat and egg production, meat quality, and survival rates (15, 24, 32, 59–67). Moreover, various studies have explored the impact of CHS on inflammatory responses, dysbiosis, reactive oxidative stress (ROS), signal reactions, and energy metabolism (68–76). Some studies have specifically investigated the health status and well-being of ducks under increasing ambient temperatures, focusing on granulosa cells and the jejunum (25, 77). As the one of the main high-throughput sequencing technologies, transcriptomics has been facilitating the poultry rearing in recent years (78). In the current study, we made full transcriptome profiling of lungs detached from two groups of CVds at 43 days of age reared under 20 and 29°C ambient temperatures. In order to understand the molecular mechanism of response to CHS, comprehensive bioinformatics analysis and intensive RT-qPCR experiments was implemented.

Initial findings indicate that CHS significantly modulates a range of physiological aspects in CVds through pivotal genes including *TLR7*, *IGF1*, *MAP3K1*, *CIITA*, *LCP2*, *PRKCB*, and *PLCB2*. Notably, *TLR7*, highly expressed in duck lung tissue, is a key gene in the innate immune defense against viral infections such as influenza (78–80), and also plays a role in egg production (79–83). Immunostimulants are reported to enhance antioxidant and immune efficacy by stimulating *TLR7* expression (84, 85). *CIITA* (Class II major histocompatibility complex trans-activator) is integral to the innate immune response, functioning as a trans-activator that boosts MHC-II expression in both antigen-presenting and virus-infected cells. This activation sparks antiviral responses in the host, serving as a blockade against viral replication and aiding in the clearance of viral infections (86, 87). *IGF1* is acknowledged as a key gene influencing growth, body composition, and the development of metabolic and skeletal traits, and plays a significant role in the growth of various tissues, including muscle and bone (88–95). Moreover, its correlation with reproductive efficiency underscores its importance in developmental biology and poultry breeding programs (96–100). Publications concerning *MAPK3K1* (mitogen-activated protein kinase kinase kinase 1) (101, 102), *LCP2* (103) are limited, yet these genes are known for their roles in lipid metabolism and the development of fatty liver disease. *PRKCB* (protein kinase C alpha) (104), and *PLCB2* (Phospholipase C Beta 2) are less frequently mentioned. The comprehensive analysis incorporating differential expression and RT-qPCR suggests the potential of these genes as biomarkers for heat stress in CVds.

Similarly, it was found that biological profiles, inflammation, and stress protein markers were significantly enriched in miRNA target genes in differential genomes, including L1RAPL2, IL7R, TRAF3, TRAF5, HSPA8, etc. IL1RAPL2 is a molecule in the IL1R family that has different biological effects on immune and inflammatory responses. There is evidence to suggest that IL1RAPL2 is a specific biomarker for kidney injury (105). IL7R is often regulated to varying degrees after virus attacks on poultry. Similarly, there is evidence to suggest that IL7R is associated with cellular responses to heat exposure (106). TRAF3 is a key innate immune regulatory factor that plays a crucial role in defending against viral invasion (107, 108). TRAF5 has been found to regulate inflammation and apoptosis of atherosclerosis, steatosis and melanoma cells, and also plays an important role in regulating myocardial I/R injury (109). HSPA8 can significantly affect the proliferation, apoptosis, and immune function of poultry macrophages, while significantly promoting the proliferation of HD11 cells and inhibiting their apoptosis, with pro-inflammatory effects (110).

Subsequent analysis of gene enrichment pointed to CHS significantly bolstering immunological processes, with a particular focus on catabolic pathways in the case of DEmRNAs and targets of DEmiRNAs. Across the differentially expressed RNAs, pathways in the immune system, especially those involved in IgA production and Toll-like receptor signaling, were highlighted. These enrichments align with the literature on CHS’s impact on animal health (1, 12, 26, 70, 75, 76). The pathway of *Salmonella* infection consistently appears as one of the most enriched, reflecting the pathogen’s notoriety as a leading poultry-associated foodborne illness. This observation aligns with findings that heat stress may compromise the immune defenses, potentially increasing the risk of *Salmonella* infection in poultry, as noted in recent studies (31). The calcium signaling pathway, akin to the *Salmonella* infection pathway, has shown deep involvement in response to heat stress (Figures 3E, 5D, 7D). Elevated temperatures can increase membrane fluidity and permeability, leading to a calcium imbalance and the release of cytochrome c into the cytoplasm, triggering apoptosis through factors like caspases (63). Simultaneously, ROS generated under heat stress can inflict oxidative damage on enzymes responsible for muscle calcium regulation, further disturbing cellular homeostasis. As a result, high temperatures lead to reduced levels of calcium and phosphorus in the plasma of laying hens. These minerals are crucial for egg production and the quality of the eggshell (18, 104, 111).

In addition, this study presents, for the first time, a batch of miRNAs which play a role in CHS response of duck. In the foundational stages of inflammation, miR-146 is upregulated following exposure to lipopolysaccharide LPS, a bacterial element detected by *TLR4*. This triggers a series of events where NFκB migrates to the nucleus, initiating the expression of genes responsible for inflammatory mediators (112–114). Research has strengthened miR-146’s role in this pathway, underscoring its function in a negative feedback mechanism that tempers the inflammatory response initiated by *TLR4* (115). MiR-146b-5p is typically upregulated in response to *Salmonella enterica* infection, playing a pivotal role in maintaining immune balance by dampening the initiation of the innate immune reaction (116, 117). This miRNA has been shown to facilitate replication of the Duck Tembusu virus through the suppression of the *RPS14* gene, demonstrating its negative regulatory capacity in immune processes (118). miR-217 is noted for its regulatory influence on diverse muscle cell types by modulating critical genes, targeting *ROCK1* in vascular smooth muscle cells and *FGFR2* in skeletal muscle progenitors (119, 120). In the context of chicken liver cancer cells, miR-29a-3p responds to selenium levels and can instigate cell movement and invasion. It does this by focusing on the *COL4A2* gene, leading to the suppression of the *RhoA/ROCK* signaling pathway (121). To date, the roles of miR-10926 and miR-17-1-3p remain unexplored, signaling the need for further investigation into their functions.

However, it’s important to acknowledge the limitations of this study. Firstly, we focused solely on morphological changes in lung tissue, and future research should incorporate biochemical tests of lung tissue and morphological tests of skin tissue, as skin is the largest heat dissipation system. Secondly, while the short-read transcriptome sequencing and data analysis provided valuable insights, they do not provide a complete view. The list of DEGs obtained here includes some unreported genes that likely play crucial roles in the CHS-induced response of ducks. These genes should be characterized in future studies. Furthermore, investigating the mechanisms that regulate the expression of these genes is essential, as is the use of other omics approaches (multi-omics studies). Additionally, the present study could benefit from additional examinations, such as assessing amino acid composition, nutritional value, and protein digestibility (122).

As a brief supplement, several strategies have been proposed to mitigate the adverse effects of CHS, such as improving housing, ventilation, and cooling systems (e.g., using little rearing systems and cage rearing systems) (123, 124), dietary supplementation (e.g., with vitamin A, vitamin C, vitamin E, Glutamine, and Herbs) (125–129), feed additives (e.g., probiotics, prebiotics, polyphenols, and palm oils) (130, 131), and other approaches (e.g., feed restrictions and genetic selection for heat tolerance) (132–136).

## 5 Conclusion

In essence, this study aims to delve into the effects of varying ambient temperatures on the growth performance of CVds and shed light on the underlying mechanisms responding to heat stress using cutting-edge high-throughput sequencing technologies. In conclusion, like other poultry, CVds are also sensitive to high ambient temperatures, and environmental control chambers offer advantages in improving their quality of life (20, 137, 138). Considering the predictions of continued climate change in most models (19, 139), further research is urgently needed to uncover the response mechanisms and regulatory networks of ducks to CHS.

## Data availability statement

The original contributions presented in the study are included in the article/Supplementary material, further inquiries can be directed to the corresponding authors.

## Ethics statement

The animal study was approved by Administration of Affairs Concerning Experimental Animals (Decree No. 63 of the Jiangsu Academy of Agricultural Science on 8 July 2014). The study was conducted in accordance with the local legislation and institutional requirements.

## Author contributions

YL: Data curation, Formal analysis, Writing – original draft. DS: Resources, Writing – review & editing. CX: Resources, Visualization, Writing – review & editing. XL: Project administration, Resources, Writing – review & editing. MT: Supervision, Writing – review & editing. SY: Funding acquisition, Project administration, Resources, Writing – review & editing.

## Footnotes

^1^ https://github.com/jstjohn/SeqPrep

^2^ https://github.com/najoshi/sickle

^3^ http://www.mirbase.org/

^4^ https://store.sangon.com/newPrimerDesign
