# Supplementary material for: In-depth transcriptome profiling of Cherry Valley duck lungs exposed to chronic heat stress
Source: Front Vet Sci. 2024 Jul 22;11:1417244. doi: 10.3389/fvets.2024.1417244 (PMC11298465; doi:10.3389/fvets.2024.1417244)
Supplement: Supplementary file 1 [file Table_1.docx]

| **Samples** | **Total clean reads** | **Total clean bases** | **unique match** | **multi-position match** | **Total mapped reads** | **Total unmapped reads** |
| --- | --- | --- | --- | --- | --- | --- |
| W20_1 | 140085820 (100.00%) | 17575757205 (100.00%) | 104694517 (74.47%) | 24316000 (17.36%) | 129010517 (92.09%) | 11075303 (7.91%) |
| W20_2 | 152129548 (100.00%) | 19161362732 (100.00%) | 116442590 (76.54%) | 24961089 (16.41%) | 141403679 (92.95%) | 10725869 (7.05%) |
| W20_3 | 141677462 (100.00%) | 18577850816 (100.00%) | 113895963 (80.39%) | 16964272 (11.97%) | 130860235 (92.36%) | 10817227 (7.64%) |
| W29_1 | 132793402 (100.00%) | 17260232547 (100.00%) | 104917382 (79.01%) | 17256557 (13.00%) | 122173939 (92.00%) | 10619463 (8.00%) |
| W29_2 | 140933246 (100.00%) | 17798316784 (100.00%) | 112350813 (79.72%) | 18298590 (12.98%) | 130649403 (92.70%) | 10283843 (7.30%) |
| W29_3 | 118826918 (100.00%) | 15501757897 (100.00%) | 94655519 (79.66%) | 15227136 (12.81%) | 109882655 (92.47%) | 8944263 (7.53%) |

**Table S1 | Total Count of Filtered Reads Aligned to the *A. platyrhynchos* Reference Genome (GCF_015476345.1)**
