# Supplementary material for: In-depth transcriptome profiling of Cherry Valley duck lungs exposed to chronic heat stress
Source: Front Vet Sci. 2024 Jul 22;11:1417244. doi: 10.3389/fvets.2024.1417244 (PMC11298465; doi:10.3389/fvets.2024.1417244)
Supplement: Supplementary file 2 [file Table_2.docx]

**Table S2 | List of Differentially Expressed mRNAs in Duck Lung Tissue**

| **mRNA_name** | **baseMean** | **log2FoldChange** | **lfcSE** |  | **stat** | **pvalue** | **padj** | **gene_name** |
| --- | --- | --- | --- | --- | --- | --- | --- | --- |
| MSTRG.10.2 | 1087.625 | 1.310618 | 0.480916 |  | 2.725252 | 0.006425 | 0.229626 | ARSA |
| MSTRG.10048.1 | 42.77965 | -1.45542 | 0.590302 |  | -2.46556 | 0.01368 | 0.35748 | LZTS2 |
| MSTRG.10144.34 | 1175.911 | 1.21831 | 0.412589 |  | 2.952838 | 0.003149 | 0.146593 | ABLIM1 |
| MSTRG.10176.3 | 157.8477 | 10.78241 | 2.7294 |  | 3.95047 | 7.80E-05 | 0.009435 | RGS10 |
| MSTRG.10309.16 | 25.65399 | 2.279992 | 0.916691 |  | 2.487198 | 0.012875 | 0.343146 | GTDC1 |
| MSTRG.10320.5 | 477.3055 | 2.161859 | 0.629274 |  | 3.435479 | 0.000592 | 0.045671 | ARHGAP15 |
| MSTRG.10387.30 | 23.10107 | 8.010133 | 3.309845 |  | 2.420093 | 0.015517 | 0.377942 | NCKAP5 |
| MSTRG.10430.19 | 881.2857 | -1.13082 | 0.171847 |  | -6.58036 | 4.69E-11 | 3.14E-08 | CLASP1 |
| MSTRG.10450.8 | 30.36436 | -8.36135 | 3.221718 |  | -2.59531 | 0.009451 | 0.289182 | MYLK |
| MSTRG.10484.2 | 36.98783 | -1.27515 | 0.579301 |  | -2.20118 | 0.027723 | 0.512467 | SPEG |
| MSTRG.10525.14 | 38.19382 | 1.400577 | 0.705474 |  | 1.985301 | 0.047111 | 0.636734 | COBLL1 |
| MSTRG.10654.5 | 169.6837 | -7.41021 | 2.46989 |  | -3.00022 | 0.002698 | 0.130913 | ABI2 |
| MSTRG.10658.1 | 178.6212 | 1.251685 | 0.555851 |  | 2.251834 | 0.024333 | 0.479837 | LOC101803044 |
| MSTRG.10658.2 | 124.1526 | 1.108615 | 0.547195 |  | 2.025995 | 0.042765 | 0.612974 | LOC101803044 |
| MSTRG.10777.1 | 446.9295 | -1.73432 | 0.840194 |  | -2.06419 | 0.039 | 0.591784 | UBE2F |
| MSTRG.10861.7 | 209.9961 | -11.1513 | 2.161147 |  | -5.15991 | 2.47E-07 | 7.30E-05 | MYO1B |
| MSTRG.10944.10 | 350.1744 | 8.029582 | 1.136319 |  | 7.066307 | 1.59E-12 | 1.53E-09 | TGFBR3 |
| MSTRG.10944.9 | 728.1818 | -1.02627 | 0.171741 |  | -5.97567 | 2.29E-09 | 1.25E-06 | TGFBR3 |
| MSTRG.10984.4 | 212.076 | 1.595026 | 0.70569 |  | 2.260236 | 0.023807 | 0.474174 | F3 |
| MSTRG.10984.6 | 229.2329 | 1.590099 | 0.594182 |  | 2.676115 | 0.007448 | 0.251114 | F3 |
| MSTRG.11071.5 | 320.6065 | 4.163511 | 1.931826 |  | 2.155221 | 0.031145 | 0.539815 | PTPRC |
| MSTRG.11071.8 | 124.1098 | 4.214718 | 1.912109 |  | 2.204225 | 0.027508 | 0.511105 | PTPRC |
| MSTRG.11071.9 | 1070.191 | 1.881054 | 0.637144 |  | 2.95232 | 0.003154 | 0.146657 | PTPRC |
| MSTRG.11120.6 | 257.3324 | 1.489487 | 0.296404 |  | 5.025194 | 5.03E-07 | 0.000136 | PACS2 |
| MSTRG.11132.2 | 83.5229 | 1.88483 | 0.895066 |  | 2.1058 | 0.035222 | 0.563863 | PRDX6 |
| MSTRG.11299.4 | 110.2091 | 1.265486 | 0.475453 |  | 2.661644 | 0.007776 | 0.256185 | ZNF326 |
| MSTRG.11423.1 | 1302.434 | -1.9202 | 0.733296 |  | -2.61859 | 0.008829 | 0.277258 | PTPRF |
| MSTRG.11424.3 | 55.12861 | -9.22184 | 3.008372 |  | -3.06539 | 0.002174 | 0.112516 | KDM4A |
| MSTRG.11435.2 | 807.5855 | -1.73864 | 0.354178 |  | -4.90895 | 9.16E-07 | 0.000221 | KLF17 |
| MSTRG.11500.5 | 40.56006 | 8.822001 | 3.122996 |  | 2.824852 | 0.00473 | 0.187239 | MOB3C |
| MSTRG.11526.4 | 521.5783 | 1.100853 | 0.389613 |  | 2.825505 | 0.004721 | 0.187055 | OSBPL9 |
| MSTRG.11612.1 | 31.85941 | -8.43069 | 3.400896 |  | -2.47896 | 0.013177 | 0.348541 | ROR1 |
| MSTRG.11683.4 | 16.12843 | -7.44865 | 3.54974 |  | -2.09837 | 0.035873 | 0.569189 | COMMD2 |
| MSTRG.11879.7 | 415.0847 | -8.17396 | 1.119067 |  | -7.30426 | 2.79E-13 | 3.49E-10 | EIF4G1 |
| MSTRG.11900.7 | 531.2404 | 1.088414 | 0.339875 |  | 3.202398 | 0.001363 | 0.084411 | ABCC5 |
| MSTRG.11920.4 | 296.3383 | -6.60495 | 1.46968 |  | -4.49414 | 6.99E-06 | 0.001254 | NCL |
| MSTRG.12002.5 | 27.6343 | -8.2259 | 1.733515 |  | -4.74521 | 2.08E-06 | 0.000439 | YEATS2 |
| MSTRG.12022.1 | 96.1348 | 2.199236 | 0.686666 |  | 3.202774 | 0.001361 | 0.084411 | SEPTIN2 |
| MSTRG.12047.3 | 53.18624 | 1.283139 | 0.59241 |  | 2.165965 | 0.030314 | 0.533662 | UBXN7 |
| MSTRG.12103.11 | 480.2044 | 1.638756 | 0.478902 |  | 3.4219 | 0.000622 | 0.047141 | STAG1 |
| MSTRG.12212.4 | 291.4549 | -1.94298 | 0.730787 |  | -2.65875 | 0.007843 | 0.257946 | TRIP12 |
| MSTRG.12284.2 | 1817.753 | 1.533549 | 0.372276 |  | 4.119393 | 3.80E-05 | 0.005146 | STAG2 |
| MSTRG.12307.7 | 72.12497 | -1.27636 | 0.476427 |  | -2.67903 | 0.007384 | 0.250224 | LOC113840926 |
| MSTRG.1233.19 | 204.7462 | 6.023159 | 1.719767 |  | 3.502312 | 0.000461 | 0.037142 | PPFIBP1 |
| MSTRG.1234.4 | 28.30405 | -8.25995 | 3.283249 |  | -2.51578 | 0.011877 | 0.327726 | ARNTL2 |
| MSTRG.12368.5 | 18.21889 | -7.62457 | 3.44632 |  | -2.21238 | 0.02694 | 0.504973 | ATRX |
| MSTRG.12444.1 | 118.64 | 2.279614 | 0.452578 |  | 5.036955 | 4.73E-07 | 0.00013 | LOC101794183 |
| MSTRG.12449.7 | 1277.517 | 1.108891 | 0.383406 |  | 2.892214 | 0.003825 | 0.165793 | LOC119717843 |
| MSTRG.12456.15 | 424.2906 | 1.190637 | 0.495714 |  | 2.401863 | 0.016312 | 0.389679 | LOC119717844 |
| MSTRG.12635.4 | 1769.707 | -1.15753 | 0.429089 |  | -2.69765 | 0.006983 | 0.243791 | DKC1 |
| MSTRG.12647.1 | 573.546 | -12.6009 | 1.438025 |  | -8.76265 | 1.91E-18 | 4.77E-15 | TAF1 |
| MSTRG.12702.4 | 92.26039 | 4.860794 | 0.639906 |  | 7.596104 | 3.05E-14 | 4.57E-11 | PHF6 |
| MSTRG.12773.5 | 587.7311 | 1.276894 | 0.636887 |  | 2.004898 | 0.044974 | 0.623498 | LOC101797884 |
| MSTRG.12817.1 | 1125.79 | -1.73254 | 0.842011 |  | -2.05762 | 0.039627 | 0.594241 | EFNB1 |
| MSTRG.12866.3 | 7.59002 | 6.403857 | 2.288832 |  | 2.797872 | 0.005144 | 0.197777 | SEMA6D |
| MSTRG.12934.1 | 31.12212 | -8.39691 | 1.897226 |  | -4.42589 | 9.60E-06 | 0.001669 | PIGB |
| MSTRG.12946.10 | 133.5126 | 10.54094 | 2.835735 |  | 3.717182 | 0.000201 | 0.019496 | TCF12 |
| MSTRG.12952.6 | 287.9975 | -1.7977 | 0.726911 |  | -2.47307 | 0.013396 | 0.351553 | LOC101801563 |
| MSTRG.12955.3 | 143.2768 | -1.79788 | 0.714768 |  | -2.51533 | 0.011892 | 0.327726 | ALDH1A2 |
| MSTRG.13077.1 | 63.50289 | 9.469182 | 1.435067 |  | 6.598423 | 4.16E-11 | 2.89E-08 | LINGO1 |
| MSTRG.13128.1 | 1490.092 | -1.25847 | 0.437875 |  | -2.87404 | 0.004053 | 0.171688 | ARIH1 |
| MSTRG.13281.10 | 1256.827 | -2.02002 | 0.671637 |  | -3.0076 | 0.002633 | 0.128995 | NR2F2 |
| MSTRG.13281.11 | 470.799 | -1.86808 | 0.597025 |  | -3.12898 | 0.001754 | 0.099714 | NR2F2 |
| MSTRG.13281.9 | 308.4072 | -1.29605 | 0.59259 |  | -2.1871 | 0.028735 | 0.523696 | NR2F2 |
| MSTRG.13287.2 | 160.5892 | -1.18202 | 0.522125 |  | -2.26386 | 0.023583 | 0.472675 | ARRDC4 |
| MSTRG.13291.5 | 281.5677 | -1.60717 | 0.779797 |  | -2.06101 | 0.039302 | 0.592771 | IGF1R |
| MSTRG.13346.2 | 65.92787 | 1.037451 | 0.497964 |  | 2.083385 | 0.037216 | 0.579715 | ZWILCH |
| MSTRG.13379.2 | 145.4864 | 1.408564 | 0.580225 |  | 2.427616 | 0.015198 | 0.373735 | GLCE |
| MSTRG.13397.1 | 405.339 | -1.61186 | 0.719513 |  | -2.24022 | 0.025077 | 0.489092 | CTDSPL2 |
| MSTRG.13455.2 | 526.2555 | -1.18106 | 0.473962 |  | -2.49189 | 0.012707 | 0.340156 | AP1G1 |
| MSTRG.13532.5 | 68.28984 | -9.53069 | 1.862642 |  | -5.11676 | 3.11E-07 | 9.04E-05 | CTU2 |
| MSTRG.13600.1 | 15.10965 | 7.397986 | 1.923052 |  | 3.847002 | 0.00012 | 0.013158 | LOC101791461 |
| MSTRG.13739.12 | 138.1283 | 1.352549 | 0.663757 |  | 2.037718 | 0.041578 | 0.605287 | CENPT |
| MSTRG.13775.2 | 181.8973 | 1.827998 | 0.832643 |  | 2.195417 | 0.028134 | 0.516561 | SLC9A5 |
| MSTRG.13809.2 | 97.67477 | 1.423651 | 0.666771 |  | 2.135142 | 0.032749 | 0.549611 | ST3GAL2 |
| MSTRG.13810.4 | 178.0731 | 1.572503 | 0.663277 |  | 2.370811 | 0.017749 | 0.408889 | PDPR |
| MSTRG.1396.5 | 119.5079 | -2.53702 | 1.144347 |  | -2.217 | 0.026623 | 0.502497 | KEL |
| MSTRG.14035.13 | 2602.077 | 1.089745 | 0.201331 |  | 5.4127 | 6.21E-08 | 2.16E-05 | FOXP1 |
| MSTRG.14035.27 | 696.4994 | -1.75873 | 0.616475 |  | -2.85287 | 0.004333 | 0.177296 | FOXP1 |
| MSTRG.14100.3 | 981.1533 | -3.3851 | 1.06322 |  | -3.18382 | 0.001453 | 0.08797 | LOC119718225 |
| MSTRG.14117.2 | 17.89055 | -7.59819 | 3.57311 |  | -2.12649 | 0.033462 | 0.55438 | PDHB |
| MSTRG.14192.9 | 72.03454 | 2.721237 | 1.357013 |  | 2.005314 | 0.04493 | 0.623498 | SLC41A3 |
| MSTRG.14218.7 | 247.9161 | -1.05693 | 0.530777 |  | -1.9913 | 0.046448 | 0.6322 | GATA2 |
| MSTRG.14277.1 | 32.97494 | -1.31486 | 0.640767 |  | -2.052 | 0.040169 | 0.596426 | SEMA3B |
| MSTRG.14397.3 | 148.5394 | 1.120924 | 0.417137 |  | 2.687185 | 0.007206 | 0.248296 | RNF123 |
| MSTRG.14495.1 | 137.8044 | -1.5973 | 0.71484 |  | -2.23449 | 0.025451 | 0.492307 | ACTR8 |
| MSTRG.14541.2 | 172.404 | 1.506314 | 0.644463 |  | 2.337318 | 0.019423 | 0.428223 | TCERG1 |
| MSTRG.14548.2 | 57.53078 | -2.35079 | 1.001838 |  | -2.34647 | 0.018952 | 0.423411 | ARHGAP26 |
| MSTRG.14611.8 | 70.07946 | -7.01107 | 2.560541 |  | -2.73812 | 0.006179 | 0.222527 | LOC101795967 |
| MSTRG.14669.1 | 171.7762 | -3.70894 | 0.996427 |  | -3.72224 | 0.000197 | 0.019196 | CD74 |
| MSTRG.14827.2 | 79.23874 | 9.78865 | 1.456161 |  | 6.72223 | 1.79E-11 | 1.40E-08 | DUSP1 |
| MSTRG.14856.3 | 11.94034 | 4.175535 | 1.843373 |  | 2.265159 | 0.023503 | 0.471881 | HARS1 |
| MSTRG.14907.3 | 198.0032 | 1.774702 | 0.771319 |  | 2.300868 | 0.021399 | 0.449106 | DNAJC18 |
| MSTRG.14943.2 | 1211.027 | 1.178488 | 0.568876 |  | 2.071609 | 0.038302 | 0.588085 | DOCK2 |
| MSTRG.1503.3 | 106.7777 | -1.36467 | 0.576976 |  | -2.36521 | 0.01802 | 0.412733 | CHD1L |
| MSTRG.15037.11 | 293.7308 | 1.343322 | 0.65923 |  | 2.037714 | 0.041579 | 0.605287 | CIITA |
| MSTRG.15108.2 | 179.5967 | 1.419476 | 0.527869 |  | 2.689068 | 0.007165 | 0.247553 | NAGPA |
| MSTRG.15175.4 | 36.32941 | 1.533998 | 0.758583 |  | 2.022189 | 0.043157 | 0.615901 | HMOX2 |
| MSTRG.15238.3 | 46.8777 | 3.204259 | 1.473249 |  | 2.174961 | 0.029633 | 0.529453 | RPS15A |
| MSTRG.15243.6 | 50.86152 | -9.10558 | 3.013787 |  | -3.02131 | 0.002517 | 0.125591 | TMC7 |
| MSTRG.15286.6 | 22.38587 | -7.9217 | 1.910105 |  | -4.14726 | 3.36E-05 | 0.004676 | CYTH3 |
| MSTRG.15324.2 | 507.6117 | 1.285004 | 0.534142 |  | 2.405734 | 0.01614 | 0.387247 | GPER1 |
| MSTRG.1537.1 | 118.9958 | -1.41831 | 0.509316 |  | -2.78473 | 0.005357 | 0.203832 | CLDND1 |
| MSTRG.15444.8 | 426.5298 | 1.086755 | 0.444878 |  | 2.442818 | 0.014573 | 0.367263 | PRPSAP2 |
| MSTRG.15518.1 | 87.44684 | 9.930407 | 2.854139 |  | 3.479301 | 0.000503 | 0.039745 | SCNN1B |
| MSTRG.15536.1 | 82.7696 | -1.37131 | 0.520112 |  | -2.63657 | 0.008375 | 0.268377 | GNPTG |
| MSTRG.15579.1 | 155.4342 | 10.76058 | 1.446594 |  | 7.438562 | 1.02E-13 | 1.32E-10 | KCTD10 |
| MSTRG.15636.13 | 175.7104 | 10.93704 | 1.398087 |  | 7.822862 | 5.16E-15 | 9.23E-12 | CLIP1 |
| MSTRG.15707.4 | 1059.702 | -1.438 | 0.502358 |  | -2.8625 | 0.004203 | 0.174278 | UBC |
| MSTRG.15754.1 | 4.922503 | -5.73619 | 2.684441 |  | -2.13683 | 0.032612 | NA | EWSR1 |
| MSTRG.15763.2 | 66.25541 | 3.518288 | 1.540135 |  | 2.284402 | 0.022348 | 0.461279 | DTX1 |
| MSTRG.15928.2 | 33.56519 | -1.65553 | 0.712082 |  | -2.32492 | 0.020076 | 0.435954 | TOP3B |
| MSTRG.15988.9 | 176.2059 | 10.9411 | 2.065182 |  | 5.297883 | 1.17E-07 | 3.82E-05 | EP400 |
| MSTRG.15996.1 | 34.57808 | 8.592035 | 3.168 |  | 2.712132 | 0.006685 | 0.235772 | LOC101795909 |
| MSTRG.1602.4 | 22.90599 | 7.997523 | 3.355668 |  | 2.383288 | 0.017159 | 0.400425 | LOC101801420 |
| MSTRG.16031.14 | 152.5504 | -2.39794 | 0.726297 |  | -3.3016 | 0.000961 | 0.06583 | LOC119718653 |
| MSTRG.16031.5 | 413.1856 | -1.67742 | 0.748568 |  | -2.24084 | 0.025036 | 0.488805 | LOC119718652 |
| MSTRG.16031.7 | 252.9022 | -2.16295 | 0.387333 |  | -5.58422 | 2.35E-08 | 9.18E-06 | LOC113841252 |
| MSTRG.16189.1 | 355.623 | 2.286926 | 1.067635 |  | 2.142049 | 0.03219 | 0.546883 | LOC101800226 |
| MSTRG.16189.2 | 94.34864 | 3.973665 | 1.948516 |  | 2.039329 | 0.041417 | 0.604504 | LOC101800226 |
| MSTRG.16232.9 | 27.51862 | 8.262624 | 3.296163 |  | 2.50674 | 0.012185 | 0.332885 | RGS3 |
| MSTRG.16334.2 | 529.6765 | -1.24143 | 0.541657 |  | -2.29191 | 0.021911 | 0.455817 | RXRA |
| MSTRG.16381.15 | 282.4376 | 1.053014 | 0.424964 |  | 2.477888 | 0.013216 | 0.349254 | PRRC2B |
| MSTRG.16399.6 | 709.9991 | 1.034514 | 0.468763 |  | 2.206901 | 0.027321 | 0.509013 | FNBP1 |
| MSTRG.16402.3 | 40.2224 | -6.97553 | 2.638482 |  | -2.64377 | 0.008199 | 0.265737 | USP20 |
| MSTRG.16462.1 | 106.2211 | 1.353509 | 0.674197 |  | 2.007586 | 0.044687 | 0.623412 | GOLGA2 |
| MSTRG.165.2 | 4942.203 | 1.822652 | 0.894223 |  | 2.038252 | 0.041525 | 0.605287 | SEMA3C |
| MSTRG.16519.4 | 246.3697 | 1.859029 | 0.430554 |  | 4.317757 | 1.58E-05 | 0.002485 | RABGAP1 |
| MSTRG.16565.20 | 43.72306 | -3.12882 | 1.237589 |  | -2.52816 | 0.011466 | 0.322573 | RALGPS1 |
| MSTRG.1659.4 | 183.5839 | 1.869148 | 0.879766 |  | 2.124596 | 0.03362 | 0.554394 | CD96 |
| MSTRG.16628.5 | 74.82091 | 2.431698 | 0.65141 |  | 3.732977 | 0.000189 | 0.018492 | TBC1D16 |
| MSTRG.16633.1 | 14.58137 | -7.30302 | 3.601376 |  | -2.02784 | 0.042576 | 0.611964 | RNF213 |
| MSTRG.16667.3 | 21.25925 | 7.890172 | 3.455108 |  | 2.283625 | 0.022394 | 0.461953 | COG1 |
| MSTRG.16879.2 | 330.2417 | 1.265771 | 0.578471 |  | 2.188134 | 0.02866 | 0.523084 | RECQL5 |
| MSTRG.16917.1 | 194.1118 | -11.038 | 1.559475 |  | -7.07801 | 1.46E-12 | 1.44E-09 | RAB37 |
| MSTRG.16973.23 | 145.7493 | 1.620613 | 0.793846 |  | 2.04147 | 0.041204 | 0.603946 | RAP1GAP2 |
| MSTRG.17057.4 | 26.81044 | 2.221268 | 0.878714 |  | 2.527862 | 0.011476 | 0.322573 | TUBD1 |
| MSTRG.1708.1 | 1314.659 | -1.24313 | 0.538386 |  | -2.309 | 0.020944 | 0.445527 | CREG1 |
| MSTRG.17095.29 | 87.43611 | 5.249337 | 1.004496 |  | 5.225839 | 1.73E-07 | 5.47E-05 | LOC119713271 |
| MSTRG.17095.43 | 38.69388 | 8.75425 | 3.326851 |  | 2.631392 | 0.008504 | 0.271186 | LOC113839590 |
| MSTRG.17095.44 | 33.21699 | 8.534342 | 1.888232 |  | 4.519753 | 6.19E-06 | 0.001138 | LOC119713271 |
| MSTRG.17095.46 | 33.21699 | 8.534342 | 1.888232 |  | 4.519753 | 6.19E-06 | 0.001138 | LOC113839590 |
| MSTRG.17120.6 | 1213.739 | 3.597845 | 1.102091 |  | 3.264561 | 0.001096 | 0.072176 | NCOR1 |
| MSTRG.17120.7 | 915.8322 | -2.20675 | 1.055775 |  | -2.09017 | 0.036602 | 0.574447 | NCOR1 |
| MSTRG.17162.2 | 19.87578 | -7.74989 | 3.417112 |  | -2.26796 | 0.023331 | 0.469958 | DHRS13 |
| MSTRG.17182.4 | 43.72545 | -8.88744 | 1.491386 |  | -5.95918 | 2.54E-09 | 1.36E-06 | KIAA0100 |
| MSTRG.17292.11 | 1178.555 | 1.111293 | 0.525085 |  | 2.116405 | 0.03431 | 0.558083 | CUX1 |
| MSTRG.17309.5 | 184.4851 | -1.0688 | 0.368107 |  | -2.90351 | 0.00369 | 0.162144 | UBE2G1 |
| MSTRG.17468.12 | 182.6843 | 1.154707 | 0.56185 |  | 2.055187 | 0.039861 | 0.595114 | ZMYND8 |
| MSTRG.17543.2 | 258.3143 | 1.032043 | 0.487614 |  | 2.116518 | 0.034301 | 0.558083 | MYBL2 |
| MSTRG.17561.8 | 1772.281 | 1.140383 | 0.268107 |  | 4.253458 | 2.10E-05 | 0.003224 | ZHX3 |
| MSTRG.17575.2 | 1713.014 | 1.182784 | 0.453443 |  | 2.608448 | 0.009095 | 0.282466 | SDC4 |
| MSTRG.17577.1 | 49.5337 | 5.398879 | 2.71742 |  | 1.986766 | 0.046948 | 0.636006 | LOC101796317 |
| MSTRG.17584.1 | 205.2265 | 11.16102 | 3.906977 |  | 2.85669 | 0.004281 | 0.176138 | LAMA5 |
| MSTRG.17608.1 | 38.64253 | 2.137065 | 0.712367 |  | 2.99995 | 0.0027 | 0.130913 | GNAS |
| MSTRG.17617.2 | 54.58055 | -9.20744 | 2.989959 |  | -3.07945 | 0.002074 | 0.109915 | LOC101805171 |
| MSTRG.17655.4 | 2414.982 | 1.013267 | 0.424468 |  | 2.387146 | 0.01698 | 0.398586 | NFATC2 |
| MSTRG.17669.2 | 349.3448 | 2.392723 | 0.880067 |  | 2.718797 | 0.006552 | 0.232825 | PTPN1 |
| MSTRG.1772.2 | 493.6891 | 3.54952 | 1.002297 |  | 3.541385 | 0.000398 | 0.033118 | FOSB |
| MSTRG.1772.3 | 37.59265 | 8.71254 | 3.125427 |  | 2.787632 | 0.005309 | 0.202273 | FOSB |
| MSTRG.17761.4 | 226.0312 | -1.34275 | 0.572559 |  | -2.34518 | 0.019018 | 0.424031 | HELZ2 |
| MSTRG.17764.1 | 15.08819 | 5.478814 | 1.667888 |  | 3.284882 | 0.00102 | 0.068123 | LOC113845767 |
| MSTRG.17817.8 | 58.19765 | 5.003126 | 2.151559 |  | 2.325349 | 0.020053 | 0.435727 | LOC101799803 |
| MSTRG.17819.1 | 964.428 | -1.03517 | 0.272687 |  | -3.79619 | 0.000147 | 0.015232 | TGM2 |
| MSTRG.17932.2 | 213.6396 | -1.07024 | 0.417255 |  | -2.56494 | 0.010319 | 0.306113 | CHD5 |
| MSTRG.17937.3 | 56.4333 | -1.73988 | 0.727757 |  | -2.39075 | 0.016814 | 0.396324 | GPR153 |
| MSTRG.17946.3 | 801.9883 | 1.009326 | 0.415899 |  | 2.426853 | 0.01523 | 0.374179 | NOL9 |
| MSTRG.17976.2 | 320.4054 | 1.334373 | 0.564542 |  | 2.363637 | 0.018097 | 0.413564 | LOC101789439 |
| MSTRG.17991.5 | 141.5659 | 2.075158 | 0.901987 |  | 2.300651 | 0.021411 | 0.449112 | ACAP3 |
| MSTRG.18048.3 | 114.4911 | -1.20289 | 0.558749 |  | -2.15283 | 0.031332 | 0.541615 | MTOR |
| MSTRG.18135.2 | 71.86975 | 1.278816 | 0.606945 |  | 2.10697 | 0.03512 | 0.563439 | ATP13A2 |
| MSTRG.18155.1 | 258.5389 | -5.06867 | 1.214743 |  | -4.17263 | 3.01E-05 | 0.004329 | PLEKHM2 |
| MSTRG.18275.1 | 157.9978 | 10.78387 | 2.713712 |  | 3.973843 | 7.07E-05 | 0.00873 | MAK16 |
| MSTRG.18283.5 | 120.5423 | -2.44531 | 1.171924 |  | -2.08658 | 0.036926 | 0.576875 | DMTN |
| MSTRG.18314.2 | 241.4254 | 1.701818 | 0.551148 |  | 3.087769 | 0.002017 | 0.107735 | LOXL2 |
| MSTRG.18379.4 | 438.2095 | 1.3931 | 0.660399 |  | 2.109482 | 0.034903 | 0.560914 | LOC119713470 |
| MSTRG.18660.4 | 133.626 | -1.64735 | 0.804116 |  | -2.04864 | 0.040497 | 0.59923 | LOC101804293 |
| MSTRG.1869.12 | 749.0128 | 2.364499 | 0.811574 |  | 2.913471 | 0.003574 | 0.159062 | SON |
| MSTRG.18787.2 | 138.5075 | -1.32033 | 0.624441 |  | -2.11443 | 0.034479 | 0.558247 | ZBTB44 |
| MSTRG.18810.6 | 149.3547 | 3.220467 | 1.193334 |  | 2.698713 | 0.006961 | 0.243434 | POU2AF1 |
| MSTRG.18887.5 | 2659.629 | 2.795448 | 0.676125 |  | 4.134515 | 3.56E-05 | 0.004891 | ZBTB16 |
| MSTRG.19022.8 | 54.30007 | 4.260666 | 1.634418 |  | 2.606839 | 0.009138 | 0.282711 | CADM3 |
| MSTRG.19058.13 | 79.87532 | -4.06625 | 1.110354 |  | -3.66212 | 0.00025 | 0.023006 | ARNT |
| MSTRG.19109.3 | 354.6871 | -3.13085 | 0.464922 |  | -6.73414 | 1.65E-11 | 1.32E-08 | SLC27A3 |
| MSTRG.1912.2 | 103.0006 | -3.53056 | 1.229482 |  | -2.87159 | 0.004084 | 0.172272 | VPS26C |
| MSTRG.19165.2 | 54.85809 | 1.665046 | 0.825552 |  | 2.016886 | 0.043707 | 0.618371 | RUSC1 |
| MSTRG.19358.12 | 66.38905 | 9.532897 | 2.952282 |  | 3.228993 | 0.001242 | 0.078822 | LOC101793778 |
| MSTRG.19358.15 | 322.4104 | 11.81271 | 3.906898 |  | 3.023552 | 0.002498 | 0.125498 | LOC101793957 |
| MSTRG.19358.22 | 144.8078 | 10.65799 | 2.77962 |  | 3.834333 | 0.000126 | 0.013616 | LOC101793957 |
| MSTRG.19358.4 | 56.99474 | 6.069376 | 2.277521 |  | 2.664904 | 0.007701 | 0.255667 | LOC101793957 |
| MSTRG.19358.8 | 48.42133 | 9.077549 | 3.033427 |  | 2.992506 | 0.002767 | 0.133116 | LOC101793957 |
| MSTRG.19395.1 | 1765.746 | 4.398013 | 1.04082 |  | 4.225525 | 2.38E-05 | 0.003564 | CSRP1 |
| MSTRG.19462.1 | 6934.797 | 2.498709 | 0.825115 |  | 3.028315 | 0.002459 | 0.123868 | CR1 |
| MSTRG.19462.11 | 22.86314 | 3.85906 | 1.888948 |  | 2.042968 | 0.041056 | 0.602743 | CR1 |
| MSTRG.19462.2 | 1421.036 | 3.158795 | 1.227745 |  | 2.572844 | 0.010087 | 0.301633 | CR1 |
| MSTRG.19462.3 | 1939.048 | 2.626695 | 0.786147 |  | 3.341227 | 0.000834 | 0.058944 | CR1 |
| MSTRG.19513.1 | 175.5376 | -1.18224 | 0.533921 |  | -2.21427 | 0.02681 | 0.503785 | ZNF652 |
| MSTRG.19538.5 | 43.52744 | -1.26472 | 0.549471 |  | -2.30171 | 0.021352 | 0.448759 | SKAP1 |
| MSTRG.1966.6 | 19.2242 | -7.70196 | 3.462336 |  | -2.2245 | 0.026115 | 0.498865 | TAB3 |
| MSTRG.19699.3 | 24.5269 | 8.096599 | 3.350775 |  | 2.416336 | 0.015678 | 0.380285 | ACLY |
| MSTRG.19706.2 | 4527.542 | -1.14928 | 0.348777 |  | -3.29518 | 0.000984 | 0.066383 | LOC101792592 |
| MSTRG.19727.2 | 1097.349 | 13.57974 | 3.9068 |  | 3.475923 | 0.000509 | 0.040134 | LOC101792081 |
| MSTRG.19763.18 | 18.05102 | 2.278741 | 1.059624 |  | 2.150519 | 0.031514 | 0.542463 | LOC106020188 |
| MSTRG.19763.33 | 142.3249 | 2.877188 | 1.039148 |  | 2.768794 | 0.005626 | 0.210081 | LOC106020188 |
| MSTRG.19763.5 | 362.8103 | 2.544509 | 0.814114 |  | 3.125497 | 0.001775 | 0.099714 | LOC106020188 |
| MSTRG.19763.6 | 73.02311 | 1.646016 | 0.819518 |  | 2.008517 | 0.044588 | 0.623056 | LOC106020188 |
| MSTRG.19844.5 | 16.54616 | -5.01077 | 1.662282 |  | -3.01439 | 0.002575 | 0.127473 | GRN |
| MSTRG.19856.1 | 28.42984 | -8.26685 | 1.866664 |  | -4.42867 | 9.48E-06 | 0.001655 | FTSJ3 |
| MSTRG.19869.10 | 44.49473 | 7.083563 | 1.369923 |  | 5.170773 | 2.33E-07 | 7.05E-05 | LOC101804703 |
| MSTRG.19887.3 | 230.097 | -1.41046 | 0.353438 |  | -3.99068 | 6.59E-05 | 0.008186 | LOC101804149 |
| MSTRG.19994.1 | 149.6151 | -2.03529 | 0.732889 |  | -2.77707 | 0.005485 | 0.205623 | SUGP2 |
| MSTRG.20068.1 | 4896.712 | -1.61497 | 0.271255 |  | -5.95371 | 2.62E-09 | 1.39E-06 | PTPRS |
| MSTRG.20068.6 | 105.9049 | -9.19973 | 2.479011 |  | -3.71105 | 0.000206 | 0.019911 | PTPRS |
| MSTRG.20116.6 | 259.9854 | 11.50253 | 1.578019 |  | 7.289225 | 3.12E-13 | 3.77E-10 | HNRNPM |
| MSTRG.20128.2 | 578.9698 | 1.313228 | 0.663926 |  | 1.977974 | 0.047932 | 0.641453 | LMNB2 |
| MSTRG.20291.2 | 643.0884 | 2.350552 | 1.07466 |  | 2.187253 | 0.028724 | 0.523696 | CD22 |
| MSTRG.2032.15 | 4840.662 | -2.21728 | 0.588568 |  | -3.76725 | 0.000165 | 0.01674 | LOC119714884 |
| MSTRG.20625.3 | 132.3731 | 1.098211 | 0.438447 |  | 2.504775 | 0.012253 | 0.332941 | LMAN1 |
| MSTRG.20643.12 | 1014.737 | 1.636169 | 0.827099 |  | 1.978203 | 0.047906 | 0.641453 | TCF4 |
| MSTRG.20643.40 | 799.9439 | -1.77642 | 0.548891 |  | -3.23639 | 0.001211 | 0.077253 | TCF4 |
| MSTRG.20643.8 | 562.2191 | -1.92033 | 0.879701 |  | -2.18293 | 0.029041 | 0.527213 | TCF4 |
| MSTRG.20696.7 | 110.4495 | 1.141968 | 0.510935 |  | 2.235055 | 0.025414 | 0.492169 | CZH18orf25 |
| MSTRG.20719.2 | 29.13419 | -7.3324 | 2.671291 |  | -2.74489 | 0.006053 | 0.219886 | UBAP2 |
| MSTRG.20719.21 | 57.95504 | 9.3366 | 2.190582 |  | 4.262155 | 2.02E-05 | 0.003114 | UBAP2 |
| MSTRG.20777.1 | 33.80895 | 3.892872 | 1.7875 |  | 2.177831 | 0.029419 | 0.528936 | LOC119714450 |
| MSTRG.20782.1 | 47.62785 | 3.875971 | 1.921861 |  | 2.01678 | 0.043719 | 0.618371 | NPR2 |
| MSTRG.2081.10 | 27.47737 | -8.21725 | 3.351812 |  | -2.45158 | 0.014223 | 0.362662 | PRDM15 |
| MSTRG.20851.5 | 538.8412 | 1.054853 | 0.415331 |  | 2.539788 | 0.011092 | 0.316522 | RICTOR |
| MSTRG.20911.4 | 7849.289 | 1.245014 | 0.347873 |  | 3.578936 | 0.000345 | 0.029898 | PLPP1 |
| MSTRG.21082.11 | 198.4537 | 1.298488 | 0.610158 |  | 2.128118 | 0.033327 | 0.553366 | JAK2 |
| MSTRG.21182.3 | 224.8471 | -1.76357 | 0.451499 |  | -3.90604 | 9.38E-05 | 0.010968 | FXN |
| MSTRG.21199.5 | 512.0127 | 1.388932 | 0.474367 |  | 2.927969 | 0.003412 | 0.153881 | APBA1 |
| MSTRG.21246.9 | 221.4262 | 1.371203 | 0.695878 |  | 1.970464 | 0.048785 | 0.646539 | UBQLN1 |
| MSTRG.21293.3 | 105.912 | 1.710738 | 0.737915 |  | 2.318341 | 0.020431 | 0.439085 | LOC101795007 |
| MSTRG.21411.3 | 530.5669 | 1.326492 | 0.425922 |  | 3.114398 | 0.001843 | 0.101865 | LOC101800068 |
| MSTRG.21489.2 | 225.1943 | -1.45177 | 0.482656 |  | -3.00787 | 0.002631 | 0.128995 | LOC119714387 |
| MSTRG.21562.7 | 852.9459 | -1.18499 | 0.538059 |  | -2.20235 | 0.027641 | 0.511702 | SEMA6A |
| MSTRG.21668.4 | 19.74416 | -7.74037 | 3.541642 |  | -2.18553 | 0.02885 | 0.525274 | SNX30 |
| MSTRG.21755.10 | 1173.979 | 1.22136 | 0.356339 |  | 3.427529 | 0.000609 | 0.046376 | CAST |
| MSTRG.21771.10 | 509.3098 | 3.606721 | 0.996343 |  | 3.619958 | 0.000295 | 0.026228 | PAX5 |
| MSTRG.21781.3 | 57.62237 | -2.31786 | 0.75875 |  | -3.05484 | 0.002252 | 0.115724 | POLR1E |
| MSTRG.21836.2 | 77.21588 | 1.424783 | 0.625109 |  | 2.279253 | 0.022652 | 0.463469 | PAN2 |
| MSTRG.22020.2 | 86.08114 | -9.86465 | 3.086769 |  | -3.19578 | 0.001395 | 0.085365 | GABBR1 |
| MSTRG.22214.1 | 9.70003 | -4.8636 | 1.787899 |  | -2.72029 | 0.006523 | 0.232438 | LOC119714934 |
| MSTRG.22297.2 | 254.7284 | 2.20117 | 0.878181 |  | 2.506511 | 0.012193 | 0.332885 | LOC119715007 |
| MSTRG.22297.4 | 277.5892 | 4.813482 | 1.954833 |  | 2.46235 | 0.013803 | 0.358006 | LOC119715007 |
| MSTRG.2235.6 | 19.13108 | 7.737561 | 3.498326 |  | 2.21179 | 0.026981 | 0.504973 | NIPA2 |
| MSTRG.22390.3 | 66.36882 | 2.626272 | 0.531325 |  | 4.942871 | 7.70E-07 | 0.000193 | LOC119713017 |
| MSTRG.22390.5 | 260.7252 | 1.98029 | 0.650328 |  | 3.045065 | 0.002326 | 0.11893 | LOC119713017 |
| MSTRG.22398.3 | 1571.865 | 2.487236 | 1.145923 |  | 2.170509 | 0.029968 | 0.531708 | LOC119715071 |
| MSTRG.22439.8 | 592.9275 | 1.849294 | 0.391681 |  | 4.721435 | 2.34E-06 | 0.000488 | LOC119715087 |
| MSTRG.22589.6 | 152.7307 | -1.30647 | 0.62238 |  | -2.09915 | 0.035803 | 0.568798 | LOC119715259 |
| MSTRG.2260.1 | 68.10711 | -2.19783 | 1.11591 |  | -1.96954 | 0.048891 | 0.647097 | UNC50 |
| MSTRG.22665.2 | 1080.004 | -1.0888 | 0.177461 |  | -6.13539 | 8.49E-10 | 4.95E-07 | ACVRL1 |
| MSTRG.22695.1 | 435.3252 | -12.2029 | 3.906864 |  | -3.12345 | 0.001787 | 0.099967 | LOC101794328 |
| MSTRG.22710.2 | 123.6674 | -1.26298 | 0.634023 |  | -1.992 | 0.046371 | 0.631857 | LOC113840667 |
| MSTRG.22812.3 | 19.12069 | 7.737111 | 1.950998 |  | 3.96572 | 7.32E-05 | 0.008973 | RUSF1 |
| MSTRG.22910.2 | 200.9568 | 1.9293 | 0.949415 |  | 2.032094 | 0.042144 | 0.608722 | LOC119715490 |
| MSTRG.2295.28 | 15.0351 | -7.34753 | 3.655105 |  | -2.01021 | 0.044409 | 0.622469 | MAP4K4 |
| MSTRG.2327.7 | 1091.85 | -1.43283 | 0.687492 |  | -2.08414 | 0.037147 | 0.579715 | LIMS1 |
| MSTRG.2356.4 | 15.18792 | -7.36201 | 3.560297 |  | -2.06781 | 0.038658 | 0.590326 | LOC101795284 |
| MSTRG.2383.2 | 1329.472 | -4.10373 | 1.885413 |  | -2.17657 | 0.029513 | 0.528936 | COL4A2 |
| MSTRG.2390.11 | 405.5658 | 1.226808 | 0.603938 |  | 2.031349 | 0.04222 | 0.609108 | ANKRD10 |
| MSTRG.2390.7 | 139.9507 | 1.350268 | 0.550217 |  | 2.454064 | 0.014125 | 0.362207 | ANKRD10 |
| MSTRG.2399.2 | 152.1368 | -1.02575 | 0.48731 |  | -2.10493 | 0.035297 | 0.564349 | ARGLU1 |
| MSTRG.2481.1 | 1522.513 | 1.077452 | 0.359134 |  | 3.000141 | 0.002699 | 0.130913 | RBM26 |
| MSTRG.2490.24 | 784.1129 | -1.8491 | 0.560849 |  | -3.29696 | 0.000977 | 0.06618 | MYCBP2 |
| MSTRG.2497.27 | 424.4039 | 3.989229 | 1.540388 |  | 2.589756 | 0.009604 | 0.29159 | LMO7 |
| MSTRG.2522.11 | 147.3304 | 2.047648 | 0.686371 |  | 2.983294 | 0.002852 | 0.135453 | DACH1 |
| MSTRG.2554.1 | 1577.986 | -1.16959 | 0.447286 |  | -2.61485 | 0.008927 | 0.279145 | RGCC |
| MSTRG.2554.2 | 245.2073 | -1.20306 | 0.368313 |  | -3.26639 | 0.001089 | 0.071963 | RGCC |
| MSTRG.2584.2 | 33.80269 | 5.048229 | 2.21639 |  | 2.277681 | 0.022746 | 0.464379 | LOC119716819 |
| MSTRG.2584.6 | 14.59111 | -7.3041 | 3.578108 |  | -2.04133 | 0.041218 | 0.603946 | LOC119716819 |
| MSTRG.2589.1 | 25.76968 | 2.050671 | 1.020532 |  | 2.009414 | 0.044493 | 0.622486 |  |
| MSTRG.2593.3 | 72.04987 | 3.266339 | 0.751457 |  | 4.346672 | 1.38E-05 | 0.002236 | LOC101792784 |
| MSTRG.2600.1 | 369.1072 | 10.17255 | 1.42893 |  | 7.118997 | 1.09E-12 | 1.10E-09 | ZC3H13 |
| MSTRG.2626.3 | 167.0351 | 1.422539 | 0.602039 |  | 2.362867 | 0.018134 | 0.41392 | LOC101790074 |
| MSTRG.2670.3 | 67.94966 | 7.718486 | 1.278736 |  | 6.036025 | 1.58E-09 | 8.85E-07 | SUPT20H |
| MSTRG.2797.1 | 281.4983 | 1.680187 | 0.597773 |  | 2.810744 | 0.004943 | 0.193477 | ZC3H12C |
| MSTRG.2895.1 | 305.2242 | 1.08254 | 0.517673 |  | 2.091163 | 0.036513 | 0.574047 | CHORDC1 |
| MSTRG.2962.1 | 100.0845 | -10.0821 | 2.828133 |  | -3.56494 | 0.000364 | 0.031109 | TENM4 |
| MSTRG.2962.21 | 567.687 | -7.93448 | 3.745251 |  | -2.11854 | 0.034129 | 0.55643 | TENM4 |
| MSTRG.2992.2 | 81.40506 | 9.827161 | 2.893216 |  | 3.396623 | 0.000682 | 0.050605 | EMSY |
| MSTRG.3020.3 | 545.9936 | 1.252819 | 0.350967 |  | 3.569622 | 0.000357 | 0.030725 | RAB6A |
| MSTRG.3043.2 | 4.685407 | -5.66549 | 2.698682 |  | -2.09935 | 0.035786 | NA | LAMTOR1 |
| MSTRG.3128.2 | 199.335 | -11.076 | 3.906982 |  | -2.83493 | 0.004584 | 0.18415 | PLEC |
| MSTRG.313.2 | 153.2871 | -10.6974 | 1.317047 |  | -8.12228 | 4.57E-16 | 8.58E-13 | LOC101800968 |
| MSTRG.3148.7 | 54.50668 | 3.523325 | 1.511351 |  | 2.331241 | 0.019741 | 0.431683 | LOC101797856 |
| MSTRG.3187.3 | 395.0009 | -12.0627 | 3.906874 |  | -3.08755 | 0.002018 | 0.107735 | AGO2 |
| MSTRG.3268.1 | 412.9717 | -1.99366 | 0.602179 |  | -3.31074 | 0.00093 | 0.064303 | ZHX1 |
| MSTRG.3283.3 | 114.0931 | -1.30388 | 0.451929 |  | -2.88515 | 0.003912 | 0.168747 | MRPL13 |
| MSTRG.3301.4 | 70.65702 | 9.62282 | 3.024321 |  | 3.181812 | 0.001464 | 0.088296 | EXT1 |
| MSTRG.3393.1 | 177.1762 | -2.03283 | 0.447225 |  | -4.54544 | 5.48E-06 | 0.001034 | MATN2 |
| MSTRG.3495.1 | 1792.597 | -1.36829 | 0.506662 |  | -2.7006 | 0.006921 | 0.242508 | PAG1 |
| MSTRG.3833.3 | 16.15395 | -7.45103 | 3.514947 |  | -2.11981 | 0.034022 | 0.555556 | RBBP8 |
| MSTRG.3840.2 | 202.9057 | 2.702534 | 1.095139 |  | 2.467754 | 0.013596 | 0.355791 | LAMA3 |
| MSTRG.3941.1 | 4153.309 | 2.790514 | 0.923327 |  | 3.022237 | 0.002509 | 0.125541 | NR4A3 |
| MSTRG.4047.1 | 1575.476 | 1.202229 | 0.573873 |  | 2.094938 | 0.036176 | 0.5706 | IKZF1 |
| MSTRG.4078.1 | 36.91573 | -1.88489 | 0.755085 |  | -2.49626 | 0.012551 | 0.338348 | CTNND2 |
| MSTRG.4093.3 | 1194.374 | 2.746845 | 1.283301 |  | 2.140454 | 0.032318 | 0.547849 | PXDC1 |
| MSTRG.4104.2 | 145.5816 | 2.992182 | 1.07216 |  | 2.790799 | 0.005258 | 0.201121 | IRF4 |
| MSTRG.415.3 | 459.7896 | 1.08446 | 0.530177 |  | 2.045465 | 0.040809 | 0.600533 | SLC38A1 |
| MSTRG.4271.3 | 197.3705 | 1.035229 | 0.503146 |  | 2.05751 | 0.039637 | 0.594241 | LOC113841408 |
| MSTRG.4271.39 | 92.67508 | -1.37439 | 0.643671 |  | -2.13523 | 0.032742 | 0.549611 | LOC113841408 |
| MSTRG.4271.42 | 14.89936 | -7.33388 | 2.429373 |  | -3.01884 | 0.002537 | 0.126117 | LOC113841408 |
| MSTRG.4350.2 | 326.7766 | 1.426538 | 0.630525 |  | 2.26246 | 0.023669 | 0.472939 | LOC101805253 |
| MSTRG.4350.9 | 20.68318 | 2.45536 | 1.13915 |  | 2.155431 | 0.031128 | 0.539815 | LOC101805253 |
| MSTRG.4431.9 | 248.5691 | -1.33012 | 0.578592 |  | -2.29889 | 0.021511 | 0.449944 | NUP153 |
| MSTRG.4479.1 | 398.7468 | -1.04092 | 0.379963 |  | -2.73954 | 0.006153 | 0.221996 | SOX4 |
| MSTRG.4503.7 | 584.2004 | 1.464821 | 0.74473 |  | 1.966916 | 0.049193 | 0.648969 | NFATC1 |
| MSTRG.4505.3 | 16.11806 | 7.490868 | 3.509122 |  | 2.134685 | 0.032787 | 0.549611 | SLC66A2 |
| MSTRG.4562.3 | 84.79511 | 9.885878 | 2.881743 |  | 3.43052 | 0.000602 | 0.046041 | DNAJC13 |
| MSTRG.4602.2 | 23.89629 | -4.37069 | 1.782101 |  | -2.45255 | 0.014185 | 0.362343 | CMTM8 |
| MSTRG.4626.11 | 221.8278 | 1.342997 | 0.534899 |  | 2.510751 | 0.012047 | 0.330953 | SLC4A7 |
| MSTRG.4812.9 | 19.45806 | -7.71954 | 3.444406 |  | -2.24118 | 0.025014 | 0.488632 | THSD7A |
| MSTRG.4831.9 | 63.94805 | -9.43592 | 1.722284 |  | -5.47872 | 4.28E-08 | 1.57E-05 | PPP1R9A |
| MSTRG.4835.3 | 82.6506 | -8.84156 | 1.613978 |  | -5.47812 | 4.30E-08 | 1.57E-05 | COL1A2 |
| MSTRG.503.1 | 120.7306 | -4.10656 | 1.42219 |  | -2.88749 | 0.003883 | 0.167889 | NAP1L1 |
| MSTRG.503.10 | 35.95354 | 8.647851 | 1.74073 |  | 4.967946 | 6.77E-07 | 0.000174 | NAP1L1 |
| MSTRG.503.12 | 45.36866 | -8.94063 | 3.074382 |  | -2.90811 | 0.003636 | 0.16034 | NAP1L1 |
| MSTRG.503.8 | 579.4863 | 1.302854 | 0.511232 |  | 2.54846 | 0.01082 | 0.313287 | NAP1L1 |
| MSTRG.5040.2 | 333.0453 | -1.19059 | 0.456516 |  | -2.608 | 0.009107 | 0.282466 | UBE3C |
| MSTRG.5056.2 | 1113.845 | 3.978647 | 1.358385 |  | 2.928955 | 0.003401 | 0.153579 | KMT2C |
| MSTRG.5125.1 | 74.37246 | -1.24353 | 0.633937 |  | -1.96161 | 0.049808 | 0.652242 | C2H1orf35 |
| MSTRG.5204.15 | 91.23192 | -1.60388 | 0.546658 |  | -2.93398 | 0.003346 | 0.151661 | LOC101792542 |
| MSTRG.5252.3 | 1106.28 | 1.182872 | 0.441216 |  | 2.680938 | 0.007342 | 0.250037 | SSTR4 |
| MSTRG.5252.4 | 24.83037 | -8.07099 | 1.952373 |  | -4.13394 | 3.57E-05 | 0.004891 | SSTR4 |
| MSTRG.5403.4 | 20.08044 | -7.76456 | 1.8554 |  | -4.18484 | 2.85E-05 | 0.00415 | C1D |
| MSTRG.5436.15 | 710.8015 | 1.277145 | 0.397945 |  | 3.209354 | 0.00133 | 0.083196 | EHBP1 |
| MSTRG.567.6 | 156.1375 | -1.95856 | 0.984445 |  | -1.98951 | 0.046645 | 0.633729 | LOC101801431 |
| MSTRG.573.2 | 123.1313 | 2.416018 | 0.980619 |  | 2.463768 | 0.013749 | 0.357676 | LOC101799094 |
| MSTRG.5762.2 | 63.21159 | -1.23322 | 0.549886 |  | -2.24268 | 0.024917 | 0.487394 | TARBP1 |
| MSTRG.5846.5 | 389.4303 | 12.08524 | 1.265098 |  | 9.552803 | 1.26E-21 | 4.74E-18 | STRN |
| MSTRG.5862.9 | 1252.855 | 1.557561 | 0.599193 |  | 2.599431 | 0.009338 | 0.287687 | LTBP1 |
| MSTRG.5930.5 | 647.921 | 1.243142 | 0.617554 |  | 2.01301 | 0.044114 | 0.620826 | BABAM2 |
| MSTRG.609.2 | 110.8423 | -1.06333 | 0.401919 |  | -2.64563 | 0.008154 | 0.264688 | WDR91 |
| MSTRG.6126.2 | 56.09788 | -9.24692 | 2.989968 |  | -3.09265 | 0.001984 | 0.106803 | TCP1 |
| MSTRG.6184.2 | 51.05449 | 9.153945 | 3.015138 |  | 3.035996 | 0.002397 | 0.121572 | SYNE1 |
| MSTRG.6204.2 | 79.10138 | -8.77805 | 2.523081 |  | -3.4791 | 0.000503 | 0.039745 | TIAM2 |
| MSTRG.6285.13 | 43.72677 | -8.88761 | 3.064512 |  | -2.90017 | 0.00373 | 0.163305 | BCLAF1 |
| MSTRG.6285.15 | 53.90975 | -9.18952 | 3.004997 |  | -3.05808 | 0.002228 | 0.114823 | BCLAF1 |
| MSTRG.6285.3 | 82.69511 | 2.225317 | 0.814102 |  | 2.733463 | 0.006267 | 0.224835 | BCLAF1 |
| MSTRG.6435.7 | 368.7549 | 1.772867 | 0.532906 |  | 3.326789 | 0.000879 | 0.061277 | FYN |
| MSTRG.6609.4 | 174.94 | 1.881205 | 0.834936 |  | 2.253114 | 0.024252 | 0.478724 | LOC101805198 |
| MSTRG.6833.10 | 212.1306 | -11.1658 | 3.906969 |  | -2.85791 | 0.004264 | 0.175848 | PUM2 |
| MSTRG.6933.5 | 132.1112 | 1.236587 | 0.434699 |  | 2.844694 | 0.004445 | 0.180559 | MSRA |
| MSTRG.6939.25 | 22.23108 | 7.954174 | 1.986938 |  | 4.003232 | 6.25E-05 | 0.007868 | HMBOX1 |
| MSTRG.6960.2 | 378.9952 | -2.89567 | 1.154114 |  | -2.50899 | 0.012108 | 0.331853 | SCARA5 |
| MSTRG.7099.4 | 635.5774 | 1.097931 | 0.254263 |  | 4.318088 | 1.57E-05 | 0.002485 | AP1AR |
| MSTRG.7118.6 | 81.17057 | 4.897395 | 1.877403 |  | 2.608601 | 0.009091 | 0.282466 | LEF1 |
| MSTRG.7142.3 | 92.71451 | 10.01472 | 2.843705 |  | 3.521716 | 0.000429 | 0.034924 | PPA2 |
| MSTRG.7228.1 | 509.5315 | 1.066188 | 0.535315 |  | 1.991702 | 0.046404 | 0.631857 | GPRIN3 |
| MSTRG.7276.3 | 279.2999 | -3.28709 | 1.045955 |  | -3.14267 | 0.001674 | 0.096871 | YTHDC1 |
| MSTRG.7285.2 | 309.3344 | 1.906184 | 0.660996 |  | 2.883806 | 0.003929 | 0.169078 | SLC4A4 |
| MSTRG.7304.3 | 899.5319 | -2.54541 | 0.750469 |  | -3.39176 | 0.000694 | 0.051399 | CCNI |
| MSTRG.7326.1 | 1277.587 | -1.06411 | 0.325951 |  | -3.26464 | 0.001096 | 0.072176 | PURG |
| MSTRG.7343.3 | 296.1716 | -1.64956 | 0.769285 |  | -2.14428 | 0.032011 | 0.546665 | CNOT7 |
| MSTRG.7343.5 | 43.9209 | -8.89392 | 3.168259 |  | -2.80719 | 0.004998 | 0.194315 | CNOT7 |
| MSTRG.7370.2 | 17.49366 | -7.56571 | 3.564849 |  | -2.12231 | 0.033812 | 0.554784 | SORBS2 |
| MSTRG.7439.3 | 50.33895 | 2.413127 | 0.485242 |  | 4.973041 | 6.59E-07 | 0.000171 | HMGB2 |
| MSTRG.75.2 | 120.4787 | -1.57092 | 0.787962 |  | -1.99365 | 0.04619 | 0.63158 | EXOC4 |
| MSTRG.7517.11 | 235.9232 | -1.72582 | 0.849055 |  | -2.03263 | 0.042089 | 0.608722 | ARFIP1 |
| MSTRG.7563.4 | 706.0706 | 1.833984 | 0.442026 |  | 4.149045 | 3.34E-05 | 0.004657 | LOC101794667 |
| MSTRG.7570.13 | 54.2503 | -9.19863 | 3.002682 |  | -3.06347 | 0.002188 | 0.113085 | GAB1 |
| MSTRG.760.13 | 258.2714 | -11.4499 | 1.638156 |  | -6.98953 | 2.76E-12 | 2.59E-09 | TNRC6B |
| MSTRG.760.26 | 1508.813 | -1.33037 | 0.379629 |  | -3.50439 | 0.000458 | 0.036932 | TNRC6B |
| MSTRG.760.5 | 415.011 | 5.84555 | 1.730044 |  | 3.378844 | 0.000728 | 0.052936 | TNRC6B |
| MSTRG.7652.2 | 167.161 | 6.59286 | 1.43018 |  | 4.609812 | 4.03E-06 | 0.000784 | BBS7 |
| MSTRG.7690.5 | 75.6183 | -9.67768 | 1.932047 |  | -5.00903 | 5.47E-07 | 0.000145 | SEC31A |
| MSTRG.7695.4 | 2150.604 | 1.752551 | 0.640375 |  | 2.736757 | 0.006205 | 0.223022 | ARHGAP24 |
| MSTRG.774.9 | 398.1762 | 1.020142 | 0.402753 |  | 2.53292 | 0.011312 | 0.319391 | MGAT3 |
| MSTRG.7788.4 | 459.1236 | 12.32278 | 1.857306 |  | 6.63476 | 3.25E-11 | 2.39E-08 | FRYL |
| MSTRG.787.3 | 58.59529 | 9.3529 | 2.967222 |  | 3.152073 | 0.001621 | 0.09461 | CBX7 |
| MSTRG.787.7 | 89.56366 | 3.097393 | 1.502468 |  | 2.061537 | 0.039252 | 0.592771 | CBX7 |
| MSTRG.787.8 | 69.49722 | 2.898842 | 1.041986 |  | 2.782036 | 0.005402 | 0.204084 | CBX7 |
| MSTRG.794.3 | 17.65156 | 7.62209 | 3.625197 |  | 2.102532 | 0.035507 | 0.565716 | JOSD1 |
| MSTRG.7978.11 | 20.45391 | -7.79146 | 3.385217 |  | -2.30161 | 0.021357 | 0.448759 | SH3TC1 |
| MSTRG.8022.1 | 480.7494 | -1.15445 | 0.455497 |  | -2.53449 | 0.011261 | 0.319391 | FGFR3 |
| MSTRG.8034.1 | 129.3693 | 1.053392 | 0.357699 |  | 2.944913 | 0.00323 | 0.148996 | UVSSA |
| MSTRG.8177.4 | 270.606 | -1.30625 | 0.662939 |  | -1.97039 | 0.048794 | 0.646539 | MDGA2 |
| MSTRG.8195.2 | 34.6074 | -5.35146 | 2.028648 |  | -2.63794 | 0.008341 | 0.267519 | L2HGDH |
| MSTRG.8209.2 | 90.65758 | -1.05419 | 0.506684 |  | -2.08057 | 0.037474 | 0.581878 | FRMD6 |
| MSTRG.8214.1 | 2284.527 | 1.036801 | 0.502679 |  | 2.062549 | 0.039155 | 0.592463 | NID2 |
| MSTRG.8227.6 | 19.8438 | 7.790339 | 3.478118 |  | 2.239814 | 0.025103 | 0.489345 | DDHD1 |
| MSTRG.8227.7 | 212.1328 | 1.087347 | 0.543976 |  | 1.998888 | 0.04562 | 0.628223 | DDHD1 |
| MSTRG.8254.2 | 714.62 | -1.02697 | 0.293398 |  | -3.50028 | 0.000465 | 0.037266 | NAA30 |
| MSTRG.8332.2 | 75.92596 | -9.68363 | 2.91411 |  | -3.32301 | 0.000891 | 0.061767 | CEP170B |
| MSTRG.8343.3 | 608.3304 | 7.680542 | 1.414038 |  | 5.43164 | 5.58E-08 | 1.98E-05 | LOC101799876 |
| MSTRG.8343.4 | 549.5749 | -2.68709 | 0.375906 |  | -7.14832 | 8.78E-13 | 9.42E-10 | LOC101799876 |
| MSTRG.8402.2 | 278.8103 | 3.293606 | 0.58818 |  | 5.599655 | 2.15E-08 | 8.48E-06 | PPP2R5C |
| MSTRG.8411.1 | 93.27516 | 10.0234 | 1.91299 |  | 5.239652 | 1.61E-07 | 5.15E-05 | EVL |
| MSTRG.8423.1 | 927.0609 | 1.305493 | 0.631134 |  | 2.068488 | 0.038594 | 0.590158 | BCL11B |
| MSTRG.8460.1 | 767.485 | -13.0211 | 1.208559 |  | -10.7741 | 4.56E-27 | 8.56E-23 | RIN3 |
| MSTRG.8471.3 | 67.18847 | -9.50725 | 1.633031 |  | -5.82184 | 5.82E-09 | 2.70E-06 | CCDC88C |
| MSTRG.8471.4 | 110.6478 | 10.26984 | 2.802144 |  | 3.664994 | 0.000247 | 0.022901 | CCDC88C |
| MSTRG.855.7 | 126.6888 | -1.20426 | 0.482293 |  | -2.49695 | 0.012527 | 0.338055 | RBFOX2 |
| MSTRG.8572.1 | 75.72784 | 2.368433 | 1.01489 |  | 2.333684 | 0.019612 | 0.431007 | TMED8 |
| MSTRG.8614.8 | 125.3704 | -10.4071 | 2.889624 |  | -3.60154 | 0.000316 | 0.027865 | YLPM1 |
| MSTRG.8617.1 | 62.52188 | -9.40369 | 1.429747 |  | -6.57717 | 4.79E-11 | 3.16E-08 | AREL1 |
| MSTRG.8693.3 | 986.2041 | 1.288977 | 0.594736 |  | 2.167309 | 0.030211 | 0.532994 | CHKA |
| MSTRG.8693.4 | 242.2462 | 1.309609 | 0.561308 |  | 2.333136 | 0.019641 | 0.431187 | CHKA |
| MSTRG.8698.2 | 101.702 | 10.14827 | 2.985091 |  | 3.399651 | 0.000675 | 0.050336 | MUC2 |
| MSTRG.8783.1 | 19.44666 | -2.30592 | 0.910242 |  | -2.5333 | 0.011299 | 0.319391 | SSH3 |
| MSTRG.8832.16 | 68.22789 | -9.52941 | 2.920524 |  | -3.26291 | 0.001103 | 0.072343 | DAGLA |
| MSTRG.8942.9 | 244.0206 | 1.46619 | 0.575969 |  | 2.545605 | 0.010909 | 0.31409 | ATG13 |
| MSTRG.8949.9 | 406.5286 | -1.0492 | 0.452559 |  | -2.31837 | 0.020429 | 0.439085 | PHF21A |
| MSTRG.8955.1 | 136.479 | -1.0814 | 0.34728 |  | -3.11392 | 0.001846 | 0.101882 | SLC35C1 |
| MSTRG.903.10 | 275.6388 | -1.35724 | 0.64137 |  | -2.11615 | 0.034332 | 0.558096 | TXNRD1 |
| MSTRG.903.5 | 65.1318 | -9.4626 | 1.369514 |  | -6.90945 | 4.87E-12 | 4.25E-09 | TXNRD1 |
| MSTRG.9099.3 | 1090.975 | 1.653596 | 0.343544 |  | 4.813342 | 1.48E-06 | 0.00033 | MPP5 |
| MSTRG.9170.1 | 2106.296 | -1.19264 | 0.409765 |  | -2.91055 | 0.003608 | 0.159654 | HECTD1 |
| MSTRG.9183.1 | 198.2747 | -1.35577 | 0.553165 |  | -2.45093 | 0.014249 | 0.362989 | SNX6 |
| MSTRG.9310.43 | 306.6831 | 1.397611 | 0.392915 |  | 3.557032 | 0.000375 | 0.031771 | MADD |
| MSTRG.94.1 | 357.5101 | 1.398562 | 0.678572 |  | 2.061037 | 0.0393 | 0.592771 | PRKCQ |
| MSTRG.9510.4 | 707.3537 | 1.09003 | 0.471578 |  | 2.311451 | 0.020808 | 0.443395 | SWAP70 |
| MSTRG.9526.27 | 19.37585 | -7.71338 | 3.410474 |  | -2.26167 | 0.023718 | 0.473529 | IRAG1 |
| MSTRG.9526.28 | 88.70712 | -1.16415 | 0.590728 |  | -1.9707 | 0.048758 | 0.646539 | IRAG1 |
| MSTRG.9550.29 | 22.39988 | 7.965175 | 1.884079 |  | 4.227622 | 2.36E-05 | 0.003545 | MICAL2 |
| MSTRG.956.7 | 70.95043 | 3.197196 | 1.544277 |  | 2.070351 | 0.038419 | 0.588926 | KIAA1549 |
| MSTRG.9695.6 | 875.0656 | -2.30586 | 0.50138 |  | -4.59903 | 4.24E-06 | 0.000817 | TET1 |
| MSTRG.9714.12 | 44.92321 | -8.92657 | 3.231739 |  | -2.76216 | 0.005742 | 0.213515 | JMJD1C |
| MSTRG.9786.3 | 20.0155 | -7.76016 | 3.435365 |  | -2.2589 | 0.023889 | 0.474815 | RASGEF1A |
| MSTRG.9786.6 | 222.6733 | -1.27759 | 0.535204 |  | -2.38711 | 0.016981 | 0.398586 | RASGEF1A |
| MSTRG.9889.1 | 334.2003 | 1.161943 | 0.482274 |  | 2.409302 | 0.015983 | 0.385205 | ALOX5 |
| MSTRG.9936.1 | 102.1839 | 10.1552 | 1.8642 |  | 5.447482 | 5.11E-08 | 1.83E-05 | PCGF5 |
| rna-NM_001310414.2 | 1307.582 | -1.05371 | 0.428018 |  | -2.46183 | 0.013823 | 0.358006 | LSM14A |
| rna-NM_001310420.1 | 75.02376 | 1.763577 | 0.856147 |  | 2.0599 | 0.039408 | 0.593415 | IL8 |
| rna-NM_001310789.1 | 38.7201 | -8.71214 | 1.774894 |  | -4.90854 | 9.18E-07 | 0.000221 | BMP4 |
| rna-NM_001310813.1 | 171.5865 | 2.474591 | 0.892706 |  | 2.772012 | 0.005571 | 0.20843 | LOC101802909 |
| rna-NM_001310817.1 | 102.8127 | 2.013703 | 0.685905 |  | 2.935835 | 0.003327 | 0.151306 | CD8A |
| rna-NM_001310822.2 | 180.315 | 1.762195 | 0.722847 |  | 2.437853 | 0.014775 | 0.369315 | LOC101805253 |
| rna-XM_005008806.5 | 122.5545 | 1.430385 | 0.711607 |  | 2.010078 | 0.044423 | 0.622469 | LOC101791564 |
| rna-XM_005008890.5 | 418.2451 | 2.207527 | 0.988792 |  | 2.232551 | 0.025579 | 0.49327 | ZC3H12D |
| rna-XM_005009090.5 | 7.324644 | 6.351987 | 2.859707 |  | 2.221202 | 0.026337 | 0.500125 | STOX1 |
| rna-XM_005009316.5 | 57.92571 | 4.354042 | 2.187117 |  | 1.990768 | 0.046506 | 0.632761 | FBXO38 |
| rna-XM_005009586.5 | 74.09124 | 2.236486 | 1.093084 |  | 2.046033 | 0.040753 | 0.600533 | THEMIS |
| rna-XM_005010136.5 | 604.323 | -1.51713 | 0.656172 |  | -2.31209 | 0.020773 | 0.443248 | GFRA4 |
| rna-XM_005010421.5 | 346.3516 | 2.489905 | 0.829018 |  | 3.003439 | 0.002669 | 0.130139 | LOC101796210 |
| rna-XM_005010500.5 | 42.96827 | 3.191367 | 1.305067 |  | 2.445367 | 0.01447 | 0.366337 | LOC101794528 |
| rna-XM_005010503.5 | 851.4267 | 1.15939 | 0.471337 |  | 2.459791 | 0.013902 | 0.358286 | P2RY8 |
| rna-XM_005010655.5 | 697.9184 | 2.083088 | 0.80278 |  | 2.594842 | 0.009463 | 0.289182 | LOC101791569 |
| rna-XM_005011275.4 | 132.7049 | -3.06195 | 1.33346 |  | -2.29624 | 0.021662 | 0.452096 | EZH2 |
| rna-XM_005011289.5 | 328.6041 | 2.258407 | 0.896697 |  | 2.518584 | 0.011783 | 0.326791 | EHF |
| rna-XM_005011750.5 | 1584.293 | 1.146645 | 0.524802 |  | 2.184908 | 0.028896 | 0.525852 | RASSF5 |
| rna-XM_005011965.5 | 140.0321 | 1.6823 | 0.734885 |  | 2.2892 | 0.022068 | 0.457509 | LOC101791390 |
| rna-XM_005012101.5 | 638.4573 | 1.040976 | 0.478274 |  | 2.176527 | 0.029516 | 0.528936 | SEC23B |
| rna-XM_005012231.5 | 129.4726 | 1.500216 | 0.627241 |  | 2.39177 | 0.016767 | 0.396218 | SATB1 |
| rna-XM_005012247.4 | 226.1452 | 1.345972 | 0.603233 |  | 2.231264 | 0.025664 | 0.494367 | LOC101795049 |
| rna-XM_005012419.5 | 31.804 | 3.695933 | 1.387905 |  | 2.662958 | 0.007746 | 0.255861 | AICDA |
| rna-XM_005012470.5 | 77.00917 | -4.12103 | 2.0252 |  | -2.03487 | 0.041863 | 0.607004 | AGPAT3 |
| rna-XM_005012660.5 | 104.2912 | 1.911633 | 0.802273 |  | 2.382771 | 0.017183 | 0.40049 | LOC101804544 |
| rna-XM_005012662.5 | 19.94992 | 7.797729 | 2.032612 |  | 3.83631 | 0.000125 | 0.013558 | LOC101804866 |
| rna-XM_005012766.5 | 12.81124 | 7.158581 | 2.174869 |  | 3.291499 | 0.000997 | 0.067017 | RASGEF1B |
| rna-XM_005012779.5 | 206.7534 | 3.365311 | 0.988039 |  | 3.40605 | 0.000659 | 0.049367 | SPP1 |
| rna-XM_005013214.5 | 157.8532 | 1.457637 | 0.413856 |  | 3.522088 | 0.000428 | 0.034924 | NTMT1 |
| rna-XM_005013893.5 | 83.58604 | 1.878738 | 0.510313 |  | 3.681538 | 0.000232 | 0.021803 | CEP128 |
| rna-XM_005014057.4 | 40.45789 | -3.60346 | 1.539213 |  | -2.3411 | 0.019227 | 0.426409 | LOC101796420 |
| rna-XM_005014621.5 | 106.4116 | 1.643509 | 0.60097 |  | 2.734759 | 0.006243 | 0.224166 | CNTN2 |
| rna-XM_005014642.5 | 550.0533 | -3.35498 | 1.439607 |  | -2.33048 | 0.019781 | 0.432308 | RNH1 |
| rna-XM_005015203.5 | 12.41174 | -7.07099 | 2.048773 |  | -3.45133 | 0.000558 | 0.04361 | CAPN6 |
| rna-XM_005015719.5 | 128.8164 | 1.522441 | 0.505251 |  | 3.013238 | 0.002585 | 0.127622 | SAMD8 |
| rna-XM_005016030.5 | 95.12453 | -10.009 | 1.437157 |  | -6.96444 | 3.30E-12 | 3.02E-09 | SPAG9 |
| rna-XM_005016051.5 | 20.09006 | 3.505179 | 1.291111 |  | 2.714854 | 0.006631 | 0.235071 | ARC |
| rna-XM_005016600.5 | 47.4125 | 1.82679 | 0.751387 |  | 2.431223 | 0.015048 | 0.371635 | GDPD2 |
| rna-XM_005016831.5 | 215.8594 | 2.169502 | 0.951083 |  | 2.281085 | 0.022543 | 0.463469 | CTLA4 |
| rna-XM_005016946.5 | 321.2584 | 1.479701 | 0.647695 |  | 2.284566 | 0.022338 | 0.461279 | NEDD9 |
| rna-XM_005016977.4 | 84.07096 | -1.53593 | 0.773443 |  | -1.98583 | 0.047052 | 0.636683 | LDB2 |
| rna-XM_005016983.5 | 22.87198 | -6.97949 | 2.73768 |  | -2.54942 | 0.01079 | 0.3132 | LDB2 |
| rna-XM_005017637.4 | 921.8175 | 1.351536 | 0.631113 |  | 2.141514 | 0.032233 | 0.547298 | CD80 |
| rna-XM_005018148.5 | 183.2647 | 1.149005 | 0.563519 |  | 2.038983 | 0.041452 | 0.604645 | SPRED2 |
| rna-XM_005018425.5 | 519.77 | -1.38574 | 0.520443 |  | -2.66261 | 0.007754 | 0.2559 | GJC1 |
| rna-XM_005018484.5 | 20.17996 | 7.814834 | 3.434679 |  | 2.275274 | 0.02289 | 0.465797 | TMC5 |
| rna-XM_005018715.5 | 77.79051 | 1.224734 | 0.545363 |  | 2.245725 | 0.024722 | 0.485696 | LOC101800311 |
| rna-XM_005019063.5 | 64.07467 | -1.79489 | 0.579045 |  | -3.09973 | 0.001937 | 0.104732 | CHDH |
| rna-XM_005019168.5 | 736.118 | 1.213691 | 0.527685 |  | 2.30003 | 0.021447 | 0.449347 | PLCB2 |
| rna-XM_005019283.5 | 227.8691 | 1.28994 | 0.450195 |  | 2.865292 | 0.004166 | 0.174149 | SOAT1 |
| rna-XM_005019381.5 | 46.25301 | -8.9685 | 3.118283 |  | -2.8761 | 0.004026 | 0.171476 | CEP63 |
| rna-XM_005019660.5 | 7.629555 | 3.054249 | 1.431246 |  | 2.133979 | 0.032844 | 0.549973 | TMEM45B |
| rna-XM_005019759.5 | 85.09931 | 1.371203 | 0.609308 |  | 2.250428 | 0.024422 | 0.481317 | TRPM5 |
| rna-XM_005019781.3 | 398.8224 | 1.295755 | 0.630081 |  | 2.056491 | 0.039735 | 0.59454 | PHLDA2 |
| rna-XM_005019899.5 | 167.7138 | 10.86996 | 2.70567 |  | 4.017474 | 5.88E-05 | 0.007483 | ITPR1 |
| rna-XM_005020113.5 | 752.8166 | 1.392706 | 0.656952 |  | 2.11995 | 0.03401 | 0.555556 | CARD11 |
| rna-XM_005020118.5 | 17.40202 | -7.55806 | 1.874833 |  | -4.03132 | 5.55E-05 | 0.007103 | LOC101799374 |
| rna-XM_005020288.5 | 5.567852 | -5.91401 | 2.669109 |  | -2.21573 | 0.02671 | 0.503417 | MAP3K19 |
| rna-XM_005020529.5 | 87.18582 | 2.550337 | 1.265363 |  | 2.015498 | 0.043852 | 0.619565 | GPNMB |
| rna-XM_005021005.5 | 167.6308 | 1.106544 | 0.535778 |  | 2.065302 | 0.038894 | 0.591136 | OMG |
| rna-XM_005021008.5 | 690.1198 | 2.000067 | 0.702716 |  | 2.846196 | 0.004424 | 0.180466 | EVI2A |
| rna-XM_005021532.5 | 195.069 | 2.247387 | 1.10852 |  | 2.027375 | 0.042624 | 0.61212 | SPIC |
| rna-XM_005021533.5 | 90.91983 | 2.719461 | 1.111269 |  | 2.447166 | 0.014398 | 0.365562 | SPIC |
| rna-XM_005021706.3 | 129.2639 | -1.48278 | 0.576202 |  | -2.57338 | 0.010071 | 0.301633 | LMNA |
| rna-XM_005021747.5 | 410.7411 | 2.560044 | 0.893644 |  | 2.864724 | 0.004174 | 0.174149 | CD79B |
| rna-XM_005022052.5 | 165.2584 | 1.034404 | 0.471146 |  | 2.195504 | 0.028127 | 0.516561 | MRPS5 |
| rna-XM_005022192.5 | 265.1139 | 1.43358 | 0.60758 |  | 2.359491 | 0.0183 | 0.415431 | TMEM273 |
| rna-XM_005022554.5 | 97.2109 | -3.00221 | 1.181556 |  | -2.54089 | 0.011057 | 0.316197 | PAH |
| rna-XM_005022627.5 | 44.52827 | -8.91366 | 3.080418 |  | -2.89365 | 0.003808 | 0.165575 | ARHGEF9 |
| rna-XM_005022738.5 | 4.94813 | 5.786234 | 2.671986 |  | 2.165518 | 0.030348 | NA | IKZF1 |
| rna-XM_005022739.5 | 97.94515 | 6.62502 | 2.007357 |  | 3.30037 | 0.000966 | 0.065998 | IKZF1 |
| rna-XM_005022766.5 | 2151.109 | 1.083493 | 0.335962 |  | 3.225049 | 0.00126 | 0.079567 | CPT1A |
| rna-XM_005023093.5 | 796.6168 | 1.207192 | 0.481649 |  | 2.506374 | 0.012198 | 0.332885 | AKAP10 |
| rna-XM_005023107.5 | 308.135 | 1.508863 | 0.640722 |  | 2.35494 | 0.018526 | 0.418229 | P2RX5 |
| rna-XM_005023235.5 | 45.38567 | -2.88713 | 1.368265 |  | -2.11006 | 0.034853 | 0.560548 | LOC101805135 |
| rna-XM_005023249.5 | 32.77366 | 3.82824 | 1.886964 |  | 2.028782 | 0.04248 | 0.611227 | SNTN |
| rna-XM_005023561.5 | 34.22505 | -8.53426 | 1.685255 |  | -5.06408 | 4.10E-07 | 0.000114 | ADORA2A |
| rna-XM_005023697.5 | 983.0021 | 2.063823 | 0.832567 |  | 2.478869 | 0.01318 | 0.348541 | CD83 |
| rna-XM_005023811.5 | 59.60365 | 1.049541 | 0.522466 |  | 2.008824 | 0.044556 | 0.622935 | GPC3 |
| rna-XM_005024104.5 | 326.9183 | 2.067447 | 0.808953 |  | 2.555707 | 0.010597 | 0.309729 | CD96 |
| rna-XM_005024804.5 | 236.9595 | 1.686879 | 0.799836 |  | 2.109031 | 0.034942 | 0.561299 | RUBCNL |
| rna-XM_005024958.5 | 7.389433 | 3.789168 | 1.883023 |  | 2.012279 | 0.044191 | 0.621068 | INSYN2B |
| rna-XM_005024959.5 | 793.9652 | 1.454347 | 0.475155 |  | 3.060784 | 0.002208 | 0.113947 | DOCK2 |
| rna-XM_005025095.5 | 69.47783 | 1.393575 | 0.592482 |  | 2.352095 | 0.018668 | 0.420248 | SLC41A3 |
| rna-XM_005025107.5 | 12.0233 | -7.02489 | 1.989826 |  | -3.53041 | 0.000415 | 0.03418 | UROC1 |
| rna-XM_005025167.5 | 98.76812 | 1.069817 | 0.529857 |  | 2.019068 | 0.04348 | 0.618118 | C1D |
| rna-XM_005025440.5 | 17.95431 | -6.629 | 2.265894 |  | -2.92556 | 0.003438 | 0.154894 | LIPC |
| rna-XM_005025990.5 | 1351.793 | 4.060082 | 1.904368 |  | 2.131984 | 0.033008 | 0.551184 | SLC6A6 |
| rna-XM_005026097.5 | 22.63459 | -5.53729 | 1.952062 |  | -2.83663 | 0.004559 | 0.183763 | CMBL |
| rna-XM_005026212.5 | 13.63346 | 7.249394 | 3.603352 |  | 2.011847 | 0.044236 | 0.6213 | LOC101792067 |
| rna-XM_005026213.5 | 16.6704 | 7.539451 | 3.504022 |  | 2.151656 | 0.031424 | 0.541913 | LOC101792067 |
| rna-XM_005026421.4 | 33.48688 | 1.922394 | 0.794378 |  | 2.419999 | 0.015521 | 0.377942 | MOGAT1 |
| rna-XM_005026612.5 | 460.1428 | 2.944659 | 0.957462 |  | 3.075483 | 0.002102 | 0.110298 | CXCR5 |
| rna-XM_005026729.5 | 81.07169 | 2.679664 | 1.054692 |  | 2.540707 | 0.011063 | 0.316197 | PDCD1 |
| rna-XM_005026830.5 | 1003.4 | 1.137787 | 0.509622 |  | 2.232608 | 0.025575 | 0.49327 | BAK1 |
| rna-XM_005026847.5 | 183.3536 | 2.869685 | 1.021136 |  | 2.810288 | 0.00495 | 0.193477 | LOC101792733 |
| rna-XM_005027522.5 | 58.71548 | 2.034302 | 0.956856 |  | 2.126027 | 0.033501 | 0.554394 | TOR4A |
| rna-XM_005027895.5 | 97.50989 | 1.820339 | 0.813535 |  | 2.237566 | 0.025249 | 0.490669 | ARHGEF38 |
| rna-XM_005027924.5 | 39.24914 | -8.73169 | 3.138376 |  | -2.78223 | 0.005399 | 0.204084 | IREB2 |
| rna-XM_005028449.5 | 12.15946 | -7.0412 | 2.092914 |  | -3.36431 | 0.000767 | 0.055163 | ARMC1 |
| rna-XM_005028669.5 | 43.03583 | 8.907427 | 3.072971 |  | 2.898637 | 0.003748 | 0.163534 | RASGEF1C |
| rna-XM_005028921.5 | 1088.776 | 1.163616 | 0.270347 |  | 4.304162 | 1.68E-05 | 0.002621 | PTPRS |
| rna-XM_005029352.5 | 1090.021 | 1.038612 | 0.213637 |  | 4.861583 | 1.16E-06 | 0.00027 | SBNO2 |
| rna-XM_005029577.5 | 12.73741 | 6.170694 | 2.890794 |  | 2.134602 | 0.032794 | 0.549611 | LOC101790444 |
| rna-XM_005029737.5 | 114.4962 | -1.61842 | 0.815236 |  | -1.98521 | 0.047121 | 0.636734 | SYTL2 |
| rna-XM_005030137.5 | 55.17532 | -9.22298 | 3.000717 |  | -3.07359 | 0.002115 | 0.110666 | FYN |
| rna-XM_005030336.5 | 2155.9 | 2.048001 | 0.778829 |  | 2.629589 | 0.008549 | 0.27186 | CCR7 |
| rna-XM_005030343.5 | 8.017135 | 3.927641 | 1.739776 |  | 2.257555 | 0.023973 | 0.475225 | KRT20 |
| rna-XM_005030411.5 | 25.27801 | 2.055953 | 0.932893 |  | 2.203847 | 0.027535 | 0.511106 | ANKRD66 |
| rna-XM_005030452.5 | 11.28953 | 6.977222 | 2.038758 |  | 3.42229 | 0.000621 | 0.047141 | PDE9A |
| rna-XM_005030599.5 | 218.3564 | 1.887908 | 0.738696 |  | 2.55573 | 0.010597 | 0.309729 | BLK |
| rna-XM_005030842.5 | 14.86576 | 7.373088 | 2.17834 |  | 3.384728 | 0.000712 | 0.052117 | CHRNA9 |
| rna-XM_005031313.4 | 9210.483 | 2.280165 | 0.828348 |  | 2.752667 | 0.005911 | 0.217139 | JCHAIN |
| rna-XM_013091718.4 | 362.8372 | -1.18405 | 0.427722 |  | -2.76828 | 0.005635 | 0.210206 | GNB1 |
| rna-XM_013091805.4 | 20.63602 | 7.847161 | 3.555693 |  | 2.206929 | 0.027319 | 0.509013 | FOXP1 |
| rna-XM_013091819.4 | 36.78745 | 3.778891 | 1.468635 |  | 2.573064 | 0.01008 | 0.301633 | FRMD4B |
| rna-XM_013091829.3 | 44.22744 | 8.946727 | 3.161237 |  | 2.830135 | 0.004653 | 0.185545 | LOC101797576 |
| rna-XM_013092027.4 | 39.95647 | 1.88987 | 0.947688 |  | 1.99419 | 0.046131 | 0.631319 | LOC101797884 |
| rna-XM_013092146.4 | 154.9965 | 1.544019 | 0.584919 |  | 2.639715 | 0.008298 | 0.267378 | CRACR2A |
| rna-XM_013092566.4 | 63.56927 | -1.47692 | 0.65157 |  | -2.26671 | 0.023408 | 0.470978 | TMCC1 |
| rna-XM_013094496.4 | 14.7603 | -7.3208 | 3.578502 |  | -2.04577 | 0.040779 | 0.600533 | CYGB |
| rna-XM_013094684.4 | 15.67618 | 7.450062 | 1.952835 |  | 3.814999 | 0.000136 | 0.014477 | PLEKHS1 |
| rna-XM_013095136.4 | 158.3426 | -1.14337 | 0.52725 |  | -2.16855 | 0.030117 | 0.53243 | TEX30 |
| rna-XM_013095145.4 | 23.84306 | -8.01267 | 3.3055 |  | -2.42404 | 0.015349 | 0.375465 | BIVM |
| rna-XM_013095349.4 | 4.016161 | 5.485841 | 2.76211 |  | 1.986105 | 0.047022 | NA | LOC101802027 |
| rna-XM_013095706.4 | 50.1286 | 9.127479 | 3.039117 |  | 3.003332 | 0.00267 | 0.130139 | HIVEP3 |
| rna-XM_013095883.4 | 26.93318 | -8.18854 | 3.362014 |  | -2.43561 | 0.014867 | 0.369946 | GP1BA |
| rna-XM_013095888.4 | 30.21452 | 8.397059 | 3.214485 |  | 2.612257 | 0.008995 | 0.280569 | GP1BA |
| rna-XM_013096106.4 | 43.45596 | 3.983917 | 1.339882 |  | 2.973335 | 0.002946 | 0.139398 | LOC101800424 |
| rna-XM_013096745.4 | 53.87218 | 1.532347 | 0.779984 |  | 1.964589 | 0.049462 | 0.650563 | LOC101800940 |
| rna-XM_013096779.4 | 9.193702 | 6.681688 | 2.338563 |  | 2.857177 | 0.004274 | 0.176062 | GPR20 |
| rna-XM_013096828.4 | 2439.526 | 1.170175 | 0.492171 |  | 2.377577 | 0.017427 | 0.403418 | PTPN6 |
| rna-XM_013096857.4 | 5.486699 | 5.936209 | 2.891641 |  | 2.052886 | 0.040084 | 0.595934 | THEMIS |
| rna-XM_013097677.4 | 16.39424 | 7.51486 | 1.853674 |  | 4.054035 | 5.03E-05 | 0.006537 | HTR1F |
| rna-XM_013099244.4 | 246.3924 | 1.501396 | 0.729322 |  | 2.058618 | 0.039531 | 0.593752 | FUT8 |
| rna-XM_013099544.4 | 27.83578 | -8.23594 | 3.243747 |  | -2.53902 | 0.011116 | 0.316975 | PKNOX1 |
| rna-XM_013099608.4 | 25.84676 | 8.171387 | 1.812552 |  | 4.508223 | 6.54E-06 | 0.001179 | CDON |
| rna-XM_013099808.4 | 367.9752 | 1.406548 | 0.675849 |  | 2.081156 | 0.03742 | 0.581878 | OSBPL5 |
| rna-XM_013100598.4 | 15.33381 | -7.37569 | 3.722725 |  | -1.98126 | 0.047562 | 0.638979 | ERCC8 |
| rna-XM_013100616.4 | 196.2391 | -4.21517 | 1.916894 |  | -2.19896 | 0.027881 | 0.514119 | HEATR5A |
| rna-XM_013100630.4 | 42.88654 | 8.901994 | 1.825885 |  | 4.87544 | 1.09E-06 | 0.000255 | LOC101800121 |
| rna-XM_013101320.4 | 105.7985 | -1.15299 | 0.534382 |  | -2.15761 | 0.030958 | 0.53882 | SEC23A |
| rna-XM_013101456.4 | 1166.372 | 1.921465 | 0.409868 |  | 4.688011 | 2.76E-06 | 0.000568 | SUSD6 |
| rna-XM_013102263.4 | 175.8888 | 1.905611 | 0.735268 |  | 2.591722 | 0.00955 | 0.290399 | TNFSF8 |
| rna-XM_013102509.4 | 92.14631 | 1.33605 | 0.634909 |  | 2.104317 | 0.035351 | 0.564726 | BRD9 |
| rna-XM_013102931.4 | 26.75993 | -6.34465 | 1.512354 |  | -4.19521 | 2.73E-05 | 0.003996 | ABCC9 |
| rna-XM_013102932.4 | 111.8966 | 2.47089 | 0.517405 |  | 4.775544 | 1.79E-06 | 0.000387 | ABCC9 |
| rna-XM_013103115.4 | 29.66011 | 8.370111 | 1.69611 |  | 4.934886 | 8.02E-07 | 0.000199 | POU2F3 |
| rna-XM_013103583.4 | 469.2273 | 1.100449 | 0.364191 |  | 3.021624 | 0.002514 | 0.125591 | PABIR2 |
| rna-XM_013103832.4 | 132.4833 | 1.607427 | 0.753605 |  | 2.132984 | 0.032926 | 0.550847 | LOC101802873 |
| rna-XM_013103963.4 | 942.4314 | -1.18643 | 0.451044 |  | -2.63042 | 0.008528 | 0.271657 | FADS2 |
| rna-XM_013104765.4 | 38.34097 | 1.661967 | 0.806956 |  | 2.059551 | 0.039441 | 0.593678 | MARCHF8 |
| rna-XM_013105349.4 | 1537.837 | 2.71686 | 1.346931 |  | 2.017075 | 0.043688 | 0.618371 | NFAT5 |
| rna-XM_013105398.4 | 27.33232 | 8.252375 | 3.273109 |  | 2.521265 | 0.011693 | 0.324958 | LOC101796210 |
| rna-XM_013105503.4 | 23.59663 | 8.040323 | 3.37543 |  | 2.382015 | 0.017218 | 0.400579 | SETD5 |
| rna-XM_013105861.4 | 215.7036 | 1.596175 | 0.615257 |  | 2.594323 | 0.009478 | 0.289384 | ZFHX4 |
| rna-XM_013106692.4 | 27.31805 | 2.139223 | 0.995154 |  | 2.149639 | 0.031584 | 0.543324 | DLEC1 |
| rna-XM_013106699.4 | 23.17065 | -7.97124 | 1.676991 |  | -4.7533 | 2.00E-06 | 0.000427 | TMEM71 |
| rna-XM_013106709.4 | 44.64416 | 4.057072 | 1.918081 |  | 2.115172 | 0.034415 | 0.558096 | TMEM71 |
| rna-XM_013106932.4 | 474.8513 | 1.1523 | 0.3566 |  | 3.231353 | 0.001232 | 0.078361 | IFT80 |
| rna-XM_013107802.4 | 96.90291 | 3.987021 | 1.246699 |  | 3.198063 | 0.001384 | 0.085053 | PIK3CB |
| rna-XM_013107821.4 | 57.12156 | 1.380097 | 0.586076 |  | 2.354808 | 0.018532 | 0.418229 | GPR55 |
| rna-XM_013108830.4 | 124.5414 | 1.484852 | 0.582907 |  | 2.547322 | 0.010855 | 0.313511 | MARCO |
| rna-XM_013109302.4 | 16.69698 | -7.49875 | 3.553395 |  | -2.1103 | 0.034832 | 0.560548 | SLC4A7 |
| rna-XM_013109514.4 | 15.42314 | 7.426859 | 3.551586 |  | 2.091138 | 0.036516 | 0.574047 | LOC101789992 |
| rna-XM_021266705.3 | 13.23781 | 7.207001 | 1.951219 |  | 3.693589 | 0.000221 | 0.021006 | MITF |
| rna-XM_021267052.3 | 45.69291 | 1.852464 | 0.851269 |  | 2.176122 | 0.029546 | 0.528936 | GTDC1 |
| rna-XM_021267060.3 | 21.81183 | -7.88401 | 3.362974 |  | -2.34436 | 0.01906 | 0.424642 | GTDC1 |
| rna-XM_021267411.3 | 15.50336 | -7.39161 | 3.540187 |  | -2.08792 | 0.036805 | 0.57576 | EPHA7 |
| rna-XM_021267574.3 | 4.552588 | -5.62376 | 2.664693 |  | -2.11047 | 0.034818 | NA | DUSP14 |
| rna-XM_021268343.3 | 137.7913 | -10.5434 | 2.784349 |  | -3.78668 | 0.000153 | 0.015653 | FRYL |
| rna-XM_021268513.3 | 165.5814 | -1.01598 | 0.457376 |  | -2.22131 | 0.02633 | 0.500125 | AVL9 |
| rna-XM_021269137.3 | 167.3079 | -2.8346 | 0.987341 |  | -2.87095 | 0.004092 | 0.172356 | RALA |
| rna-XM_021269187.3 | 16.78898 | 7.549238 | 3.507059 |  | 2.152584 | 0.031351 | 0.541649 | FHAD1 |
| rna-XM_021269481.3 | 95.16863 | -1.32456 | 0.472598 |  | -2.80271 | 0.005067 | 0.195835 | NVL |
| rna-XM_021269585.3 | 23.69699 | 8.046767 | 1.648213 |  | 4.882115 | 1.05E-06 | 0.000248 | ADARB2 |
| rna-XM_021269802.3 | 664.1029 | -1.94389 | 0.698377 |  | -2.78344 | 0.005379 | 0.204084 | ARHGAP6 |
| rna-XM_021270554.3 | 42.45913 | 8.888038 | 3.13393 |  | 2.836068 | 0.004567 | 0.183891 | SALL1 |
| rna-XM_021271141.3 | 24.15878 | 8.075252 | 2.12659 |  | 3.797278 | 0.000146 | 0.015207 | LOC101789532 |
| rna-XM_021271305.3 | 19.48173 | 7.763898 | 3.420849 |  | 2.269582 | 0.023233 | 0.469456 | GGT7 |
| rna-XM_021271347.3 | 15.10472 | 7.397543 | 1.918663 |  | 3.855573 | 0.000115 | 0.012781 | FBRSL1 |
| rna-XM_021271365.3 | 218.6754 | 1.166998 | 0.541111 |  | 2.15667 | 0.031031 | 0.53935 | LOC101795909 |
| rna-XM_021271373.3 | 39.06632 | -8.72496 | 3.136618 |  | -2.78165 | 0.005408 | 0.204084 | SENP7 |
| rna-XM_021271611.3 | 17.83104 | -7.5935 | 3.455418 |  | -2.19756 | 0.02798 | 0.515189 | RIMS1 |
| rna-XM_021271940.3 | 19.31057 | 7.750627 | 2.423748 |  | 3.197786 | 0.001385 | 0.085053 | THNSL1 |
| rna-XM_021271944.3 | 16.6112 | 7.534465 | 3.595696 |  | 2.095412 | 0.036134 | 0.570202 | THNSL1 |
| rna-XM_021271949.3 | 10.81614 | -6.872 | 2.158876 |  | -3.18314 | 0.001457 | 0.088034 | CCDC85A |
| rna-XM_021272163.3 | 392.6528 | 1.473326 | 0.743582 |  | 1.981391 | 0.047547 | 0.638979 | FGD3 |
| rna-XM_021272382.3 | 13.47721 | -7.1896 | 3.635354 |  | -1.97769 | 0.047964 | 0.641655 | ZBTB49 |
| rna-XM_021273077.3 | 5.411798 | 5.916803 | 2.75126 |  | 2.150579 | 0.031509 | NA | IL1RAPL2 |
| rna-XM_021273430.2 | 9.057475 | 6.659091 | 2.195596 |  | 3.032931 | 0.002422 | 0.122483 | PUS3 |
| rna-XM_021273509.3 | 15.14884 | -7.35813 | 3.585312 |  | -2.0523 | 0.040141 | 0.596426 | ADNP2 |
| rna-XM_021273714.3 | 149.3613 | -1.66128 | 0.790525 |  | -2.10148 | 0.035599 | 0.566512 | LOC101796443 |
| rna-XM_021273827.2 | 26.49728 | -5.35578 | 2.093316 |  | -2.55851 | 0.010512 | 0.309143 | TMEM62 |
| rna-XM_021274056.3 | 277.861 | -11.5554 | 1.487691 |  | -7.76733 | 8.02E-15 | 1.37E-11 | RASA3 |
| rna-XM_021274105.3 | 49.99095 | -4.26145 | 2.041256 |  | -2.08766 | 0.036828 | 0.57576 | GFRA1 |
| rna-XM_021274332.3 | 16.89842 | -7.5161 | 2.261111 |  | -3.32408 | 0.000887 | 0.061721 | SPATA1 |
| rna-XM_021274713.3 | 21.14528 | 7.882585 | 3.394589 |  | 2.322103 | 0.020227 | 0.437735 | ROR1 |
| rna-XM_021275055.3 | 27.08029 | -7.22483 | 2.690539 |  | -2.68527 | 0.007247 | 0.249028 | USP49 |
| rna-XM_021275680.3 | 22.0748 | -7.90157 | 3.395492 |  | -2.32708 | 0.019961 | 0.43459 | SCYL2 |
| rna-XM_021275754.3 | 6.396224 | 5.161291 | 2.026105 |  | 2.547395 | 0.010853 | 0.313511 | DUSP26 |
| rna-XM_021276036.3 | 7.992559 | 6.478846 | 2.254114 |  | 2.874232 | 0.00405 | 0.171688 | UROC1 |
| rna-XM_021277005.3 | 666.0539 | -1.51061 | 0.606037 |  | -2.4926 | 0.012681 | 0.340143 | LOC101801406 |
| rna-XM_021277295.3 | 300.1424 | 2.812843 | 0.961949 |  | 2.924107 | 0.003454 | 0.155244 | POU2AF1 |
| rna-XM_021277389.3 | 73.64157 | 1.892627 | 0.940389 |  | 2.012599 | 0.044157 | 0.620826 | ST3GAL1 |
| rna-XM_021277431.3 | 242.9751 | 1.006377 | 0.358559 |  | 2.80673 | 0.005005 | 0.194315 | LAT2 |
| rna-XM_021278939.3 | 22.78658 | 7.990803 | 2.242929 |  | 3.562664 | 0.000367 | 0.031309 | RAB5A |
| rna-XM_021279456.2 | 19.72755 | -7.73921 | 2.012181 |  | -3.84618 | 0.00012 | 0.013164 | BCO2 |
| rna-XM_021279670.3 | 28.7333 | 8.324477 | 3.266459 |  | 2.548472 | 0.01082 | 0.313287 | PPP1R9A |
| rna-XM_027442896.2 | 187.0342 | -4.43737 | 1.936014 |  | -2.29201 | 0.021905 | 0.455817 | EDNRB |
| rna-XM_027443175.2 | 203.3443 | 2.324469 | 0.723449 |  | 3.213039 | 0.001313 | 0.082416 | PIK3CD |
| rna-XM_027443362.2 | 54.15701 | -8.23012 | 2.584502 |  | -3.18441 | 0.00145 | 0.087931 | SLC45A1 |
| rna-XM_027443377.2 | 406.9914 | -1.01979 | 0.408712 |  | -2.49513 | 0.012591 | 0.339184 | GPR153 |
| rna-XM_027443415.2 | 90.8181 | 1.906643 | 0.819365 |  | 2.326978 | 0.019966 | 0.43459 | TNFRSF9 |
| rna-XM_027443540.2 | 229.515 | -1.57686 | 0.788736 |  | -1.99922 | 0.045584 | 0.627964 | ANK1 |
| rna-XM_027443542.2 | 29.51442 | -8.3207 | 1.593993 |  | -5.22003 | 1.79E-07 | 5.59E-05 | ANK1 |
| rna-XM_027443717.2 | 454.3954 | 3.041703 | 1.370431 |  | 2.219524 | 0.026451 | 0.500796 | RLF |
| rna-XM_027443802.2 | 236.0512 | -1.51776 | 0.648731 |  | -2.33958 | 0.019305 | 0.426891 | LOC101789501 |
| rna-XM_027443803.2 | 22.94436 | 3.731505 | 1.295416 |  | 2.880547 | 0.00397 | 0.17022 | LOC101789501 |
| rna-XM_027443867.2 | 31.99161 | -4.84102 | 1.807356 |  | -2.67851 | 0.007395 | 0.250224 | RPS6KA1 |
| rna-XM_027443869.2 | 36.48111 | 8.668742 | 1.728704 |  | 5.014591 | 5.31E-07 | 0.000141 | RPS6KA1 |
| rna-XM_027443905.2 | 18.1653 | -7.6202 | 3.451297 |  | -2.20792 | 0.02725 | 0.508837 | BIVM |
| rna-XM_027443949.2 | 1020.966 | -1.05091 | 0.448348 |  | -2.34395 | 0.019081 | 0.424642 | EDN2 |
| rna-XM_027443991.2 | 22.37996 | -7.92126 | 3.363935 |  | -2.35476 | 0.018535 | 0.418229 | EPB41 |
| rna-XM_027444041.2 | 47.49894 | -9.00687 | 3.061361 |  | -2.94211 | 0.00326 | 0.149725 | HIVEP3 |
| rna-XM_027444042.2 | 116.0006 | 1.590202 | 0.364645 |  | 4.360955 | 1.29E-05 | 0.002128 | HIVEP3 |
| rna-XM_027444144.2 | 1510.682 | -1.64075 | 0.515174 |  | -3.18486 | 0.001448 | 0.087931 | ARHGEF12 |
| rna-XM_027444174.2 | 22.44362 | -7.92518 | 3.373234 |  | -2.34943 | 0.018802 | 0.421475 | ARHGEF7 |
| rna-XM_027444699.2 | 122.6546 | 3.603998 | 0.779385 |  | 4.624156 | 3.76E-06 | 0.000735 | RASA3 |
| rna-XM_027444741.2 | 42.195 | 8.878577 | 1.914793 |  | 4.636834 | 3.54E-06 | 0.000695 | PLXNA2 |
| rna-XM_027444763.2 | 26.32904 | -8.15547 | 2.005856 |  | -4.06583 | 4.79E-05 | 0.006302 | PFKFB2 |
| rna-XM_027444869.2 | 29.08551 | 2.432703 | 1.049439 |  | 2.318098 | 0.020444 | 0.439085 | RHEX |
| rna-XM_027444875.2 | 18.20041 | 2.17488 | 1.051982 |  | 2.067411 | 0.038695 | 0.590326 | GRM4 |
| rna-XM_027445021.2 | 33.89513 | -1.32964 | 0.644478 |  | -2.06312 | 0.039101 | 0.591879 | BCAS2 |
| rna-XM_027445078.2 | 20.98252 | -4.67223 | 1.695909 |  | -2.755 | 0.005869 | 0.216407 | UBE2T |
| rna-XM_027445085.2 | 118.3223 | -1.196 | 0.338797 |  | -3.53013 | 0.000415 | 0.03418 | AP4B1 |
| rna-XM_027445166.2 | 81.89348 | 2.322101 | 1.140638 |  | 2.035791 | 0.041771 | 0.60691 | PGC |
| rna-XM_027445418.2 | 350.0585 | -1.38131 | 0.257457 |  | -5.36522 | 8.09E-08 | 2.73E-05 | GLI1 |
| rna-XM_027445459.2 | 90.60056 | 2.146871 | 0.951661 |  | 2.25592 | 0.024076 | 0.476246 | GALNT6 |
| rna-XM_027445581.2 | 7.824501 | -6.40501 | 2.262323 |  | -2.83117 | 0.004638 | 0.185442 | PHOSPHO1 |
| rna-XM_027445598.2 | 236.2489 | -1.12038 | 0.426977 |  | -2.62398 | 0.008691 | 0.274981 | RND2 |
| rna-XM_027445635.2 | 572.0997 | 1.759184 | 0.624335 |  | 2.817691 | 0.004837 | 0.190661 | IKZF3 |
| rna-XM_027445636.2 | 77.79634 | 9.761638 | 2.891281 |  | 3.376233 | 0.000735 | 0.053234 | IKZF3 |
| rna-XM_027445695.2 | 25.12333 | -8.08817 | 3.313137 |  | -2.44124 | 0.014637 | 0.36788 | ADAM11 |
| rna-XM_027445809.2 | 314.9243 | 4.217133 | 1.639297 |  | 2.572525 | 0.010096 | 0.301633 | MAP4K4 |
| rna-XM_027445850.2 | 1628.694 | -1.23792 | 0.473027 |  | -2.61702 | 0.00887 | 0.278073 | MAP4K4 |
| rna-XM_027445934.2 | 7.38142 | -6.32139 | 2.371357 |  | -2.66573 | 0.007682 | 0.255492 | IFT22 |
| rna-XM_027445972.2 | 60.73198 | -7.39944 | 2.693543 |  | -2.7471 | 0.006012 | 0.21947 | AUTS2 |
| rna-XM_027446028.2 | 16.34976 | 7.511142 | 3.538079 |  | 2.122944 | 0.033759 | 0.554394 | NPAS2 |
| rna-XM_027446150.2 | 429.8145 | -1.13539 | 0.229001 |  | -4.95802 | 7.12E-07 | 0.000181 | PTBP3 |
| rna-XM_027446151.2 | 475.0802 | 1.321334 | 0.5064 |  | 2.609271 | 0.009074 | 0.282466 | LONRF2 |
| rna-XM_027446302.2 | 185.9209 | 1.082383 | 0.413049 |  | 2.620472 | 0.008781 | 0.276426 | SMAD2 |
| rna-XM_027446316.2 | 847.5665 | -1.00829 | 0.409613 |  | -2.46156 | 0.013833 | 0.358006 | UBQLN1 |
| rna-XM_027446326.2 | 73.08065 | -1.55332 | 0.693575 |  | -2.23958 | 0.025118 | 0.489383 | GNE |
| rna-XM_027446334.2 | 86.68887 | -1.17072 | 0.513707 |  | -2.27897 | 0.022669 | 0.463469 | TRIM14 |
| rna-XM_027446375.2 | 115.9414 | -1.65104 | 0.800576 |  | -2.06232 | 0.039178 | 0.592559 | MCCC2 |
| rna-XM_027446399.2 | 32.58486 | 8.506068 | 1.995203 |  | 4.263259 | 2.01E-05 | 0.003111 | CRACDL |
| rna-XM_027446506.2 | 180.2833 | 10.97414 | 2.710028 |  | 4.049457 | 5.13E-05 | 0.006628 | INPP4A |
| rna-XM_027446520.2 | 48.1364 | -9.02623 | 3.133843 |  | -2.88024 | 0.003974 | 0.17022 | INPP4A |
| rna-XM_027446521.2 | 42.92725 | 2.874196 | 1.214643 |  | 2.366289 | 0.017967 | 0.412472 | RAB3C |
| rna-XM_027446556.2 | 56.92446 | -9.26816 | 2.199401 |  | -4.21395 | 2.51E-05 | 0.003722 | INPP4A |
| rna-XM_027446563.2 | 364.5752 | -6.8757 | 1.521538 |  | -4.51891 | 6.22E-06 | 0.001138 | INPP4A |
| rna-XM_027446785.2 | 583.7179 | 1.552354 | 0.58839 |  | 2.638308 | 0.008332 | 0.267519 | KLF4 |
| rna-XM_027446899.2 | 434.9783 | 1.201318 | 0.503965 |  | 2.383733 | 0.017138 | 0.400425 | CYFIP1 |
| rna-XM_027446903.2 | 154.8498 | -4.12255 | 1.923387 |  | -2.14338 | 0.032083 | 0.546665 | NIPA2 |
| rna-XM_027447206.2 | 35.44117 | 8.627381 | 3.195311 |  | 2.700013 | 0.006934 | 0.242711 | LOC101796277 |
| rna-XM_027447360.2 | 14.02009 | 7.289184 | 1.927214 |  | 3.782239 | 0.000155 | 0.015892 | TMEM269 |
| rna-XM_027447577.2 | 157.8353 | -1.52399 | 0.739439 |  | -2.061 | 0.039303 | 0.592771 | PPP1R14A |
| rna-XM_027447644.2 | 715.6394 | 1.286059 | 0.223311 |  | 5.75906 | 8.46E-09 | 3.69E-06 | ARHGAP6 |
| rna-XM_027448666.2 | 21.31204 | 4.339257 | 1.790313 |  | 2.423742 | 0.015362 | 0.375532 | RSPH1 |
| rna-XM_027448701.2 | 30.14157 | -1.64235 | 0.762133 |  | -2.15493 | 0.031167 | 0.539953 | CYTL1 |
| rna-XM_027448870.2 | 921.0547 | 1.288963 | 0.447937 |  | 2.877554 | 0.004008 | 0.171286 | ICOSLG |
| rna-XM_027449137.2 | 44.60645 | -4.3877 | 1.902015 |  | -2.30687 | 0.021062 | 0.445651 | RUNX1 |
| rna-XM_027449244.2 | 129.1392 | -1.1652 | 0.566526 |  | -2.05675 | 0.03971 | 0.59454 | CHPT1 |
| rna-XM_027449248.2 | 219.158 | 1.217461 | 0.528545 |  | 2.30342 | 0.021255 | 0.448596 | USP16 |
| rna-XM_027449289.2 | 17.47903 | -7.5645 | 3.558855 |  | -2.12554 | 0.033541 | 0.554394 | NRIP1 |
| rna-XM_027449298.2 | 25.65026 | -8.11793 | 3.301707 |  | -2.45871 | 0.013944 | 0.359122 | NRIP1 |
| rna-XM_027449300.2 | 559.3071 | 1.286988 | 0.589624 |  | 2.182728 | 0.029056 | 0.527234 | NRIP1 |
| rna-XM_027449374.2 | 31.08679 | -8.39576 | 1.823249 |  | -4.60484 | 4.13E-06 | 0.000798 | STYXL2 |
| rna-XM_027449375.2 | 331.0749 | -11.8083 | 1.335426 |  | -8.84233 | 9.37E-19 | 2.52E-15 | POU2F1 |
| rna-XM_027449412.2 | 24.35673 | -2.27574 | 0.918803 |  | -2.47685 | 0.013255 | 0.349641 | PRR5 |
| rna-XM_027449526.2 | 703.5227 | 1.067353 | 0.333599 |  | 3.199511 | 0.001377 | 0.084823 | NFKBIZ |
| rna-XM_027449533.2 | 47.93571 | 9.063556 | 1.505981 |  | 6.018374 | 1.76E-09 | 9.72E-07 | SENP7 |
| rna-XM_027449623.2 | 729.8675 | 1.202441 | 0.551447 |  | 2.180519 | 0.029219 | 0.528406 | UPK1B |
| rna-XM_027449699.2 | 523.4409 | 2.18984 | 0.861244 |  | 2.542648 | 0.011002 | 0.315382 | TENT5C |
| rna-XM_027449710.2 | 58.70344 | -2.44127 | 0.795385 |  | -3.0693 | 0.002146 | 0.111541 | TCP11L2 |
| rna-XM_027449847.2 | 97.30602 | 1.149692 | 0.461126 |  | 2.493229 | 0.012659 | 0.340143 | RHNO1 |
| rna-XM_027449953.2 | 4.253995 | -5.52567 | 2.774206 |  | -1.9918 | 0.046393 | NA | ARHGAP8 |
| rna-XM_027450050.2 | 27.16128 | -1.97124 | 1.004943 |  | -1.96154 | 0.049815 | 0.652242 | PACSIN2 |
| rna-XM_027450366.2 | 17.98634 | -3.76624 | 1.800721 |  | -2.09152 | 0.036481 | 0.574002 | LOC101795679 |
| rna-XM_027450421.2 | 15.40374 | -7.38247 | 3.566884 |  | -2.06972 | 0.038478 | 0.589585 | GALNT11 |
| rna-XM_027450441.2 | 32.05737 | 4.375266 | 1.778334 |  | 2.460318 | 0.013881 | 0.358006 | DNAJB6 |
| rna-XM_027450443.2 | 286.0975 | 1.256652 | 0.622932 |  | 2.017316 | 0.043663 | 0.618371 | PTPRN2 |
| rna-XM_027450459.2 | 947.7714 | -1.12595 | 0.361363 |  | -3.11584 | 0.001834 | 0.101637 | DIP2C |
| rna-XM_027450468.2 | 17.79898 | -7.59073 | 1.841303 |  | -4.12248 | 3.75E-05 | 0.005096 | ADARB2 |
| rna-XM_027450483.2 | 8.375398 | -6.50304 | 2.504838 |  | -2.59619 | 0.009426 | 0.288832 | CREM |
| rna-XM_027450500.2 | 64.92535 | -9.45777 | 3.018682 |  | -3.13308 | 0.00173 | 0.099102 | CUL2 |
| rna-XM_027450530.2 | 139.7264 | -10.5636 | 2.30707 |  | -4.57881 | 4.68E-06 | 0.000891 | NRP1 |
| rna-XM_027450562.2 | 416.4758 | 1.420962 | 0.61124 |  | 2.324719 | 0.020087 | 0.435954 | MAP3K8 |
| rna-XM_027450573.2 | 20.6757 | 7.849645 | 3.412774 |  | 2.300077 | 0.021444 | 0.449347 | ODAD2 |
| rna-XM_027450592.2 | 69.21697 | -1.22204 | 0.48778 |  | -2.50532 | 0.012234 | 0.332941 | ACBD5 |
| rna-XM_027450607.2 | 21.82397 | -7.88478 | 3.53507 |  | -2.23044 | 0.025718 | 0.494844 | ABI1 |
| rna-XM_027450736.2 | 14.03773 | -7.24822 | 3.657767 |  | -1.9816 | 0.047524 | 0.638979 | ADAM22 |
| rna-XM_027450821.2 | 60.63598 | -9.3595 | 1.598035 |  | -5.85688 | 4.72E-09 | 2.21E-06 | SLC25A13 |
| rna-XM_027450901.2 | 572.0261 | 1.005786 | 0.361577 |  | 2.781664 | 0.005408 | 0.204084 | MGAT3 |
| rna-XM_027450955.2 | 156.0296 | 1.276041 | 0.453081 |  | 2.816367 | 0.004857 | 0.191248 | OSBPL3 |
| rna-XM_027450975.2 | 5.05567 | -5.77488 | 2.660736 |  | -2.17041 | 0.029976 | NA | GRAP2 |
| rna-XM_027451149.2 | 231.8157 | -1.00679 | 0.39444 |  | -2.55246 | 0.010697 | 0.311398 | XPNPEP3 |
| rna-XM_027451176.2 | 123.4463 | 1.075067 | 0.483312 |  | 2.224377 | 0.026123 | 0.498865 | LOC101805328 |
| rna-XM_027451276.2 | 15.68711 | -7.4086 | 3.533363 |  | -2.09676 | 0.036015 | 0.569482 | ATXN1 |
| rna-XM_027451313.2 | 207.5002 | -1.03146 | 0.330338 |  | -3.12243 | 0.001794 | 0.100135 | SEC61G |
| rna-XM_027451328.2 | 13.98269 | -7.2425 | 3.612396 |  | -2.0049 | 0.044973 | 0.623498 | HECW1 |
| rna-XM_027451375.2 | 61.91248 | -5.34513 | 2.705696 |  | -1.97551 | 0.04821 | 0.643348 | GOLGA4 |
| rna-XM_027451445.2 | 15.8942 | 7.470328 | 3.532411 |  | 2.114796 | 0.034447 | 0.558247 | ELMO1 |
| rna-XM_027451560.2 | 376.1473 | 1.309031 | 0.538031 |  | 2.433003 | 0.014974 | 0.371635 | RASSF9 |
| rna-XM_027451707.2 | 133.0128 | -1.23249 | 0.562516 |  | -2.19103 | 0.02845 | 0.520767 | LOC101802187 |
| rna-XM_027451710.2 | 34.9188 | -1.39353 | 0.674246 |  | -2.0668 | 0.038753 | 0.590527 | PSMG4 |
| rna-XM_027451806.2 | 23.50212 | 8.034493 | 3.373124 |  | 2.381914 | 0.017223 | 0.400579 | MOCOS |
| rna-XM_027451862.2 | 2183.857 | 1.743773 | 0.864016 |  | 2.018219 | 0.043568 | 0.618252 | NR4A3 |
| rna-XM_027451864.2 | 203.1976 | -1.37747 | 0.324064 |  | -4.2506 | 2.13E-05 | 0.003252 | SEC61B |
| rna-XM_027451888.2 | 901.4426 | 1.055341 | 0.461486 |  | 2.28683 | 0.022206 | 0.459608 | ZNF407 |
| rna-XM_027451957.2 | 163.4851 | -1.68918 | 0.44034 |  | -3.83608 | 0.000125 | 0.013558 | GATA6 |
| rna-XM_027451976.2 | 112.113 | 1.301976 | 0.509444 |  | 2.55568 | 0.010598 | 0.309729 | THOC1 |
| rna-XM_027451988.2 | 55.1525 | -2.7675 | 0.812715 |  | -3.40525 | 0.000661 | 0.049414 | METTL4 |
| rna-XM_027452025.2 | 224.6876 | 11.29189 | 1.302637 |  | 8.668482 | 4.38E-18 | 9.67E-15 | PTPRM |
| rna-XM_027452027.2 | 91.38785 | 6.522687 | 1.15839 |  | 5.630819 | 1.79E-08 | 7.24E-06 | PTPRM |
| rna-XM_027452033.2 | 173.1429 | -1.50367 | 0.364635 |  | -4.12378 | 3.73E-05 | 0.005086 | MTCL1 |
| rna-XM_027452069.2 | 14.02724 | -6.26881 | 2.958326 |  | -2.11904 | 0.034087 | 0.556138 | CEP83 |
| rna-XM_027452079.2 | 51.84304 | -9.13343 | 1.686206 |  | -5.41656 | 6.08E-08 | 2.13E-05 | CEP83 |
| rna-XM_027452087.2 | 14.8014 | -7.32486 | 3.563374 |  | -2.0556 | 0.039822 | 0.595102 | CEP83 |
| rna-XM_027452088.2 | 198.8072 | -11.0724 | 2.314902 |  | -4.78308 | 1.73E-06 | 0.000374 | RB1CC1 |
| rna-XM_027452089.2 | 348.3242 | -1.01546 | 0.42021 |  | -2.41654 | 0.015669 | 0.380285 | RB1CC1 |
| rna-XM_027452095.2 | 36.35607 | -8.62131 | 3.143673 |  | -2.74243 | 0.006099 | 0.221111 | CEP83 |
| rna-XM_027452100.2 | 15.76545 | 7.459491 | 1.993103 |  | 3.742653 | 0.000182 | 0.018029 | SNTG1 |
| rna-XM_027452103.2 | 29.04729 | -8.29722 | 1.826628 |  | -4.54237 | 5.56E-06 | 0.001044 | CEP83 |
| rna-XM_027452221.2 | 62.35045 | -9.3993 | 1.893825 |  | -4.96313 | 6.94E-07 | 0.000177 | ATP6V1H |
| rna-XM_027452281.2 | 982.3436 | -1.06319 | 0.228804 |  | -4.64673 | 3.37E-06 | 0.000666 | CHD7 |
| rna-XM_027452306.2 | 46.08064 | 9.006127 | 1.468256 |  | 6.133893 | 8.58E-10 | 4.95E-07 | MYBL1 |
| rna-XM_027452369.2 | 107.044 | -1.23348 | 0.603293 |  | -2.04457 | 0.040897 | 0.601121 | PEX2 |
| rna-XM_027452426.2 | 33.57109 | 8.549027 | 3.17877 |  | 2.689414 | 0.007158 | 0.247553 | E2F5 |
| rna-XM_027452458.2 | 200.8155 | 3.468876 | 1.389287 |  | 2.496876 | 0.012529 | 0.338055 | RUNX1T1 |
| rna-XM_027452482.2 | 236.6746 | -1.03881 | 0.394549 |  | -2.63291 | 0.008466 | 0.270364 | LOC101793684 |
| rna-XM_027452523.2 | 896.0358 | 3.447071 | 1.421377 |  | 2.425163 | 0.015302 | 0.374817 | RNF19A |
| rna-XM_027452531.2 | 16.21266 | -2.80966 | 1.40291 |  | -2.00274 | 0.045205 | 0.625262 | ANKRD46 |
| rna-XM_027452642.1 | 429.3796 | -1.43915 | 0.673617 |  | -2.13646 | 0.032642 | 0.549611 | CCN3 |
| rna-XM_027452673.2 | 6.187622 | -6.06643 | 2.427765 |  | -2.49877 | 0.012462 | 0.337656 | LOC113842916 |
| rna-XM_027452746.2 | 1274.193 | 13.79546 | 1.280408 |  | 10.77427 | 4.55E-27 | 8.56E-23 | AGO2 |
| rna-XM_027452756.2 | 20.76056 | -7.81282 | 3.39073 |  | -2.30417 | 0.021213 | 0.447957 | SLC45A4 |
| rna-XM_027452758.2 | 10.95026 | -6.88956 | 2.30152 |  | -2.99348 | 0.002758 | 0.132862 | GPR20 |
| rna-XM_027452775.2 | 122.2005 | -10.3702 | 2.769814 |  | -3.74401 | 0.000181 | 0.018026 | CNOT4 |
| rna-XM_027452776.2 | 13.8035 | 3.698236 | 1.836631 |  | 2.013598 | 0.044052 | 0.620278 | LOC101791268 |
| rna-XM_027452901.2 | 155.068 | 1.171584 | 0.533123 |  | 2.197587 | 0.027979 | 0.515189 | LOC101798210 |
| rna-XM_027452996.2 | 141.6793 | -1.42812 | 0.600213 |  | -2.37935 | 0.017343 | 0.402062 | SYT14 |
| rna-XM_027453014.2 | 144.6815 | -3.07933 | 1.373131 |  | -2.24256 | 0.024925 | 0.487394 | RCOR3 |
| rna-XM_027453077.2 | 357.8849 | -1.20478 | 0.339221 |  | -3.55161 | 0.000383 | 0.032143 | DISP1 |
| rna-XM_027453121.2 | 269.2747 | -1.03148 | 0.420449 |  | -2.45329 | 0.014156 | 0.362207 | MARK1 |
| rna-XM_027453150.2 | 493.0666 | -1.42963 | 0.479016 |  | -2.98451 | 0.00284 | 0.135453 | LOC101793955 |
| rna-XM_027453273.2 | 22.63986 | 7.981093 | 3.342199 |  | 2.387977 | 0.016941 | 0.398325 | BCL11A |
| rna-XM_027453407.2 | 974.7034 | -1.80744 | 0.764154 |  | -2.36528 | 0.018016 | 0.412733 | SPRED2 |
| rna-XM_027453408.2 | 17.28587 | -7.54877 | 3.501463 |  | -2.15589 | 0.031092 | 0.539815 | ACTR2 |
| rna-XM_027453445.2 | 27.96857 | -8.24275 | 3.269524 |  | -2.52109 | 0.011699 | 0.324958 | B3GNT2 |
| rna-XM_027453453.2 | 10.64904 | 6.893248 | 2.32672 |  | 2.962647 | 0.00305 | 0.143246 | CHGB |
| rna-XM_027453481.2 | 218.6865 | -1.32094 | 0.49598 |  | -2.66329 | 0.007738 | 0.255861 | LOC101797715 |
| rna-XM_027453611.2 | 329.1855 | 1.45375 | 0.545909 |  | 2.66299 | 0.007745 | 0.255861 | FOXN2 |
| rna-XM_027453696.2 | 1344.928 | 1.651151 | 0.803826 |  | 2.054116 | 0.039964 | 0.595286 | ATF3 |
| rna-XM_027453768.2 | 47.27816 | -1.82995 | 0.875912 |  | -2.08919 | 0.03669 | 0.575106 | LGALS8 |
| rna-XM_027453769.2 | 587.3938 | -2.25825 | 0.615454 |  | -3.66924 | 0.000243 | 0.022708 | LGALS8 |
| rna-XM_027453782.2 | 20.90508 | -7.82301 | 3.398134 |  | -2.30215 | 0.021327 | 0.448759 | RYR2 |
| rna-XM_027453886.2 | 45.12061 | 3.481864 | 1.294951 |  | 2.688799 | 0.007171 | 0.247553 | TNFRSF13C |
| rna-XM_027453897.2 | 28.69128 | 8.322349 | 3.284659 |  | 2.533703 | 0.011286 | 0.319391 | HEATR5B |
| rna-XM_027453898.2 | 248.0782 | -2.34299 | 0.648368 |  | -3.61367 | 0.000302 | 0.026718 | EIF2AK2 |
| rna-XM_027453917.2 | 425.3155 | -1.07089 | 0.400672 |  | -2.67274 | 0.007523 | 0.252748 | SREBF2 |
| rna-XM_027454021.2 | 6.833893 | -6.20978 | 2.368672 |  | -2.62163 | 0.008751 | 0.276183 | GLP1R |
| rna-XM_027454150.2 | 40.45431 | -8.77539 | 3.090026 |  | -2.83991 | 0.004513 | 0.182279 | TRERF1 |
| rna-XM_027454166.2 | 20.26188 | -7.77771 | 3.494272 |  | -2.22585 | 0.026024 | 0.497995 | LOC101802945 |
| rna-XM_027454186.2 | 134.0566 | -5.2482 | 1.686257 |  | -3.11234 | 0.001856 | 0.102244 | SLC9A7 |
| rna-XM_027454271.2 | 236.0613 | -6.42525 | 1.583778 |  | -4.05691 | 4.97E-05 | 0.006479 | MAP3K4 |
| rna-XM_027454308.2 | 28.22457 | -8.2561 | 3.371231 |  | -2.44899 | 0.014326 | 0.364176 | SHPRH |
| rna-XM_027454335.2 | 119.5671 | -2.0361 | 0.330118 |  | -6.16777 | 6.93E-10 | 4.19E-07 | SASH1 |
| rna-XM_027454386.2 | 205.5277 | -11.1205 | 1.491206 |  | -7.45737 | 8.83E-14 | 1.18E-10 | PAWR |
| rna-XM_027454479.2 | 65.53732 | -9.47132 | 2.416997 |  | -3.91863 | 8.91E-05 | 0.010485 | BCLAF1 |
| rna-XM_027454568.2 | 60.88746 | -3.1493 | 1.306318 |  | -2.41082 | 0.015916 | 0.384094 | PTPRK |
| rna-XM_027454571.2 | 125.478 | -1.20374 | 0.580687 |  | -2.07295 | 0.038177 | 0.586715 | PTPRK |
| rna-XM_027454762.2 | 30.29949 | -8.35847 | 2.006271 |  | -4.16617 | 3.10E-05 | 0.004418 | ATG5 |
| rna-XM_027454962.2 | 128.1088 | 1.625259 | 0.78126 |  | 2.080305 | 0.037498 | 0.581878 | NRIP2 |
| rna-XM_027455335.2 | 569.1774 | 1.066334 | 0.53442 |  | 1.995312 | 0.046009 | 0.630333 | ENPP4 |
| rna-XM_027455579.2 | 543.4542 | 1.864853 | 0.485311 |  | 3.842596 | 0.000122 | 0.01328 | ZFC3H1 |
| rna-XM_027455644.2 | 10.91209 | -6.88491 | 2.12206 |  | -3.24445 | 0.001177 | 0.075486 | CFAP99 |
| rna-XM_027455660.2 | 6.359795 | 6.149034 | 2.457952 |  | 2.50169 | 0.01236 | 0.335128 | NAT8L |
| rna-XM_027455808.2 | 99.97494 | -1.65002 | 0.636354 |  | -2.59292 | 0.009516 | 0.290091 | FAM13A |
| rna-XM_027455814.2 | 89.82847 | -9.92624 | 2.934219 |  | -3.38293 | 0.000717 | 0.052257 | FAM13A |
| rna-XM_027455871.2 | 15.01065 | -7.34502 | 3.559605 |  | -2.06344 | 0.039071 | 0.591879 | EXOC6B |
| rna-XM_027456098.2 | 96.60851 | 3.435254 | 1.437681 |  | 2.389441 | 0.016874 | 0.397238 | LOC113843481 |
| rna-XM_027456300.2 | 540.6553 | 1.105441 | 0.500033 |  | 2.210736 | 0.027054 | 0.505725 | TBC1D30 |
| rna-XM_027456401.2 | 15.37965 | 7.42381 | 2.036346 |  | 3.645652 | 0.000267 | 0.024175 | AGA |
| rna-XM_027456477.2 | 14.90058 | 7.377462 | 3.67325 |  | 2.008429 | 0.044598 | 0.623056 | MARCHF1 |
| rna-XM_027456487.2 | 15.87305 | 7.46814 | 3.669204 |  | 2.035357 | 0.041815 | 0.60691 | MARCHF1 |
| rna-XM_027456606.2 | 111.5243 | -1.22174 | 0.594141 |  | -2.05631 | 0.039752 | 0.59454 | GUCY1A1 |
| rna-XM_027456607.2 | 63.83025 | 2.812775 | 1.359156 |  | 2.069502 | 0.038499 | 0.589663 | GUCY1A1 |
| rna-XM_027456632.2 | 16.89836 | -2.20249 | 0.817465 |  | -2.6943 | 0.007054 | 0.245477 | DCHS2 |
| rna-XM_027456689.2 | 153.1612 | -1.38629 | 0.639081 |  | -2.1692 | 0.030068 | 0.53243 | ZNF827 |
| rna-XM_027456731.2 | 112.0824 | -10.2455 | 2.110165 |  | -4.85529 | 1.20E-06 | 0.000277 | MAML3 |
| rna-XM_027456789.2 | 8.88885 | -6.58881 | 2.39121 |  | -2.75543 | 0.005862 | 0.216407 | PRSS12 |
| rna-XM_027456798.2 | 15.94924 | -7.43227 | 1.983404 |  | -3.74723 | 0.000179 | 0.01794 | TBCK |
| rna-XM_027457070.2 | 59.88698 | 9.383971 | 2.157014 |  | 4.350444 | 1.36E-05 | 0.002207 | CLOCK |
| rna-XM_027457129.2 | 17.32694 | 5.699004 | 2.899135 |  | 1.96576 | 0.049326 | 0.649463 | TEC |
| rna-XM_027457193.2 | 377.8802 | -1.05793 | 0.34235 |  | -3.09021 | 0.002 | 0.107376 | APBB2 |
| rna-XM_027457219.2 | 35.42598 | -8.5837 | 3.319764 |  | -2.58564 | 0.00972 | 0.294383 | N4BP2 |
| rna-XM_027457292.2 | 332.873 | 1.258122 | 0.437822 |  | 2.873596 | 0.004058 | 0.171688 | SEL1L3 |
| rna-XM_027457389.2 | 23.48455 | 8.033417 | 3.370501 |  | 2.383449 | 0.017151 | 0.400425 | SLC2A9 |
| rna-XM_027457429.2 | 259.5637 | 1.814536 | 0.670764 |  | 2.705179 | 0.006827 | 0.240313 | TBC1D14 |
| rna-XM_027457431.2 | 128.4468 | -2.88297 | 0.728908 |  | -3.95518 | 7.65E-05 | 0.009287 | TBC1D14 |
| rna-XM_027457439.2 | 724.7798 | -1.37311 | 0.412646 |  | -3.32758 | 0.000876 | 0.061216 | SORCS2 |
| rna-XM_027457440.2 | 79.7983 | 9.798292 | 1.55025 |  | 6.320459 | 2.61E-10 | 1.63E-07 | SORCS2 |
| rna-XM_027457448.2 | 930.4294 | -1.16852 | 0.247197 |  | -4.72708 | 2.28E-06 | 0.000477 | SMIM30 |
| rna-XM_027457479.2 | 935.7447 | 1.002105 | 0.326484 |  | 3.069384 | 0.002145 | 0.111541 | MGA |
| rna-XM_027457482.2 | 18.942 | -7.68053 | 1.853646 |  | -4.14347 | 3.42E-05 | 0.004737 | MGA |
| rna-XM_027457712.2 | 34.21102 | 8.576535 | 3.197073 |  | 2.682621 | 0.007305 | 0.249826 | HEATR5A |
| rna-XM_027457789.2 | 646.1472 | 1.453636 | 0.590788 |  | 2.460505 | 0.013874 | 0.358006 | BCL11B |
| rna-XM_027457815.2 | 87.96703 | 1.197743 | 0.523111 |  | 2.289654 | 0.022041 | 0.457509 | FMN1 |
| rna-XM_027457849.2 | 164.9471 | 6.645497 | 1.671995 |  | 3.974592 | 7.05E-05 | 0.00873 | MUC5AC |
| rna-XM_027458119.2 | 17.0336 | -4.36105 | 1.800896 |  | -2.4216 | 0.015452 | 0.377017 | WASL |
| rna-XM_027458151.2 | 42.69659 | 8.895905 | 3.159243 |  | 2.815834 | 0.004865 | 0.191365 | SLC25A22 |
| rna-XM_027458172.2 | 72.97917 | 1.268336 | 0.519669 |  | 2.440664 | 0.01466 | 0.36788 | ZFYVE19 |
| rna-XM_027458194.2 | 17.17988 | -7.53973 | 3.481987 |  | -2.16535 | 0.030361 | 0.533662 | BAZ2B |
| rna-XM_027458197.2 | 221.5137 | -4.6041 | 1.861716 |  | -2.47304 | 0.013397 | 0.351553 | TANC1 |
| rna-XM_027458205.2 | 40.72828 | -8.78526 | 2.228207 |  | -3.94275 | 8.06E-05 | 0.009688 | TANC1 |
| rna-XM_027458389.2 | 8.455102 | 6.559512 | 2.294828 |  | 2.85839 | 0.004258 | 0.175776 | LOC101797844 |
| rna-XM_027458457.2 | 45.20683 | -8.9355 | 2.349495 |  | -3.80316 | 0.000143 | 0.014975 | MBD5 |
| rna-XM_027458508.2 | 2261.951 | 1.024583 | 0.234267 |  | 4.373566 | 1.22E-05 | 0.00203 | PIM3 |
| rna-XM_027458515.2 | 160.448 | 1.434193 | 0.596564 |  | 2.404089 | 0.016213 | 0.388249 | VRK1 |
| rna-XM_027458536.2 | 235.0889 | -1.4615 | 0.549906 |  | -2.65773 | 0.007867 | 0.258499 | ALG12 |
| rna-XM_027458551.2 | 50.12932 | 9.127459 | 3.112767 |  | 2.932265 | 0.003365 | 0.152318 | HECTD1 |
| rna-XM_027458556.2 | 147.2768 | 9.719067 | 2.493815 |  | 3.897268 | 9.73E-05 | 0.011267 | HECTD1 |
| rna-XM_027458584.2 | 765.9941 | 2.980474 | 1.41611 |  | 2.104692 | 0.035318 | 0.564444 | STARD9 |
| rna-XM_027458610.2 | 222.5425 | -1.02275 | 0.288309 |  | -3.5474 | 0.000389 | 0.032443 | ATP5MJ |
| rna-XM_027458635.2 | 93.70713 | -1.29979 | 0.565983 |  | -2.29651 | 0.021647 | 0.452096 | TIMM9 |
| rna-XM_027458860.2 | 7.131692 | 6.31377 | 2.354362 |  | 2.681733 | 0.007324 | 0.249855 | AKAP6 |
| rna-XM_027458897.2 | 330.1421 | 1.120618 | 0.514054 |  | 2.179962 | 0.02926 | 0.528627 | KIF21A |
| rna-XM_027458977.2 | 78.45339 | -1.69314 | 0.720342 |  | -2.35046 | 0.01875 | 0.421064 | ERGIC2 |
| rna-XM_027459006.2 | 15.20543 | 7.406365 | 3.560752 |  | 2.08 | 0.037526 | 0.581878 | PLEKHA5 |
| rna-XM_027459514.2 | 21.96673 | 7.937286 | 3.446501 |  | 2.302998 | 0.021279 | 0.448759 | SNAP23 |
| rna-XM_027459572.2 | 20.85844 | -6.02002 | 2.772216 |  | -2.17155 | 0.029889 | 0.531708 | ORC4 |
| rna-XM_027459582.2 | 105.7972 | 3.086699 | 1.473515 |  | 2.094787 | 0.03619 | 0.5706 | RPAP1 |
| rna-XM_027459645.2 | 33.91992 | -7.55248 | 2.639234 |  | -2.86162 | 0.004215 | 0.174571 | PHF21A |
| rna-XM_027459733.2 | 390.0663 | -1.36035 | 0.429353 |  | -3.16837 | 0.001533 | 0.090876 | PRDM11 |
| rna-XM_027459811.2 | 17.79715 | 7.633821 | 3.525488 |  | 2.165323 | 0.030363 | 0.533662 | CAMK2G |
| rna-XM_027459822.2 | 77.69963 | 9.759954 | 2.918802 |  | 3.343822 | 0.000826 | 0.058506 | CAMK2G |
| rna-XM_027459833.2 | 14.40902 | -7.28612 | 3.617371 |  | -2.0142 | 0.043988 | 0.620089 | PLEKHA5 |
| rna-XM_027459881.2 | 21.53295 | 7.908832 | 1.769778 |  | 4.468827 | 7.86E-06 | 0.001392 | CFAP43 |
| rna-XM_027459977.2 | 21.96078 | 7.936637 | 3.430526 |  | 2.313534 | 0.020693 | 0.442208 | PCLO |
| rna-XM_027460086.2 | 45.36547 | -7.97279 | 1.498613 |  | -5.32011 | 1.04E-07 | 3.41E-05 | MARCHF8 |
| rna-XM_027460101.2 | 200.4564 | -1.96812 | 0.559596 |  | -3.51703 | 0.000436 | 0.035445 | SEMA3D |
| rna-XM_027460207.2 | 462.7857 | -12.2913 | 1.39011 |  | -8.84197 | 9.40E-19 | 2.52E-15 | ZMIZ1 |
| rna-XM_027460251.2 | 6.605427 | -4.24037 | 2.102136 |  | -2.01717 | 0.043678 | 0.618371 | LRRC20 |
| rna-XM_027460262.2 | 43.56215 | -8.88219 | 3.227464 |  | -2.75206 | 0.005922 | 0.21723 | PTPRE |
| rna-XM_027460498.2 | 35.48909 | 8.629542 | 3.140613 |  | 2.747725 | 0.006001 | 0.219268 | NT5C2 |
| rna-XM_027460499.2 | 21.88667 | 2.083213 | 0.834039 |  | 2.497741 | 0.012499 | 0.338055 | NT5C2 |
| rna-XM_027460514.2 | 251.1873 | 1.761086 | 0.72359 |  | 2.433818 | 0.014941 | 0.371498 | BLNK |
| rna-XM_027460516.2 | 185.137 | 1.696111 | 0.85559 |  | 1.982388 | 0.047436 | 0.638491 | BLNK |
| rna-XM_027460531.2 | 5.713554 | 5.993337 | 2.629412 |  | 2.279345 | 0.022647 | 0.463469 | DNTT |
| rna-XM_027460922.2 | 1026.403 | -1.15266 | 0.324685 |  | -3.5501 | 0.000385 | 0.032255 | NFE2L2 |
| rna-XM_027460927.2 | 288.7144 | 11.6536 | 1.535133 |  | 7.591265 | 3.17E-14 | 4.57E-11 | MAP3K2 |
| rna-XM_027461034.2 | 12.41396 | -7.07087 | 2.004352 |  | -3.52776 | 0.000419 | 0.034337 | STRADB |
| rna-XM_027461108.2 | 126.0245 | -10.4146 | 2.932236 |  | -3.55177 | 0.000383 | 0.032143 | NBEAL1 |
| rna-XM_027461138.2 | 23.19586 | -7.97276 | 3.33606 |  | -2.38987 | 0.016854 | 0.39702 | IKZF2 |
| rna-XM_027461180.2 | 10.49626 | 6.872009 | 2.130274 |  | 3.225881 | 0.001256 | 0.07947 | MAP3K20 |
| rna-XM_027461190.2 | 54.5511 | 9.249722 | 2.976196 |  | 3.107901 | 0.001884 | 0.103069 | GLI2 |
| rna-XM_027461335.2 | 1124.736 | 1.578365 | 0.513018 |  | 3.076627 | 0.002094 | 0.110298 | WIPF1 |
| rna-XM_027461352.2 | 14.357 | 6.344161 | 3.135236 |  | 2.023503 | 0.043021 | 0.614765 | LOC101798011 |
| rna-XM_027461435.2 | 158.0038 | 2.096794 | 0.668008 |  | 3.138878 | 0.001696 | 0.097759 | SCUBE2 |
| rna-XM_027461476.2 | 22.62367 | 7.9797 | 3.35192 |  | 2.380636 | 0.017283 | 0.401322 | PDE1A |
| rna-XM_027461510.2 | 781.8311 | 1.414206 | 0.599222 |  | 2.360071 | 0.018271 | 0.415202 | ITGB2 |
| rna-XM_027461557.2 | 88.69966 | 9.950987 | 2.837313 |  | 3.507187 | 0.000453 | 0.036625 | HNRNPA3 |
| rna-XM_027461562.2 | 6.511219 | -6.13998 | 2.386595 |  | -2.5727 | 0.010091 | 0.301633 | HNRNPA3 |
| rna-XM_027461662.2 | 237.0002 | -5.90078 | 1.786243 |  | -3.30346 | 0.000955 | 0.065515 | RNF141 |
| rna-XM_027461718.2 | 20.67637 | 7.850208 | 3.371845 |  | 2.328164 | 0.019903 | 0.433726 | HDAC4 |
| rna-XM_027461740.2 | 49.08887 | 1.512468 | 0.61923 |  | 2.442499 | 0.014586 | 0.367341 | LOC101789531 |
| rna-XM_027461749.2 | 25.40516 | -2.04732 | 0.897059 |  | -2.28225 | 0.022474 | 0.46287 | UBE2F |
| rna-XM_027462130.2 | 6.703161 | 6.2238 | 2.969957 |  | 2.095586 | 0.036119 | 0.570199 | PCBP3 |
| rna-XM_027462195.2 | 6.104086 | -6.04725 | 2.548481 |  | -2.37288 | 0.01765 | 0.407349 | BIN1 |
| rna-XM_027462199.2 | 8.517061 | -6.52777 | 2.350061 |  | -2.7777 | 0.005474 | 0.205623 | BIN1 |
| rna-XM_027462208.2 | 15.51969 | 7.436246 | 3.580071 |  | 2.077122 | 0.03779 | 0.583332 | BIN1 |
| rna-XM_027462237.2 | 326.0548 | -1.19493 | 0.369632 |  | -3.23277 | 0.001226 | 0.078107 | ADARB1 |
| rna-XM_027462254.2 | 342.5443 | 1.224094 | 0.218307 |  | 5.6072 | 2.06E-08 | 8.21E-06 | TEAD1 |
| rna-XM_027462287.2 | 71.09548 | 1.374726 | 0.56563 |  | 2.430435 | 0.015081 | 0.371635 | ZDBF2 |
| rna-XM_027462349.2 | 86.59028 | -1.23943 | 0.543884 |  | -2.27884 | 0.022676 | 0.463469 | CNN3 |
| rna-XM_027462358.2 | 126.0183 | -7.8634 | 2.519916 |  | -3.1205 | 0.001805 | 0.100518 | ARHGAP29 |
| rna-XM_027462372.2 | 34.16644 | 1.455189 | 0.725303 |  | 2.006318 | 0.044822 | 0.623498 | RABGGTB |
| rna-XM_027462398.2 | 17.31913 | 7.594093 | 3.489741 |  | 2.17612 | 0.029546 | 0.528936 | DNAJC6 |
| rna-XM_027462409.2 | 121.8688 | -1.00384 | 0.412618 |  | -2.43286 | 0.01498 | 0.371635 | ADGRL4 |
| rna-XM_027462490.2 | 58.40244 | -5.62937 | 2.673055 |  | -2.10597 | 0.035207 | 0.563863 | CCSER2 |
| rna-XM_027462526.2 | 761.7291 | -1.80321 | 0.811185 |  | -2.22293 | 0.026221 | 0.499464 | SERBP1 |
| rna-XM_027462614.2 | 15.15065 | 7.401787 | 2.042827 |  | 3.623306 | 0.000291 | 0.025987 | ABCA4 |
| rna-XM_027462712.2 | 5.848759 | 6.028247 | 2.570372 |  | 2.345282 | 0.019013 | 0.424031 | NR5A2 |
| rna-XM_027462786.2 | 428.3483 | -1.02155 | 0.250286 |  | -4.08153 | 4.47E-05 | 0.005953 | LOC101795759 |
| rna-XM_027462875.2 | 22.31037 | -7.91688 | 3.476563 |  | -2.27721 | 0.022773 | 0.464424 | GLIS1 |
| rna-XM_027462918.2 | 31.88613 | -8.43185 | 3.194087 |  | -2.63983 | 0.008295 | 0.267378 | ZBTB20 |
| rna-XM_027462948.2 | 314.9027 | 1.463815 | 0.667879 |  | 2.191736 | 0.028399 | 0.520086 | ARID5B |
| rna-XM_027463187.2 | 5371.75 | 1.702168 | 0.669966 |  | 2.54068 | 0.011064 | 0.316197 | CCN1 |
| rna-XM_027463281.2 | 6.815456 | 6.247663 | 2.618279 |  | 2.386171 | 0.017025 | 0.399036 | NTNG1 |
| rna-XM_027463295.2 | 32.60329 | -8.46446 | 1.763936 |  | -4.79862 | 1.60E-06 | 0.000349 | CACNA1C |
| rna-XM_027463300.2 | 40.34045 | -8.7713 | 3.084513 |  | -2.84366 | 0.00446 | 0.180559 | CACNA1C |
| rna-XM_027463406.2 | 162.5424 | -1.06348 | 0.454142 |  | -2.34172 | 0.019195 | 0.425955 | ZFYVE9 |
| rna-XM_027463423.2 | 985.7244 | 2.882591 | 1.082907 |  | 2.661902 | 0.00777 | 0.256185 | TGFBR3 |
| rna-XM_027463458.2 | 71.63182 | 8.677606 | 2.524083 |  | 3.437925 | 0.000586 | 0.045354 | RABGAP1L |
| rna-XM_027463508.2 | 610.1643 | -5.87533 | 1.447167 |  | -4.05988 | 4.91E-05 | 0.006419 | ERC1 |
| rna-XM_027463582.2 | 55.82899 | -1.67201 | 0.795202 |  | -2.10263 | 0.035499 | 0.565716 | C8H1orf112 |
| rna-XM_027463616.2 | 1364.3 | 1.066815 | 0.327254 |  | 3.259894 | 0.001115 | 0.072484 | LOC101798767 |
| rna-XM_027463702.2 | 31.15944 | 6.623036 | 2.701626 |  | 2.4515 | 0.014226 | 0.362662 | KMT2E |
| rna-XM_027463713.2 | 132.0918 | 10.5252 | 1.405001 |  | 7.491239 | 6.82E-14 | 9.48E-11 | KMT2E |
| rna-XM_027463716.2 | 231.3921 | 1.09323 | 0.360048 |  | 3.036341 | 0.002395 | 0.121572 | KMT2E |
| rna-XM_027463736.2 | 3355.874 | -2.18517 | 0.525299 |  | -4.15986 | 3.18E-05 | 0.004509 | KMT2E |
| rna-XM_027463791.2 | 18.25336 | 7.66979 | 3.52886 |  | 2.173447 | 0.029747 | 0.530602 | RAVER2 |
| rna-XM_027463799.2 | 227.8521 | 2.345581 | 0.757036 |  | 3.098376 | 0.001946 | 0.105062 | EGR2 |
| rna-XM_027463908.2 | 46.76955 | -8.98492 | 1.577828 |  | -5.69448 | 1.24E-08 | 5.22E-06 | LXN |
| rna-XM_027463912.2 | 430.5398 | 1.221365 | 0.518329 |  | 2.356353 | 0.018455 | 0.417947 | EHHADH |
| rna-XM_027463934.2 | 16.72907 | -7.5017 | 1.853325 |  | -4.0477 | 5.17E-05 | 0.006647 | TFDP2 |
| rna-XM_027463970.2 | 455.6573 | -1.56872 | 0.542407 |  | -2.89215 | 0.003826 | 0.165793 | CAB39 |
| rna-XM_027464012.2 | 298.5941 | 1.26359 | 0.255511 |  | 4.945346 | 7.60E-07 | 0.000191 | VPS8 |
| rna-XM_027464026.2 | 342.109 | -2.24829 | 0.57645 |  | -3.90023 | 9.61E-05 | 0.011165 | ACSL3 |
| rna-XM_027464109.2 | 256.7908 | -1.04323 | 0.405698 |  | -2.57145 | 0.010127 | 0.302327 | ARMC9 |
| rna-XM_027464152.2 | 399.5375 | -1.74302 | 0.459393 |  | -3.79417 | 0.000148 | 0.015272 | PCYT1A |
| rna-XM_027464186.2 | 11.31055 | -6.93688 | 2.127075 |  | -3.26123 | 0.001109 | 0.072393 | DVL3 |
| rna-XM_027464187.2 | 15.91074 | 7.471975 | 1.882821 |  | 3.9685 | 7.23E-05 | 0.008899 | DVL3 |
| rna-XM_027464210.2 | 13.56827 | -7.19919 | 3.63411 |  | -1.98101 | 0.047591 | 0.638979 | AP2M1 |
| rna-XM_027464265.2 | 22.6692 | -7.94018 | 1.832531 |  | -4.3329 | 1.47E-05 | 0.00234 | SERPINI1 |
| rna-XM_027464266.2 | 217.9819 | -1.84797 | 0.84077 |  | -2.19795 | 0.027953 | 0.515189 | SERPINI1 |
| rna-XM_027464391.2 | 7.018186 | -6.24877 | 2.435317 |  | -2.5659 | 0.010291 | 0.305813 | SLC35G2 |
| rna-XM_027464415.2 | 132.4637 | -1.13309 | 0.426821 |  | -2.65471 | 0.007938 | 0.260597 | HES6 |
| rna-XM_027464464.2 | 84.12658 | -4.12479 | 1.705492 |  | -2.41853 | 0.015583 | 0.378979 | GIGYF2 |
| rna-XM_027464584.2 | 267.1155 | 1.065934 | 0.484186 |  | 2.201496 | 0.027701 | 0.51231 | GPR171 |
| rna-XM_027464638.2 | 300.2639 | -1.17141 | 0.528179 |  | -2.21783 | 0.026566 | 0.501712 | MBNL1 |
| rna-XM_027464643.2 | 315.7166 | -11.7397 | 1.22036 |  | -9.61989 | 6.59E-22 | 2.92E-18 | STAG1 |
| rna-XM_027464817.2 | 1138.647 | 1.467213 | 0.626009 |  | 2.343759 | 0.01909 | 0.424642 | DOCK10 |
| rna-XM_027464868.2 | 134.7352 | -10.5113 | 1.444817 |  | -7.27521 | 3.46E-13 | 4.06E-10 | ATP11C |
| rna-XM_027464978.2 | 272.608 | 1.246523 | 0.423606 |  | 2.94265 | 0.003254 | 0.149725 | LAMP2 |
| rna-XM_027465173.2 | 21.48263 | 3.306334 | 1.459385 |  | 2.265566 | 0.023478 | 0.471881 | FRMPD3 |
| rna-XM_027465257.2 | 614.5237 | 1.225372 | 0.620627 |  | 1.97441 | 0.048335 | 0.644097 | SEPTIN6 |
| rna-XM_027465267.2 | 16.06711 | -7.44301 | 3.545909 |  | -2.09904 | 0.035813 | 0.568798 | KIAA1210 |
| rna-XM_027465319.2 | 19.1371 | -7.69526 | 3.430633 |  | -2.2431 | 0.02489 | 0.487223 | LOC101796377 |
| rna-XM_027465327.2 | 468.5046 | -1.24015 | 0.580629 |  | -2.13587 | 0.03269 | 0.549611 | PHF6 |
| rna-XM_027465429.2 | 207.5214 | 1.980998 | 0.775745 |  | 2.553673 | 0.010659 | 0.310552 | COL4A5 |
| rna-XM_027465502.2 | 37.39952 | -7.6941 | 2.21852 |  | -3.46812 | 0.000524 | 0.041145 | LOC101802145 |
| rna-XM_027465653.2 | 302.2437 | -1.7187 | 0.627334 |  | -2.7397 | 0.00615 | 0.221996 | LINGO1 |
| rna-XM_027465703.2 | 307.6628 | -4.74533 | 1.913925 |  | -2.47937 | 0.013161 | 0.348541 | TMED3 |
| rna-XM_027465705.2 | 27.148 | -4.10838 | 1.899969 |  | -2.16234 | 0.030592 | 0.535533 | USP18 |
| rna-XM_027465713.2 | 17.8997 | 3.869251 | 1.930791 |  | 2.003973 | 0.045073 | 0.62412 | MAP2K5 |
| rna-XM_027465835.2 | 5.016955 | -5.76404 | 2.697073 |  | -2.13715 | 0.032586 | NA | LCMT2 |
| rna-XM_027465892.2 | 12.56877 | 7.131833 | 2.191819 |  | 3.253843 | 0.001139 | 0.073662 | MEF2A |
| rna-XM_027465964.2 | 54.10541 | 2.108005 | 0.894892 |  | 2.355599 | 0.018493 | 0.418229 | TPPP3 |
| rna-XM_027465984.2 | 41.51332 | 8.855361 | 3.154604 |  | 2.807123 | 0.004999 | 0.194315 | WDR59 |
| rna-XM_027465989.2 | 33.0742 | -8.48494 | 1.649289 |  | -5.14461 | 2.68E-07 | 7.86E-05 | NUP93 |
| rna-XM_027465992.2 | 39.86944 | 8.797129 | 3.104842 |  | 2.833358 | 0.004606 | 0.184641 | CHD2 |
| rna-XM_027466038.2 | 907.8183 | -1.63582 | 0.427534 |  | -3.82618 | 0.00013 | 0.013994 | SCAMP2 |
| rna-XM_027466050.2 | 8.048083 | -6.44603 | 2.398282 |  | -2.68777 | 0.007193 | 0.248088 | MCTP2 |
| rna-XM_027466068.2 | 42.25 | -8.8379 | 3.080414 |  | -2.86906 | 0.004117 | 0.172997 | C11H15orf40 |
| rna-XM_027466326.2 | 153.1784 | -1.09716 | 0.445217 |  | -2.46432 | 0.013727 | 0.357525 | E2F4 |
| rna-XM_027466451.2 | 15.59464 | 3.63787 | 1.776039 |  | 2.048305 | 0.04053 | 0.599485 | SAXO2 |
| rna-XM_027466566.2 | 77.27952 | 9.752176 | 2.880645 |  | 3.385414 | 0.000711 | 0.052089 | TPM1 |
| rna-XM_027466619.2 | 698.4026 | -1.23007 | 0.324125 |  | -3.79504 | 0.000148 | 0.01526 | UBE2Q2 |
| rna-XM_027466625.2 | 194.4709 | 1.58542 | 0.515879 |  | 3.073241 | 0.002117 | 0.110666 | EDC3 |
| rna-XM_027466725.2 | 39.34634 | -8.73524 | 1.713388 |  | -5.09823 | 3.43E-07 | 9.68E-05 | ZFAND6 |
| rna-XM_027466857.2 | 1261.266 | -1.50737 | 0.481973 |  | -3.12749 | 0.001763 | 0.099714 | LOC101792155 |
| rna-XM_027466888.2 | 395.9288 | 1.029992 | 0.342131 |  | 3.01052 | 0.002608 | 0.128433 | MYEF2 |
| rna-XM_027466913.2 | 247.1727 | -1.77902 | 0.366848 |  | -4.84947 | 1.24E-06 | 0.000283 | MEAK7 |
| rna-XM_027466958.2 | 47.04532 | -1.97408 | 0.660332 |  | -2.98952 | 0.002794 | 0.134252 | KCTD15 |
| rna-XM_027467043.2 | 205.2541 | -1.59119 | 0.544516 |  | -2.92221 | 0.003476 | 0.155943 | ME3 |
| rna-XM_027467110.2 | 53.11943 | -5.77865 | 2.218441 |  | -2.60482 | 0.009192 | 0.283663 | SLTM |
| rna-XM_027467114.2 | 15.37456 | 5.577356 | 2.04101 |  | 2.732645 | 0.006283 | 0.225179 | SLTM |
| rna-XM_027467188.2 | 6.293744 | -6.09055 | 2.749859 |  | -2.21486 | 0.02677 | 0.503525 | TCF12 |
| rna-XM_027467239.2 | 80.6615 | -1.62894 | 0.805682 |  | -2.02181 | 0.043196 | 0.616088 | NOX4 |
| rna-XM_027467282.2 | 183.3679 | 1.14474 | 0.56818 |  | 2.014747 | 0.043931 | 0.620089 | NISCH |
| rna-XM_027467310.2 | 74.18604 | 1.110914 | 0.559746 |  | 1.984674 | 0.047181 | 0.636927 | SFMBT1 |
| rna-XM_027467331.2 | 11.48802 | -4.03031 | 1.898179 |  | -2.12325 | 0.033733 | 0.554394 | SLC41A3 |
| rna-XM_027467336.2 | 513.4818 | -1.24786 | 0.404652 |  | -3.08379 | 0.002044 | 0.108784 | TMEM115 |
| rna-XM_027467344.2 | 21.48849 | 1.693679 | 0.862402 |  | 1.963908 | 0.049541 | 0.651008 | DEUP1 |
| rna-XM_027467426.2 | 609.7095 | 1.649823 | 0.587846 |  | 2.806557 | 0.005007 | 0.194315 | BHLHE40 |
| rna-XM_027467552.2 | 158.1382 | -1.25378 | 0.577615 |  | -2.17061 | 0.029961 | 0.531708 | LOC101792450 |
| rna-XM_027467556.2 | 235.2888 | 3.269102 | 1.416298 |  | 2.308203 | 0.020988 | 0.445651 | TASOR |
| rna-XM_027467566.2 | 66.82858 | 1.302655 | 0.565882 |  | 2.301992 | 0.021336 | 0.448759 | ERC2 |
| rna-XM_027467713.2 | 931.7193 | 1.0312 | 0.464597 |  | 2.219557 | 0.026449 | 0.500796 | MAGI1 |
| rna-XM_027467721.2 | 238.365 | 2.963151 | 1.382905 |  | 2.1427 | 0.032137 | 0.546665 | MAGI1 |
| rna-XM_027467824.2 | 393.2578 | -1.37684 | 0.536607 |  | -2.56583 | 0.010293 | 0.305813 | PGR |
| rna-XM_027467843.2 | 29.08138 | 8.341602 | 2.146354 |  | 3.886407 | 0.000102 | 0.011711 | PGR |
| rna-XM_027467906.2 | 44.68434 | -8.91882 | 3.065891 |  | -2.90905 | 0.003625 | 0.160048 | LRIG1 |
| rna-XM_027467948.2 | 44.88006 | -8.92502 | 3.061231 |  | -2.9155 | 0.003551 | 0.158452 | IQSEC3 |
| rna-XM_027467962.2 | 13.02226 | -7.14033 | 2.650973 |  | -2.69348 | 0.007071 | 0.245688 | LOC101797103 |
| rna-XM_027467997.2 | 355.3581 | 1.01088 | 0.416941 |  | 2.424513 | 0.015329 | 0.375224 | XPC |
| rna-XM_027467998.2 | 25.62689 | -8.1166 | 3.318657 |  | -2.44575 | 0.014455 | 0.366337 | YAP1 |
| rna-XM_027468063.2 | 235.0501 | 1.641911 | 0.362368 |  | 4.531061 | 5.87E-06 | 0.001092 | SEPTIN8 |
| rna-XM_027468105.2 | 28.97169 | -8.29355 | 3.253182 |  | -2.54936 | 0.010792 | 0.3132 | LOC101795967 |
| rna-XM_027468226.2 | 238.5287 | 2.022446 | 0.77889 |  | 2.596575 | 0.009416 | 0.288832 | RNF145 |
| rna-XM_027468246.2 | 38.27828 | 8.738442 | 3.128235 |  | 2.79341 | 0.005216 | 0.200116 | MSANTD4 |
| rna-XM_027468264.2 | 13.72057 | 6.279666 | 2.887693 |  | 2.17463 | 0.029658 | 0.529453 | RANBP17 |
| rna-XM_027468300.2 | 47.59805 | 9.052759 | 3.070687 |  | 2.948122 | 0.003197 | 0.14823 | AGTR1 |
| rna-XM_027468422.2 | 117.0936 | 2.41142 | 1.182067 |  | 2.040004 | 0.04135 | 0.604446 | MZB1 |
| rna-XM_027468454.2 | 20304.1 | 2.364408 | 0.756309 |  | 3.126247 | 0.001771 | 0.099714 | EGR1 |
| rna-XM_027468522.2 | 12.9188 | 7.171395 | 3.652996 |  | 1.963155 | 0.049628 | 0.651609 | KCTD16 |
| rna-XM_027468525.2 | 121.7103 | 1.312537 | 0.488616 |  | 2.686235 | 0.007226 | 0.248774 | EXPH5 |
| rna-XM_027468547.2 | 79.99926 | -1.38784 | 0.511294 |  | -2.71437 | 0.00664 | 0.235071 | LOC113845171 |
| rna-XM_027468577.2 | 84.87083 | 1.596617 | 0.484702 |  | 3.294018 | 0.000988 | 0.066539 | RNF130 |
| rna-XM_027468654.2 | 54.8915 | -1.32943 | 0.47301 |  | -2.81057 | 0.004945 | 0.193477 | DND1 |
| rna-XM_027468743.2 | 524.832 | 6.855995 | 1.650361 |  | 4.154239 | 3.26E-05 | 0.004587 | ARHGAP17 |
| rna-XM_027468744.2 | 173.7627 | 1.268117 | 0.62331 |  | 2.034487 | 0.041902 | 0.607175 | ARHGAP17 |
| rna-XM_027468794.2 | 33.43659 | -1.7104 | 0.541187 |  | -3.16046 | 0.001575 | 0.092941 | VPS35L |
| rna-XM_027468851.2 | 14.26575 | 7.313908 | 2.076904 |  | 3.521543 | 0.000429 | 0.034924 | SYNGR3 |
| rna-XM_027468856.2 | 26.70898 | -8.17626 | 3.274422 |  | -2.49701 | 0.012525 | 0.338055 | E4F1 |
| rna-XM_027468882.2 | 152.4939 | -1.20695 | 0.368752 |  | -3.27306 | 0.001064 | 0.070534 | PDGFA |
| rna-XM_027468989.2 | 8.858698 | -2.92174 | 1.456559 |  | -2.00592 | 0.044865 | 0.623498 | IQCE |
| rna-XM_027469006.2 | 50.8944 | -2.47619 | 0.649935 |  | -3.8099 | 0.000139 | 0.014696 | MARF1 |
| rna-XM_027469065.2 | 51.0022 | -1.56132 | 0.52942 |  | -2.94912 | 0.003187 | 0.148003 | ELFN1 |
| rna-XM_027469069.2 | 74.97131 | -1.92912 | 0.757878 |  | -2.54542 | 0.010915 | 0.31409 | CACNG3 |
| rna-XM_027469133.2 | 59.42153 | -9.33005 | 2.971406 |  | -3.13994 | 0.00169 | 0.097554 | LOC101799012 |
| rna-XM_027469166.2 | 151.7075 | 3.992506 | 1.986828 |  | 2.009487 | 0.044486 | 0.622486 | SMURF1 |
| rna-XM_027469271.2 | 389.12 | 1.978998 | 0.845767 |  | 2.339885 | 0.01929 | 0.426795 | LOC101801498 |
| rna-XM_027469283.2 | 1722.165 | 1.378164 | 0.382982 |  | 3.598508 | 0.00032 | 0.02806 | LMTK2 |
| rna-XM_027469345.2 | 105.7152 | 2.001313 | 0.7138 |  | 2.803743 | 0.005051 | 0.195615 | CIITA |
| rna-XM_027469349.2 | 67.93421 | 2.531381 | 1.146816 |  | 2.207313 | 0.027292 | 0.509013 | LOC101798643 |
| rna-XM_027469476.2 | 10.18082 | -6.78461 | 2.155352 |  | -3.1478 | 0.001645 | 0.095856 | CPPED1 |
| rna-XM_027469479.2 | 13.91231 | -7.23546 | 3.60667 |  | -2.00613 | 0.044842 | 0.623498 | DCUN1D3 |
| rna-XM_027469504.2 | 517.8638 | -12.4536 | 1.286061 |  | -9.68348 | 3.54E-22 | 1.90E-18 | MRTFB |
| rna-XM_027469512.2 | 535.3889 | 12.54439 | 3.906843 |  | 3.210877 | 0.001323 | 0.0829 | MRTFB |
| rna-XM_027469692.2 | 70.10456 | 4.749219 | 1.316336 |  | 3.607909 | 0.000309 | 0.027254 | DEPDC5 |
| rna-XM_027469730.2 | 57.66742 | -9.28683 | 2.965338 |  | -3.1318 | 0.001737 | 0.099218 | PITPNM2 |
| rna-XM_027469734.2 | 32.39511 | -8.45459 | 1.658371 |  | -5.09813 | 3.43E-07 | 9.68E-05 | PITPNM2 |
| rna-XM_027469835.2 | 40.31088 | 8.813143 | 3.087883 |  | 2.854106 | 0.004316 | 0.176996 | ARVCF |
| rna-XM_027469900.2 | 53.64786 | -9.1829 | 1.560035 |  | -5.88634 | 3.95E-09 | 1.95E-06 | SLC15A4 |
| rna-XM_027469904.2 | 56.41946 | 5.475571 | 2.701703 |  | 2.026711 | 0.042692 | 0.612392 | CIT |
| rna-XM_027470003.2 | 14.76951 | 4.050934 | 2.061847 |  | 1.964711 | 0.049448 | 0.650563 | GALNT9 |
| rna-XM_027470054.2 | 112.6213 | 1.074364 | 0.396118 |  | 2.712232 | 0.006683 | 0.235772 | RNF185 |
| rna-XM_027470275.2 | 744.9744 | -12.9782 | 1.302854 |  | -9.96135 | 2.25E-23 | 2.10E-19 | TAOK3 |
| rna-XM_027470455.2 | 15.99126 | 7.479559 | 3.747557 |  | 1.995849 | 0.04595 | 0.630222 | LOC101804334 |
| rna-XM_027470500.2 | 8.498165 | -6.52459 | 2.432141 |  | -2.68265 | 0.007304 | 0.249826 | LOC101789992 |
| rna-XM_027470580.2 | 100.0786 | -10.0821 | 1.742379 |  | -5.7864 | 7.19E-09 | 3.29E-06 | BRD3 |
| rna-XM_027470618.2 | 55.25605 | 3.983907 | 1.263875 |  | 3.152136 | 0.001621 | 0.09461 | SPACA9 |
| rna-XM_027470779.2 | 894.9287 | 1.042083 | 0.465478 |  | 2.238734 | 0.025173 | 0.489443 | LOC101799454 |
| rna-XM_027470797.2 | 13.81222 | -7.22501 | 3.613213 |  | -1.99961 | 0.045543 | 0.627842 | ADAMTSL2 |
| rna-XM_027470876.2 | 59.84636 | -1.17004 | 0.507142 |  | -2.30712 | 0.021048 | 0.445651 | LOC101794119 |
| rna-XM_027470933.2 | 362.5464 | -1.07446 | 0.425891 |  | -2.52285 | 0.011641 | 0.324296 | SLC25A15 |
| rna-XM_027470954.2 | 301.236 | 1.285806 | 0.513202 |  | 2.505459 | 0.012229 | 0.332941 | TPRN |
| rna-XM_027471011.2 | 30.12642 | 4.536227 | 1.832729 |  | 2.475122 | 0.013319 | 0.350489 | TRIM32 |
| rna-XM_027471208.2 | 121.9009 | -1.83221 | 0.371451 |  | -4.93257 | 8.12E-07 | 0.0002 | LOC101791127 |
| rna-XM_027471214.2 | 30.69889 | 8.420359 | 3.187377 |  | 2.641783 | 0.008247 | 0.266556 | USP20 |
| rna-XM_027471216.2 | 144.0078 | -1.1661 | 0.553709 |  | -2.10598 | 0.035206 | 0.563863 | USP20 |
| rna-XM_027471293.2 | 280.6867 | -1.2551 | 0.259387 |  | -4.83872 | 1.31E-06 | 0.000297 | TRIM47 |
| rna-XM_027471327.2 | 67.86672 | -9.52159 | 1.430571 |  | -6.6558 | 2.82E-11 | 2.16E-08 | CEP112 |
| rna-XM_027471336.2 | 89.14964 | 6.520515 | 1.467179 |  | 4.444252 | 8.82E-06 | 0.001554 | AXIN2 |
| rna-XM_027471393.2 | 37.83731 | 8.721347 | 1.774376 |  | 4.915163 | 8.87E-07 | 0.000216 | PIK3R5 |
| rna-XM_027471394.2 | 541.1337 | 1.138366 | 0.474639 |  | 2.398381 | 0.016468 | 0.391356 | PIK3R5 |
| rna-XM_027471395.2 | 34.18898 | -8.53252 | 3.164688 |  | -2.69616 | 0.007014 | 0.24462 | PIK3R5 |
| rna-XM_027471492.2 | 12.2228 | -6.06817 | 2.945727 |  | -2.05999 | 0.0394 | 0.593415 | UTS2R |
| rna-XM_027471557.2 | 135.8507 | 1.201621 | 0.369834 |  | 3.249081 | 0.001158 | 0.074516 | NDEL1 |
| rna-XM_027471572.2 | 113.8933 | -1.96846 | 0.620619 |  | -3.17178 | 0.001515 | 0.090245 | ASPSCR1 |
| rna-XM_027471668.2 | 245.6579 | -1.34023 | 0.50105 |  | -2.67485 | 0.007476 | 0.251507 | HELZ |
| rna-XM_027471774.2 | 1261.674 | -1.00968 | 0.123037 |  | -8.20632 | 2.28E-16 | 4.50E-13 | CPAMD8 |
| rna-XM_027471912.2 | 457.9382 | -2.20476 | 0.905085 |  | -2.43597 | 0.014852 | 0.369946 | VTN |
| rna-XM_027471976.2 | 71.04503 | 1.262553 | 0.573157 |  | 2.202806 | 0.027608 | 0.511368 | DPP9 |
| rna-XM_027472023.2 | 57.69442 | 9.33054 | 1.416112 |  | 6.588845 | 4.43E-11 | 3.02E-08 | CUEDC1 |
| rna-XM_027472072.2 | 13.57903 | 7.243063 | 2.138484 |  | 3.387007 | 0.000707 | 0.05199 | MFSD12 |
| rna-XM_027472165.2 | 24.88608 | 3.762725 | 1.894907 |  | 1.985704 | 0.047066 | 0.636683 | LINGO3 |
| rna-XM_027472177.2 | 14.10407 | -7.25507 | 2.956017 |  | -2.45434 | 0.014114 | 0.362207 | PIPOX |
| rna-XM_027472375.2 | 24.787 | 8.111319 | 3.328152 |  | 2.437184 | 0.014802 | 0.369315 | PDE4C |
| rna-XM_027472461.1 | 175.5603 | -2.13306 | 0.808108 |  | -2.63957 | 0.008301 | 0.267378 | SDF2 |
| rna-XM_027472515.2 | 276.7093 | 2.521238 | 0.790527 |  | 3.189314 | 0.001426 | 0.087016 | SBNO2 |
| rna-XM_027472638.2 | 213.6748 | 5.889803 | 1.884013 |  | 3.126201 | 0.001771 | 0.099714 | HNRNPM |
| rna-XM_027472682.2 | 82.91992 | -1.12034 | 0.431267 |  | -2.59778 | 0.009383 | 0.288129 | NLE1 |
| rna-XM_027472704.2 | 298.156 | 1.207408 | 0.572103 |  | 2.110471 | 0.034818 | 0.560548 | FAM222B |
| rna-XM_027472723.2 | 405.1952 | 2.157598 | 0.324765 |  | 6.643559 | 3.06E-11 | 2.30E-08 | NCOA6 |
| rna-XM_027472774.2 | 32.13045 | 2.774591 | 0.74534 |  | 3.722582 | 0.000197 | 0.019196 | LOC101799803 |
| rna-XM_027472883.2 | 49.04089 | -9.05285 | 1.68405 |  | -5.37564 | 7.63E-08 | 2.60E-05 | SLC35C2 |
| rna-XM_027472947.2 | 74.42343 | 9.697836 | 2.877876 |  | 3.369789 | 0.000752 | 0.054285 | SPATA2 |
| rna-XM_027473169.2 | 107.0795 | 1.854391 | 0.915737 |  | 2.025027 | 0.042865 | 0.614063 | HRH3 |
| rna-XM_027473276.2 | 5.084936 | -5.78302 | 2.683025 |  | -2.15541 | 0.03113 | NA | SNPH |
| rna-XM_038164909.1 | 8.277155 | 6.528861 | 2.241933 |  | 2.912157 | 0.003589 | 0.1594 | TRABD |
| rna-XM_038164924.1 | 304.5225 | 1.07196 | 0.527218 |  | 2.033239 | 0.042028 | 0.608455 | LOC101803784 |
| rna-XM_038164930.1 | 1595.698 | -1.69573 | 0.36488 |  | -4.64736 | 3.36E-06 | 0.000666 | GNPTAB |
| rna-XM_038165010.1 | 4.542508 | 5.662128 | 2.871506 |  | 1.971832 | 0.048629 | NA | TMEM268 |
| rna-XM_038165111.1 | 396.7113 | -1.35247 | 0.631126 |  | -2.14296 | 0.032117 | 0.546665 | SPTAN1 |
| rna-XM_038165192.1 | 39.98591 | -1.94766 | 0.984984 |  | -1.97735 | 0.048002 | 0.64169 | TMPRSS6 |
| rna-XM_038165213.1 | 7.976216 | -6.43278 | 2.332408 |  | -2.758 | 0.005816 | 0.215643 | SEPTIN9 |
| rna-XM_038165226.1 | 204.9492 | -1.88091 | 0.547606 |  | -3.43478 | 0.000593 | 0.045695 | BPTF |
| rna-XM_038165229.1 | 114.5645 | -8.48378 | 2.225355 |  | -3.81233 | 0.000138 | 0.014593 | BPTF |
| rna-XM_038165230.1 | 92.63178 | -9.97058 | 2.995419 |  | -3.32861 | 0.000873 | 0.061216 | BPTF |
| rna-XM_038165339.1 | 74.63688 | -9.6589 | 2.90131 |  | -3.32915 | 0.000871 | 0.061214 | MAP2K6 |
| rna-XM_038165354.1 | 11.46264 | -6.95584 | 2.301074 |  | -3.02287 | 0.002504 | 0.125541 | LLGL2 |
| rna-XM_038165384.1 | 3066.526 | 1.132419 | 0.211598 |  | 5.35175 | 8.71E-08 | 2.92E-05 | GAS7 |
| rna-XM_038165386.1 | 66.93445 | -9.50179 | 1.644194 |  | -5.77899 | 7.51E-09 | 3.40E-06 | GAS7 |
| rna-XM_038165499.1 | 18.18616 | 7.66463 | 3.457473 |  | 2.21683 | 0.026635 | 0.502497 | FOXJ1 |
| rna-XM_038165512.1 | 272.2037 | 1.368949 | 0.592159 |  | 2.311791 | 0.020789 | 0.443248 | CASKIN2 |
| rna-XM_038165522.1 | 26.54693 | 8.210467 | 1.966378 |  | 4.175427 | 2.97E-05 | 0.004309 | CEP112 |
| rna-XM_038165545.1 | 717.9684 | -1.17819 | 0.243705 |  | -4.83451 | 1.33E-06 | 0.000302 | MYH10 |
| rna-XM_038165589.1 | 6.24097 | -6.07855 | 2.486478 |  | -2.44464 | 0.0145 | 0.366395 | ARL16 |
| rna-XM_038165701.1 | 606.2965 | -1.28999 | 0.640918 |  | -2.01272 | 0.044144 | 0.620826 | LOC101804794 |
| rna-XM_038165705.1 | 4.162053 | 5.537514 | 2.721323 |  | 2.034861 | 0.041865 | NA | NSUN5 |
| rna-XM_038165735.1 | 56.80516 | -9.2651 | 3.004412 |  | -3.08383 | 0.002044 | 0.108784 | LOC101800461 |
| rna-XM_038165781.1 | 139.8959 | 7.143115 | 2.606876 |  | 2.740106 | 0.006142 | 0.221996 | NF1 |
| rna-XM_038165796.1 | 98.73861 | 1.158427 | 0.551898 |  | 2.098988 | 0.035818 | 0.568798 | MSI2 |
| rna-XM_038165814.1 | 28.95768 | 8.33533 | 2.016495 |  | 4.133573 | 3.57E-05 | 0.004891 | SYNJ1 |
| rna-XM_038165818.1 | 16.30164 | -7.46395 | 3.624987 |  | -2.05903 | 0.039492 | 0.593752 | SYNJ1 |
| rna-XM_038165888.1 | 25.58449 | 8.156936 | 3.500674 |  | 2.330105 | 0.019801 | 0.432387 | ACACA |
| rna-XM_038165898.1 | 165.4194 | -1.18795 | 0.552706 |  | -2.14934 | 0.031608 | 0.543324 | ACACA |
| rna-XM_038165906.1 | 309.853 | 2.541187 | 0.74781 |  | 3.398173 | 0.000678 | 0.050508 | CLUH |
| rna-XM_038165939.1 | 43.86761 | -8.89216 | 3.064965 |  | -2.90123 | 0.003717 | 0.163137 | GIT1 |
| rna-XM_038165941.1 | 67.41576 | 2.106687 | 1.072761 |  | 1.963798 | 0.049554 | 0.651008 | GIT1 |
| rna-XM_038165996.1 | 1987.153 | 1.088065 | 0.294443 |  | 3.695338 | 0.00022 | 0.020968 | NCOR1 |
| rna-XM_038166000.1 | 17.07231 | 7.574377 | 2.118555 |  | 3.575257 | 0.00035 | 0.030183 | SPECC1 |
| rna-XM_038166016.1 | 102.5262 | 1.554027 | 0.719279 |  | 2.160534 | 0.030731 | 0.53737 | LIME1 |
| rna-XM_038166017.1 | 81.71809 | 2.658021 | 0.906675 |  | 2.931615 | 0.003372 | 0.152453 | TNFRSF6B |
| rna-XM_038166030.1 | 126.1894 | 1.219263 | 0.57054 |  | 2.137034 | 0.032595 | 0.549611 | LOC119713019 |
| rna-XM_038166032.1 | 1613.153 | 1.028323 | 0.280538 |  | 3.665535 | 0.000247 | 0.022901 | PREX1 |
| rna-XM_038166044.1 | 150.6668 | -10.6723 | 2.120502 |  | -5.03291 | 4.83E-07 | 0.000131 | TMEM50B |
| rna-XM_038166084.1 | 149.1494 | 1.622541 | 0.811933 |  | 1.998369 | 0.045677 | 0.628313 | LOC101796159 |
| rna-XM_038166089.1 | 508.9491 | 1.110001 | 0.267321 |  | 4.15231 | 3.29E-05 | 0.004609 | ZBTB46 |
| rna-XM_038166095.1 | 155.0073 | 10.75619 | 2.775353 |  | 3.87561 | 0.000106 | 0.012094 | ZNF512B |
| rna-XM_038166123.1 | 583.9068 | 1.232184 | 0.472676 |  | 2.606823 | 0.009139 | 0.282711 | DNAJC5 |
| rna-XM_038166215.1 | 20.61979 | 7.845771 | 1.798647 |  | 4.36204 | 1.29E-05 | 0.002128 | LOC101802936 |
| rna-XM_038166235.1 | 403.5154 | 2.10845 | 0.73655 |  | 2.862603 | 0.004202 | 0.174278 | ZNF831 |
| rna-XM_038166239.1 | 10.04966 | -6.7667 | 2.21532 |  | -3.0545 | 0.002254 | 0.115724 | RALY |
| rna-XM_038166326.1 | 53.44034 | -1.14414 | 0.50326 |  | -2.27346 | 0.022998 | 0.466748 | HM13 |
| rna-XM_038166372.1 | 146.4502 | -2.01117 | 0.751475 |  | -2.6763 | 0.007444 | 0.251114 | RIPOR3 |
| rna-XM_038166376.1 | 8.757875 | -6.56725 | 2.334982 |  | -2.81255 | 0.004915 | 0.193114 | PEDS1 |
| rna-XM_038166388.1 | 45.09909 | 8.974869 | 2.305033 |  | 3.893596 | 9.88E-05 | 0.011404 | BCAS4 |
| rna-XM_038166425.1 | 126.0671 | -4.86642 | 1.520372 |  | -3.20081 | 0.00137 | 0.084582 | LOC119713377 |
| rna-XM_038166513.1 | 60.42146 | 5.573346 | 2.086622 |  | 2.67099 | 0.007563 | 0.253387 | KCNAB2 |
| rna-XM_038166600.1 | 836.9593 | 3.576271 | 0.652862 |  | 5.477836 | 4.31E-08 | 1.57E-05 | HP1BP3 |
| rna-XM_038166609.1 | 22.73571 | -7.94424 | 1.695451 |  | -4.68562 | 2.79E-06 | 0.000569 | DISP3 |
| rna-XM_038166616.1 | 22.77418 | 7.989371 | 3.438593 |  | 2.323442 | 0.020155 | 0.436934 | TMCO4 |
| rna-XM_038166651.1 | 70.06975 | 1.791211 | 0.671929 |  | 2.665772 | 0.007681 | 0.255492 | ZNF277 |
| rna-XM_038166729.1 | 16.96677 | -7.52171 | 2.08973 |  | -3.59937 | 0.000319 | 0.028033 | CEP104 |
| rna-XM_038166749.1 | 55.71827 | 1.906737 | 0.710949 |  | 2.681961 | 0.007319 | 0.249855 | KIF1B |
| rna-XM_038166777.1 | 30.94027 | 8.431449 | 3.26964 |  | 2.578708 | 0.009917 | 0.298905 | TNFRSF8 |
| rna-XM_038166834.1 | 146.1564 | -3.6466 | 1.017862 |  | -3.58261 | 0.00034 | 0.029618 | LOC101797744 |
| rna-XM_038166850.1 | 84.76864 | 4.299626 | 1.954729 |  | 2.199602 | 0.027835 | 0.513527 | LOC119713470 |
| rna-XM_038166868.1 | 39.46372 | 2.343743 | 1.155162 |  | 2.02893 | 0.042465 | 0.611227 | LOC119713475 |
| rna-XM_038166915.1 | 98.11246 | 2.146286 | 0.565006 |  | 3.798698 | 0.000145 | 0.015179 | RAB11FIP1 |
| rna-XM_038166919.1 | 77.81218 | -6.85818 | 1.167653 |  | -5.87348 | 4.27E-09 | 2.03E-06 | PDLIM2 |
| rna-XM_038166969.1 | 10.45924 | -6.82371 | 2.314789 |  | -2.94788 | 0.0032 | 0.14823 | FAM160B2 |
| rna-XM_038166970.1 | 199.2413 | -1.35372 | 0.641466 |  | -2.11035 | 0.034828 | 0.560548 | FAM160B2 |
| rna-XM_038167006.1 | 313.7605 | 1.643865 | 0.575874 |  | 2.854557 | 0.00431 | 0.176938 | DOCK5 |
| rna-XM_038167024.1 | 9.292094 | 5.709574 | 2.067273 |  | 2.761886 | 0.005747 | 0.213515 | NCAPH |
| rna-XM_038167050.1 | 14.31258 | 7.319503 | 3.575952 |  | 2.046869 | 0.040671 | 0.600533 | TMEM117 |
| rna-XM_038167062.1 | 682.5345 | 2.729378 | 0.81097 |  | 3.365574 | 0.000764 | 0.055016 | EGR3 |
| rna-XM_038167086.1 | 48.93239 | -9.0499 | 3.110202 |  | -2.90975 | 0.003617 | 0.159877 | AAK1 |
| rna-XM_038167109.1 | 22.38459 | -7.92143 | 3.360673 |  | -2.3571 | 0.018419 | 0.417364 | NFU1 |
| rna-XM_038167114.1 | 127.9095 | -1.3951 | 0.697057 |  | -2.00141 | 0.045348 | 0.626311 | POLR3D |
| rna-XM_038167155.1 | 611.2854 | -1.15264 | 0.298875 |  | -3.85659 | 0.000115 | 0.012765 | MAN1C1 |
| rna-XM_038167196.1 | 545.1344 | -1.17668 | 0.545964 |  | -2.15524 | 0.031143 | 0.539815 | AHDC1 |
| rna-XM_038167275.1 | 578.6089 | -1.46447 | 0.41962 |  | -3.48998 | 0.000483 | 0.038404 | CSMD2 |
| rna-XM_038167334.1 | 40.33663 | -3.3783 | 1.379585 |  | -2.44878 | 0.014334 | 0.364176 | TINAGL1 |
| rna-XM_038167360.1 | 873.4891 | 1.15443 | 0.586375 |  | 1.968757 | 0.048981 | 0.647415 | RUNX3 |
| rna-XM_038167415.1 | 10.32939 | -3.53764 | 1.567995 |  | -2.25615 | 0.024061 | 0.476209 | LOC119712987 |
| rna-XM_038167418.1 | 43.59296 | -3.97034 | 1.388069 |  | -2.86033 | 0.004232 | 0.174894 | LOC119713018 |
| rna-XM_038167506.1 | 6.750521 | 6.234746 | 2.385141 |  | 2.613994 | 0.008949 | 0.279611 | CADM1 |
| rna-XM_038167522.1 | 94.82633 | 1.00517 | 0.505871 |  | 1.987008 | 0.046921 | 0.635872 | GRAMD1B |
| rna-XM_038167541.1 | 3014.734 | 1.259429 | 0.331057 |  | 3.804267 | 0.000142 | 0.014956 | APLP2 |
| rna-XM_038167558.1 | 271.7294 | 1.405666 | 0.552077 |  | 2.546142 | 0.010892 | 0.31409 | THY1 |
| rna-XM_038167641.1 | 575.9282 | 1.196193 | 0.403224 |  | 2.966573 | 0.003011 | 0.141785 | ST14 |
| rna-XM_038167652.1 | 63.95175 | 5.53936 | 1.910775 |  | 2.899013 | 0.003743 | 0.163528 | POU2AF1 |
| rna-XM_038167673.1 | 500.8936 | -1.67877 | 0.508777 |  | -3.29962 | 0.000968 | 0.066012 | SC5D |
| rna-XM_038167745.1 | 537.8155 | -1.32769 | 0.458411 |  | -2.8963 | 0.003776 | 0.164377 | LOC119713748 |
| rna-XM_038167815.1 | 122.6327 | 2.124405 | 0.697764 |  | 3.04459 | 0.00233 | 0.118956 | LOC119713782 |
| rna-XM_038167816.1 | 311.0286 | 2.042626 | 0.981924 |  | 2.080227 | 0.037505 | 0.581878 | LOC119713782 |
| rna-XM_038167857.1 | 23.08119 | 2.753692 | 1.376672 |  | 2.000253 | 0.045473 | 0.627113 | LOC113839888 |
| rna-XM_038167861.1 | 6.841845 | -6.21141 | 2.414394 |  | -2.57266 | 0.010092 | 0.301633 | LOC119713071 |
| rna-XM_038167887.1 | 89.99837 | 1.781068 | 0.485833 |  | 3.666012 | 0.000246 | 0.022901 | SHC1 |
| rna-XM_038167918.1 | 21.64507 | 7.915679 | 3.498462 |  | 2.262617 | 0.023659 | 0.472939 | CADM3 |
| rna-XM_038167936.1 | 51.71559 | -1.54825 | 0.437478 |  | -3.53905 | 0.000402 | 0.033265 | PBXIP1 |
| rna-XM_038167957.1 | 73.14234 | -2.03541 | 1.032042 |  | -1.97222 | 0.048585 | 0.645587 | LOC119713830 |
| rna-XM_038167991.1 | 22.85245 | -7.95125 | 3.342199 |  | -2.37905 | 0.017358 | 0.402062 | SEMA6C |
| rna-XM_038168101.1 | 373.717 | 1.058361 | 0.32506 |  | 3.255899 | 0.00113 | 0.073384 | UBAP2L |
| rna-XM_038168133.1 | 31.94384 | 8.477431 | 3.185644 |  | 2.661136 | 0.007788 | 0.256347 | TPM3 |
| rna-XM_038168202.1 | 118.3455 | -10.324 | 2.802542 |  | -3.68379 | 0.00023 | 0.021665 | PLXNA2 |
| rna-XM_038168244.1 | 39.16377 | 8.771761 | 1.48545 |  | 5.90512 | 3.52E-09 | 1.81E-06 | USP49 |
| rna-XM_038168245.1 | 13.74506 | 7.260617 | 3.645476 |  | 1.991679 | 0.046406 | 0.631857 | USP49 |
| rna-XM_038168273.1 | 8.466065 | -4.06523 | 1.914702 |  | -2.12317 | 0.03374 | 0.554394 | SYCP1 |
| rna-XM_038168293.1 | 24.14763 | 8.073693 | 3.312525 |  | 2.437323 | 0.014796 | 0.369315 | LAMB3 |
| rna-XM_038168295.1 | 25.00803 | 2.221232 | 1.072805 |  | 2.07049 | 0.038406 | 0.588926 | CAMK1G |
| rna-XM_038168387.1 | 21.70494 | -7.87691 | 3.370718 |  | -2.33686 | 0.019446 | 0.428239 | SMIM29 |
| rna-XM_038168411.1 | 57.36686 | 3.9993 | 1.967961 |  | 2.032205 | 0.042133 | 0.608722 | DUS4L |
| rna-XM_038168427.1 | 18.32648 | -7.63348 | 2.108879 |  | -3.61969 | 0.000295 | 0.026228 | LOC101794319 |
| rna-XM_038168462.1 | 645.5332 | 1.342652 | 0.614732 |  | 2.184127 | 0.028953 | 0.526385 | PTPN7 |
| rna-XM_038168480.1 | 25.61133 | -8.11573 | 3.32586 |  | -2.44019 | 0.01468 | 0.36788 | C27H6orf89 |
| rna-XM_038168484.1 | 201.1408 | 1.019106 | 0.455542 |  | 2.237127 | 0.025278 | 0.490972 | ZC3H11A |
| rna-XM_038168541.1 | 11.89307 | -7.00974 | 2.218722 |  | -3.15936 | 0.001581 | 0.093131 | WARS2 |
| rna-XM_038168566.1 | 149.2109 | -1.22201 | 0.513644 |  | -2.37909 | 0.017355 | 0.402062 | LOC119714007 |
| rna-XM_038168680.1 | 458.1438 | -3.13709 | 1.383181 |  | -2.26802 | 0.023328 | 0.469958 | NKIRAS2 |
| rna-XM_038168747.1 | 140.7065 | -2.60474 | 1.075035 |  | -2.42294 | 0.015396 | 0.37612 | GJC1 |
| rna-XM_038168756.1 | 84.45225 | -9.83721 | 2.864909 |  | -3.43369 | 0.000595 | 0.045766 | MPP3 |
| rna-XM_038168761.1 | 19.86698 | 7.792423 | 3.413617 |  | 2.282747 | 0.022445 | 0.462526 | MPP3 |
| rna-XM_038168797.1 | 17.926 | -7.60125 | 3.55003 |  | -2.14118 | 0.03226 | 0.547507 | MAPT |
| rna-XM_038168807.1 | 1040.921 | -1.14161 | 0.320749 |  | -3.55921 | 0.000372 | 0.031651 | LOC101792081 |
| rna-XM_038168813.1 | 1102.298 | 1.306651 | 0.502943 |  | 2.598013 | 0.009377 | 0.288129 | PPP1R9B |
| rna-XM_038168817.1 | 726.9674 | -1.8397 | 0.642664 |  | -2.86261 | 0.004202 | 0.174278 | ZNF652 |
| rna-XM_038168828.1 | 33.50836 | 8.546641 | 3.150823 |  | 2.71251 | 0.006678 | 0.235772 | TOP2A |
| rna-XM_038168830.1 | 5.080252 | 5.825676 | 2.684646 |  | 2.169997 | 0.030007 | NA | LOC119713025 |
| rna-XM_038168909.1 | 26.36882 | 1.939609 | 0.859106 |  | 2.257706 | 0.023964 | 0.475225 | LOC101804340 |
| rna-XM_038168971.1 | 682.4379 | 4.220841 | 1.435809 |  | 2.939696 | 0.003285 | 0.150344 | MUC16 |
| rna-XM_038168993.1 | 130.2958 | -10.4627 | 2.788063 |  | -3.75268 | 0.000175 | 0.017648 | DAZAP1 |
| rna-XM_038169073.1 | 18.59105 | 7.696509 | 3.437226 |  | 2.239163 | 0.025145 | 0.489443 | SUGP1 |
| rna-XM_038169076.1 | 32.08328 | -8.44091 | 3.199642 |  | -2.63808 | 0.008338 | 0.267519 | MYO9B |
| rna-XM_038169081.1 | 209.8382 | -1.01773 | 0.344026 |  | -2.95829 | 0.003094 | 0.144743 | TBXA2R |
| rna-XM_038169182.1 | 283.6811 | 1.831856 | 0.69618 |  | 2.631297 | 0.008506 | 0.271186 | LOC101794304 |
| rna-XM_038169184.1 | 44.4647 | -1.83855 | 0.671397 |  | -2.73839 | 0.006174 | 0.222527 | FKBP8 |
| rna-XM_038169192.1 | 189.5959 | 2.715426 | 1.110401 |  | 2.445446 | 0.014467 | 0.366337 | LOC101794304 |
| rna-XM_038169196.1 | 142.3858 | -1.20795 | 0.606447 |  | -1.99185 | 0.046388 | 0.631857 | PHEX |
| rna-XM_038169259.1 | 133.0869 | 1.309879 | 0.496309 |  | 2.63924 | 0.008309 | 0.267413 | CSNK1G2 |
| rna-XM_038169262.1 | 51.22494 | 9.158977 | 3.017577 |  | 3.035209 | 0.002404 | 0.121726 | CSNK1G2 |
| rna-XM_038169293.1 | 44.80444 | -4.11714 | 2.01053 |  | -2.04779 | 0.040581 | 0.599996 | LOC101803211 |
| rna-XM_038169308.1 | 20.20009 | -7.77335 | 1.766035 |  | -4.40159 | 1.07E-05 | 0.001825 | TPM4 |
| rna-XM_038169314.1 | 90.6398 | 1.462097 | 0.668704 |  | 2.186465 | 0.028782 | 0.524287 | LOC101803519 |
| rna-XM_038169345.1 | 113.323 | 2.884601 | 0.783985 |  | 3.679409 | 0.000234 | 0.021931 | CD22 |
| rna-XM_038169510.1 | 9.77768 | -2.85259 | 1.205064 |  | -2.36717 | 0.017925 | 0.411899 | LOC119712996 |
| rna-XM_038169697.1 | 279.8826 | -4.07401 | 1.974262 |  | -2.06356 | 0.039059 | 0.591879 | B4GALNT3 |
| rna-XM_038169746.1 | 7209.025 | 1.02394 | 0.23603 |  | 4.338176 | 1.44E-05 | 0.002294 | SMARCA2 |
| rna-XM_038169753.1 | 21.23552 | -7.84541 | 1.805498 |  | -4.34529 | 1.39E-05 | 0.00224 | SETBP1 |
| rna-XM_038169760.1 | 102.174 | -6.94883 | 1.804379 |  | -3.85109 | 0.000118 | 0.012978 | APC |
| rna-XM_038169762.1 | 280.8637 | 11.61367 | 3.906918 |  | 2.972591 | 0.002953 | 0.13956 | APC |
| rna-XM_038169795.1 | 16.75671 | 7.546676 | 1.811762 |  | 4.165379 | 3.11E-05 | 0.004418 | RUSC2 |
| rna-XM_038169838.1 | 20.93717 | 2.109283 | 1.035081 |  | 2.037796 | 0.04157 | 0.605287 | FAM169A |
| rna-XM_038169858.1 | 14.73722 | 7.361405 | 3.570127 |  | 2.061945 | 0.039213 | 0.592617 | RMI1 |
| rna-XM_038169873.1 | 162.3318 | -1.52324 | 0.495642 |  | -3.07327 | 0.002117 | 0.110666 | PSD3 |
| rna-XM_038169922.1 | 62.48209 | 1.634936 | 0.692701 |  | 2.360231 | 0.018264 | 0.415202 | CTIF |
| rna-XM_038169968.1 | 133.8565 | -10.5016 | 2.812819 |  | -3.73349 | 0.000189 | 0.018492 | ZNF462 |
| rna-XM_038169970.1 | 463.7305 | -3.58543 | 1.724793 |  | -2.07876 | 0.03764 | 0.582926 | ZNF462 |
| rna-XM_038169980.1 | 23.40355 | 8.02913 | 1.713 |  | 4.687175 | 2.77E-06 | 0.000568 | SSBP2 |
| rna-XM_038169984.1 | 23.38891 | -7.98475 | 3.337022 |  | -2.39278 | 0.016721 | 0.396131 | SSBP2 |
| rna-XM_038169993.1 | 36.4623 | -8.62541 | 3.131289 |  | -2.75459 | 0.005877 | 0.216407 | ERBIN |
| rna-XM_038170029.1 | 15.45099 | -7.38687 | 3.552089 |  | -2.07958 | 0.037564 | 0.582228 | CAST |
| rna-XM_038170056.1 | 16.0747 | 7.486854 | 3.53265 |  | 2.119331 | 0.034062 | 0.555979 | MIER3 |
| rna-XM_038170083.1 | 27.98523 | -8.24372 | 3.254141 |  | -2.5333 | 0.011299 | 0.319391 | MAST4 |
| rna-XM_038170084.1 | 40.29345 | 8.812467 | 3.110584 |  | 2.833059 | 0.00461 | 0.184641 | MAST4 |
| rna-XM_038170105.1 | 13.66871 | -7.2099 | 1.928511 |  | -3.73858 | 0.000185 | 0.018227 | NRG1 |
| rna-XM_038170130.1 | 192.9952 | -2.15125 | 0.690501 |  | -3.11549 | 0.001836 | 0.101637 | ACO1 |
| rna-XM_038170134.1 | 869.9214 | 1.31649 | 0.592234 |  | 2.222923 | 0.026221 | 0.499464 | PALM2AKAP2 |
| rna-XM_038170147.1 | 19.61337 | 1.674012 | 0.749693 |  | 2.232932 | 0.025553 | 0.49327 | SEMA4D |
| rna-XM_038170149.1 | 18.43564 | -7.64134 | 2.580338 |  | -2.96137 | 0.003063 | 0.143661 | ZNF131 |
| rna-XM_038170163.1 | 34.07661 | 5.145606 | 1.783337 |  | 2.885381 | 0.003909 | 0.168747 | FER |
| rna-XM_038170172.1 | 67.06055 | -1.45351 | 0.724706 |  | -2.00566 | 0.044893 | 0.623498 | CDK7 |
| rna-XM_038170198.1 | 5.382607 | -5.86528 | 2.530015 |  | -2.31828 | 0.020434 | NA | ARL15 |
| rna-XM_038170215.1 | 4.917922 | 5.777556 | 2.646822 |  | 2.182827 | 0.029049 | NA | FSD1L |
| rna-XM_038170313.1 | 82.62666 | 1.223949 | 0.496678 |  | 2.464268 | 0.013729 | 0.357525 | MSH3 |
| rna-XM_038170332.1 | 598.5513 | 1.211811 | 0.583794 |  | 2.07575 | 0.037917 | 0.584328 | MAP3K1 |
| rna-XM_038170337.1 | 1158.407 | 1.314757 | 0.356056 |  | 3.692553 | 0.000222 | 0.021008 | MAP3K1 |
| rna-XM_038170368.1 | 700.231 | -1.3932 | 0.6362 |  | -2.18987 | 0.028533 | 0.521792 | FREM1 |
| rna-XM_038170372.1 | 276.6053 | -1.06774 | 0.442033 |  | -2.41551 | 0.015713 | 0.380903 | FREM1 |
| rna-XM_038170392.1 | 67.44922 | 3.394724 | 1.088876 |  | 3.117641 | 0.001823 | 0.101198 | SPEF2 |
| rna-XM_038170393.1 | 345.3318 | -1.23171 | 0.28132 |  | -4.37831 | 1.20E-05 | 0.002002 | DYM |
| rna-XM_038170419.1 | 8.224519 | 3.976209 | 1.987488 |  | 2.00062 | 0.045433 | 0.627027 | CDC20B |
| rna-XM_038170429.1 | 41.13997 | 8.842684 | 3.07565 |  | 2.875062 | 0.004039 | 0.171667 | SMAD2 |
| rna-XM_038170430.1 | 324.3776 | -11.7787 | 1.28568 |  | -9.16149 | 5.12E-20 | 1.60E-16 | SMAD2 |
| rna-XM_038170507.1 | 15.28054 | -7.37057 | 3.621724 |  | -2.0351 | 0.041841 | 0.60691 | ADAMTS6 |
| rna-XM_038170515.1 | 18.90776 | -7.67784 | 3.453921 |  | -2.22294 | 0.02622 | 0.499464 | KDM4C |
| rna-XM_038170700.1 | 7.52057 | 6.390349 | 2.530532 |  | 2.525299 | 0.01156 | 0.323269 | LOC119714447 |
| rna-XM_038170702.1 | 92.3178 | 3.19752 | 1.342623 |  | 2.381547 | 0.01724 | 0.400579 | CD72 |
| rna-XM_038170705.1 | 97.5632 | 2.779115 | 1.179817 |  | 2.355549 | 0.018495 | 0.418229 | LOC119714450 |
| rna-XM_038170740.1 | 103.8387 | 1.258756 | 0.542901 |  | 2.318575 | 0.020418 | 0.439085 | IL7R |
| rna-XM_038170750.1 | 180.3722 | -1.03604 | 0.477271 |  | -2.17076 | 0.029949 | 0.531708 | ENC1 |
| rna-XM_038170763.1 | 15.86236 | 7.467219 | 3.588245 |  | 2.081023 | 0.037432 | 0.581878 | SYTL5 |
| rna-XM_038170846.1 | 15.16649 | -7.35975 | 3.56321 |  | -2.06548 | 0.038877 | 0.591115 | ELAVL2 |
| rna-XM_038170962.1 | 91.46627 | -1.29661 | 0.573851 |  | -2.25948 | 0.023854 | 0.474354 | GKAP1 |
| rna-XM_038170967.1 | 136.098 | 1.926098 | 0.427072 |  | 4.510013 | 6.48E-06 | 0.001175 | MLLT3 |
| rna-XM_038170982.1 | 146.7498 | 5.138675 | 1.813227 |  | 2.833995 | 0.004597 | 0.184495 | CD274 |
| rna-XM_038170998.1 | 23.52349 | 8.035755 | 3.494933 |  | 2.299258 | 0.02149 | 0.449762 | PAX5 |
| rna-XM_038171000.1 | 685.9928 | 3.714582 | 1.024423 |  | 3.626023 | 0.000288 | 0.025777 | PAX5 |
| rna-XM_038171036.1 | 6483.505 | 1.712788 | 0.863451 |  | 1.983653 | 0.047294 | 0.637552 | LOC119714498 |
| rna-XM_038171050.1 | 1200.048 | 2.416625 | 0.963678 |  | 2.50771 | 0.012152 | 0.332596 | C7 |
| rna-XM_038171051.1 | 347.1981 | 2.193016 | 1.083639 |  | 2.023752 | 0.042996 | 0.614633 | C7 |
| rna-XM_038171118.1 | 14.40371 | 7.328746 | 3.593028 |  | 2.039713 | 0.041379 | 0.604446 | LOC119714521 |
| rna-XM_038171136.1 | 6.892198 | 6.265652 | 2.451535 |  | 2.555808 | 0.010594 | 0.309729 | PTPRD |
| rna-XM_038171221.1 | 35.5318 | -8.58822 | 1.499064 |  | -5.72906 | 1.01E-08 | 4.36E-06 | FAM214B |
| rna-XM_038171222.1 | 30.84841 | 8.427095 | 3.198542 |  | 2.634667 | 0.008422 | 0.269424 | FAM214B |
| rna-XM_038171240.1 | 30.51004 | -2.10796 | 0.962965 |  | -2.18903 | 0.028595 | 0.522658 | NMRK1 |
| rna-XM_038171250.1 | 47.3446 | -4.07237 | 1.838435 |  | -2.21513 | 0.026751 | 0.503525 | TRPM6 |
| rna-XM_038171278.1 | 91.00198 | 9.987882 | 2.929737 |  | 3.409139 | 0.000652 | 0.049007 | BNC2 |
| rna-XM_038171390.1 | 12.47562 | -7.0787 | 2.348885 |  | -3.01364 | 0.002581 | 0.12762 | GIN1 |
| rna-XM_038171399.1 | 282.5802 | 1.144539 | 0.522077 |  | 2.192278 | 0.028359 | 0.519623 | TCF4 |
| rna-XM_038171425.1 | 16.92652 | 7.561521 | 3.473962 |  | 2.176628 | 0.029508 | 0.528936 | PIP5K1B |
| rna-XM_038171485.1 | 20.20364 | -7.7735 | 3.407744 |  | -2.28113 | 0.022541 | 0.463469 | IQGAP2 |
| rna-XM_038171542.1 | 59.15224 | -9.32351 | 2.956352 |  | -3.15372 | 0.001612 | 0.094371 | SMARCC2 |
| rna-XM_038171556.1 | 13.78019 | 7.264426 | 1.927048 |  | 3.769718 | 0.000163 | 0.01662 | GLI1 |
| rna-XM_038171598.1 | 14.44446 | -7.28961 | 1.916225 |  | -3.80415 | 0.000142 | 0.014956 | GALNT6 |
| rna-XM_038171637.1 | 19.56333 | -7.727 | 2.434077 |  | -3.17451 | 0.001501 | 0.089554 | LOC119714666 |
| rna-XM_038171686.1 | 184.1316 | 4.542024 | 1.863285 |  | 2.437643 | 0.014783 | 0.369315 | ORMDL2 |
| rna-XM_038171773.1 | 37.93 | 1.341299 | 0.668241 |  | 2.007208 | 0.044727 | 0.623412 | LOC113841160 |
| rna-XM_038171885.1 | 5.550093 | 5.951493 | 2.732595 |  | 2.177964 | 0.029409 | 0.528936 | GPAT2 |
| rna-XM_038171900.1 | 343.5006 | 1.267729 | 0.624798 |  | 2.029022 | 0.042456 | 0.611227 | LOC119714837 |
| rna-XM_038171910.1 | 872.4713 | 1.281255 | 0.563386 |  | 2.274205 | 0.022954 | 0.466344 | LOC101804201 |
| rna-XM_038171927.1 | 77.51712 | -7.27032 | 1.31897 |  | -5.51212 | 3.55E-08 | 1.33E-05 | DNMT1 |
| rna-XM_038172027.1 | 7.928431 | -6.42379 | 2.589094 |  | -2.4811 | 0.013098 | 0.347475 | LOC113841504 |
| rna-XM_038172129.1 | 9496.599 | 3.719964 | 1.878786 |  | 1.979982 | 0.047706 | 0.63979 | LOC119715006 |
| rna-XM_038172161.1 | 613.2906 | 1.540981 | 0.664242 |  | 2.319908 | 0.020346 | 0.439032 | LOC119715020 |
| rna-XM_038172167.1 | 182.6869 | 3.104908 | 0.559717 |  | 5.547285 | 2.90E-08 | 1.10E-05 | LOC119715026 |
| rna-XM_038172253.1 | 56.19662 | -1.70871 | 0.833483 |  | -2.05009 | 0.040356 | 0.597847 | COL2A1 |
| rna-XM_038172262.1 | 265.8598 | -1.66499 | 0.550053 |  | -3.02695 | 0.00247 | 0.124262 | LOC119715087 |
| rna-XM_038172265.1 | 96.38213 | -2.19361 | 1.060718 |  | -2.06805 | 0.038636 | 0.590313 | LOC119715087 |
| rna-XM_038172289.1 | 24.21605 | -8.03472 | 2.000548 |  | -4.01626 | 5.91E-05 | 0.007496 | STX5 |
| rna-XM_038172299.1 | 114.4242 | 3.818535 | 1.350865 |  | 2.826732 | 0.004703 | 0.186734 | AGPAT3 |
| rna-XM_038172350.1 | 6.573681 | -5.15818 | 2.026049 |  | -2.54593 | 0.010899 | 0.31409 | LOC119715161 |
| rna-XM_038172440.1 | 9.997811 | -6.75917 | 2.579032 |  | -2.62082 | 0.008772 | 0.276379 | CASC3 |
| rna-XM_038172550.1 | 42.28344 | 8.882068 | 3.158719 |  | 2.811921 | 0.004925 | 0.193114 | LOC101794328 |
| rna-XM_038172554.1 | 265.4613 | -11.4893 | 3.906928 |  | -2.94075 | 0.003274 | 0.150145 | HDAC7 |
| rna-XM_038172567.1 | 193.5537 | -1.18005 | 0.420386 |  | -2.80707 | 0.004999 | 0.194315 | TESPA1 |
| rna-XM_038172674.1 | 52.12133 | -1.34748 | 0.657016 |  | -2.05091 | 0.040276 | 0.597135 | LOC119713029 |
| rna-XM_038172687.1 | 477.3874 | -1.16307 | 0.436109 |  | -2.66691 | 0.007655 | 0.255492 | LOC119715380 |
| rna-XM_038172705.1 | 1016.285 | -1.29497 | 0.368408 |  | -3.51505 | 0.00044 | 0.035634 | LOC119715394 |
| rna-XM_038172710.1 | 249.8605 | -2.05454 | 0.795513 |  | -2.58266 | 0.009804 | 0.296219 | LOC119715397 |
| rna-XM_038172755.1 | 439.0194 | -1.43962 | 0.574748 |  | -2.50479 | 0.012253 | 0.332941 | LOC119715450 |
| rna-XM_038172846.1 | 7.351353 | 6.358142 | 2.489473 |  | 2.554011 | 0.010649 | 0.310492 | LOC113840883 |
| rna-XM_038172874.1 | 143004.2 | -1.98961 | 0.704828 |  | -2.82283 | 0.00476 | 0.188225 | LOC113841989 |
| rna-XM_038172888.1 | 305.3563 | 3.641732 | 1.269693 |  | 2.868199 | 0.004128 | 0.173277 | FOSB |
| rna-XM_038172912.1 | 80.54419 | -1.89492 | 0.776183 |  | -2.44133 | 0.014633 | 0.36788 | SH3KBP1 |
| rna-XM_038172969.1 | 18.97809 | 7.72609 | 3.458418 |  | 2.233995 | 0.025483 | 0.492666 | GPM6B |
| rna-XM_038173083.1 | 12.43898 | 3.81146 | 1.814636 |  | 2.100399 | 0.035694 | 0.567545 | CFAP54 |
| rna-XM_038173154.1 | 72.00552 | -6.01678 | 2.607253 |  | -2.30771 | 0.021015 | 0.445651 | ENOX1 |
| rna-XM_038173170.1 | 605.1138 | 2.481366 | 0.669425 |  | 3.70671 | 0.00021 | 0.020203 | SLC9A7 |
| rna-XM_038173212.1 | 92.59641 | 1.281706 | 0.540582 |  | 2.370976 | 0.017741 | 0.408889 | REV1 |
| rna-XM_038173215.1 | 25.85136 | -7.15696 | 2.23535 |  | -3.20172 | 0.001366 | 0.084454 | REV1 |
| rna-XM_038173225.1 | 25.75021 | -8.12366 | 3.274393 |  | -2.48097 | 0.013103 | 0.347475 | KIAA0930 |
| rna-XM_038173304.1 | 1324.858 | 1.183128 | 0.575925 |  | 2.054311 | 0.039946 | 0.595286 | LOC101793940 |
| rna-XM_038173333.1 | 828.7141 | -1.26597 | 0.463988 |  | -2.72845 | 0.006363 | 0.227844 | LOC101792784 |
| rna-XM_038173362.1 | 1606.126 | 12.33233 | 3.586842 |  | 3.438214 | 0.000586 | 0.045354 | LOC119715617 |
| rna-XM_038173390.1 | 120.6181 | 1.864899 | 0.804455 |  | 2.318214 | 0.020438 | 0.439085 | PLXNB2 |
| rna-XM_038173394.1 | 351.2689 | 1.4818 | 0.449463 |  | 3.296822 | 0.000978 | 0.06618 | LOC119715627 |
| rna-XM_038173455.1 | 2646.245 | 1.496885 | 0.592021 |  | 2.528432 | 0.011457 | 0.322573 | FARP1 |
| rna-XM_038173484.1 | 410.8363 | 12.16274 | 1.247343 |  | 9.750912 | 1.83E-22 | 1.14E-18 | PDE3A |
| rna-XM_038173487.1 | 167.1336 | 10.86489 | 2.71889 |  | 3.996075 | 6.44E-05 | 0.008082 | PDE3A |
| rna-XM_038173508.1 | 13.11678 | 7.193597 | 3.652404 |  | 1.969551 | 0.04889 | 0.647097 | PPFIBP1 |
| rna-XM_038173518.1 | 131.8084 | -4.12095 | 1.904937 |  | -2.1633 | 0.030518 | 0.534889 | ARNTL2 |
| rna-XM_038173607.1 | 11.14973 | -6.91618 | 2.253393 |  | -3.06923 | 0.002146 | 0.111541 | RAD54B |
| rna-XM_038173687.1 | 6.5362 | 6.1884 | 2.452674 |  | 2.523124 | 0.011632 | 0.32428 | LOC101790251 |
| rna-XM_038173754.1 | 475.4913 | -1.53984 | 0.509465 |  | -3.02247 | 0.002507 | 0.125541 | GRINA |
| rna-XM_038173830.1 | 33.2578 | -8.49263 | 3.172785 |  | -2.67671 | 0.007435 | 0.251114 | BICD1 |
| rna-XM_038173872.1 | 33.21829 | 1.852295 | 0.88165 |  | 2.100942 | 0.035646 | 0.567027 | HDAC9 |
| rna-XM_038173882.1 | 261.5547 | -1.22306 | 0.52702 |  | -2.32071 | 0.020302 | 0.438345 | ZNF521 |
| rna-XM_038173898.1 | 581.3009 | -12.6203 | 1.531991 |  | -8.23784 | 1.75E-16 | 3.66E-13 | MAP4 |
| rna-XM_038173899.1 | 157.8743 | -10.7398 | 1.545766 |  | -6.94788 | 3.71E-12 | 3.31E-09 | MAP4 |
| rna-XM_038173946.1 | 25.41291 | -8.10464 | 3.279267 |  | -2.47148 | 0.013456 | 0.352597 | TRAPPC9 |
| rna-XM_038173986.1 | 470.8401 | -3.74574 | 0.860011 |  | -4.35545 | 1.33E-05 | 0.002167 | PTPRM |
| rna-XM_038173987.1 | 112.2185 | 8.479305 | 2.471393 |  | 3.430981 | 0.000601 | 0.046041 | NFATC1 |
| rna-XM_038174001.1 | 20.83019 | 7.861086 | 1.995176 |  | 3.940046 | 8.15E-05 | 0.009736 | GAREM1 |
| rna-XM_038174072.1 | 51.18508 | 9.157588 | 3.020009 |  | 3.032305 | 0.002427 | 0.122572 | FAM126A |
| rna-XM_038174088.1 | 84.45218 | 3.384867 | 1.196912 |  | 2.827999 | 0.004684 | 0.186391 | LOC101801689 |
| rna-XM_038174091.1 | 45.55816 | 4.231921 | 1.902218 |  | 2.22473 | 0.026099 | 0.498865 | DNAH5 |
| rna-XM_038174304.1 | 1527.337 | -3.6257 | 0.716588 |  | -5.05967 | 4.20E-07 | 0.000116 | TGFBR2 |
| rna-XM_038174316.1 | 83.6231 | -1.02585 | 0.430521 |  | -2.38282 | 0.017181 | 0.40049 | ARHGAP12 |
| rna-XM_038174324.1 | 273.9689 | -1.11862 | 0.48239 |  | -2.3189 | 0.0204 | 0.439085 | PCMTD1 |
| rna-XM_038174384.1 | 92.78876 | -4.1953 | 1.389386 |  | -3.01953 | 0.002532 | 0.126117 | SUPT20H |
| rna-XM_038174428.1 | 153.4885 | -1.18918 | 0.526602 |  | -2.25822 | 0.023932 | 0.475225 | LOC101800567 |
| rna-XM_038174433.1 | 50.08882 | -9.08339 | 1.594221 |  | -5.6977 | 1.21E-08 | 5.18E-06 | THNSL1 |
| rna-XM_038174548.1 | 26.07336 | -2.03998 | 0.992935 |  | -2.0545 | 0.039928 | 0.595286 | TMEM65 |
| rna-XM_038174577.1 | 60.8629 | 2.481461 | 1.072552 |  | 2.313604 | 0.020689 | 0.442208 | NUDCD1 |
| rna-XM_038174580.1 | 30.54923 | 8.413065 | 1.622789 |  | 5.184324 | 2.17E-07 | 6.61E-05 | NBEA |
| rna-XM_038174583.1 | 63.93713 | -1.58038 | 0.70027 |  | -2.25681 | 0.02402 | 0.475643 | THRB |
| rna-XM_038174586.1 | 56.11117 | 9.290242 | 2.975674 |  | 3.122063 | 0.001796 | 0.100135 | THRB |
| rna-XM_038174625.1 | 682.9489 | -12.8528 | 1.208639 |  | -10.6341 | 2.07E-26 | 2.59E-22 | STARD13 |
| rna-XM_038174643.1 | 786.5384 | 1.102056 | 0.419974 |  | 2.624109 | 0.008688 | 0.274981 | STARD13 |
| rna-XM_038174675.1 | 333.26 | 1.375663 | 0.644412 |  | 2.134758 | 0.032781 | 0.549611 | MROH1 |
| rna-XM_038174695.1 | 28.30978 | -8.26033 | 3.28377 |  | -2.5155 | 0.011886 | 0.327726 | DUSP22 |
| rna-XM_038174696.1 | 50.35113 | 4.368481 | 1.934871 |  | 2.257763 | 0.02396 | 0.475225 | N4BP2L2 |
| rna-XM_038174743.1 | 14.51915 | -7.29687 | 3.651569 |  | -1.99828 | 0.045686 | 0.628313 | RNF152 |
| rna-XM_038174870.1 | 26.84766 | -8.18392 | 3.259306 |  | -2.51094 | 0.012041 | 0.330953 | TCAIM |
| rna-XM_038174882.1 | 15.66433 | -7.40646 | 3.542487 |  | -2.09075 | 0.03655 | 0.574174 | SLC46A3 |
| rna-XM_038174913.1 | 261.6378 | -8.38318 | 3.504668 |  | -2.392 | 0.016757 | 0.396214 | PAN3 |
| rna-XM_038175037.1 | 81.85538 | -9.79216 | 2.928005 |  | -3.34431 | 0.000825 | 0.058506 | CRISPLD1 |
| rna-XM_038175084.1 | 824.6139 | 1.113054 | 0.487822 |  | 2.281682 | 0.022508 | 0.463312 | MYC |
| rna-XM_038175094.1 | 32.71756 | -1.59419 | 0.751981 |  | -2.11999 | 0.034007 | 0.555556 | LOC101792542 |
| rna-XM_038175112.1 | 27.33974 | -8.20991 | 3.34392 |  | -2.45518 | 0.014082 | 0.361769 | LOC113841369 |
| rna-XM_038175116.1 | 49.75207 | 3.656076 | 1.391574 |  | 2.627295 | 0.008607 | 0.27309 | LOC113841369 |
| rna-XM_038175155.1 | 18.88503 | -7.67616 | 3.454139 |  | -2.22231 | 0.026263 | 0.500001 | NCAPG2 |
| rna-XM_038175239.1 | 120.2934 | 9.426787 | 1.322559 |  | 7.127686 | 1.02E-12 | 1.06E-09 | SCRIB |
| rna-XM_038175254.1 | 18.5328 | -7.64908 | 3.442312 |  | -2.22208 | 0.026278 | 0.500044 | SCRIB |
| rna-XM_038175291.1 | 324.2339 | 2.178697 | 0.901044 |  | 2.41797 | 0.015607 | 0.379317 | IRF4 |
| rna-XM_038175327.1 | 20.71095 | -7.80974 | 1.829226 |  | -4.26942 | 1.96E-05 | 0.003039 | NETO1 |
| rna-XM_038175332.1 | 7.881804 | 6.458469 | 2.270259 |  | 2.844816 | 0.004444 | 0.180559 | NETO1 |
| rna-XM_038175391.1 | 25.6099 | 1.859305 | 0.876597 |  | 2.121049 | 0.033918 | 0.555289 | HEPACAM2 |
| rna-XM_038175484.1 | 42.99216 | 8.906205 | 3.322898 |  | 2.680252 | 0.007357 | 0.250224 | ROCK1 |
| rna-XM_038175508.1 | 648.4573 | 1.174157 | 0.480631 |  | 2.442949 | 0.014568 | 0.367263 | RSU1 |
| rna-XM_038175562.1 | 261.867 | -1.28208 | 0.348844 |  | -3.67522 | 0.000238 | 0.022239 | FAM8A1 |
| rna-XM_038175606.1 | 11.01308 | 3.966348 | 1.706534 |  | 2.324213 | 0.020114 | 0.436289 | PP2D1 |
| rna-XM_038175636.1 | 99.37421 | 10.11481 | 2.827344 |  | 3.577496 | 0.000347 | 0.029994 | EXPH5 |
| rna-XM_038175648.1 | 23.93949 | -8.01842 | 2.009087 |  | -3.99108 | 6.58E-05 | 0.008186 | OTULIN |
| rna-XM_038175675.1 | 39.86589 | -8.75424 | 3.126977 |  | -2.79959 | 0.005117 | 0.197336 | BTD |
| rna-XM_038175686.1 | 14.63522 | -7.30825 | 3.672197 |  | -1.99016 | 0.046574 | 0.632985 | GREB1L |
| rna-XM_038175691.1 | 13.43723 | 7.228419 | 3.635727 |  | 1.988163 | 0.046794 | 0.634827 | ESCO1 |
| rna-XM_038175838.1 | 20.39722 | -7.78747 | 3.383529 |  | -2.30158 | 0.021359 | 0.448759 | UTRN |
| rna-XM_038175842.1 | 524.3794 | 1.265707 | 0.618274 |  | 2.047161 | 0.040642 | 0.600533 | UTRN |
| rna-XM_038175844.1 | 29.13746 | 8.344523 | 1.633096 |  | 5.109635 | 3.23E-07 | 9.32E-05 | TMEM181 |
| rna-XM_038175980.1 | 66.25119 | -7.06973 | 2.236327 |  | -3.16131 | 0.001571 | 0.092813 | NRXN1 |
| rna-XM_038176021.1 | 52.29305 | 1.408851 | 0.658307 |  | 2.140111 | 0.032346 | 0.547979 | EHBP1 |
| rna-XM_038176042.1 | 6.061844 | 6.07954 | 2.457015 |  | 2.47436 | 0.013348 | 0.350746 | EML4 |
| rna-XM_038176129.1 | 51.11404 | 1.012443 | 0.487575 |  | 2.076487 | 0.037849 | 0.583757 | PCNX2 |
| rna-XM_038176179.1 | 84.83124 | 9.886502 | 2.896409 |  | 3.413366 | 0.000642 | 0.048447 | MARK1 |
| rna-XM_038176186.1 | 137.1792 | 1.677154 | 0.666662 |  | 2.515747 | 0.011878 | 0.327726 | SENP6 |
| rna-XM_038176195.1 | 229.2509 | 1.864827 | 0.699362 |  | 2.66647 | 0.007665 | 0.255492 | QKI |
| rna-XM_038176222.1 | 17.05797 | -5.11489 | 1.828027 |  | -2.79804 | 0.005141 | 0.197777 | RUNX2 |
| rna-XM_038176339.1 | 246.4636 | -2.5518 | 1.058163 |  | -2.41154 | 0.015885 | 0.383589 | LOC119716357 |
| rna-XM_038176343.1 | 59.45672 | -9.33077 | 1.40701 |  | -6.63163 | 3.32E-11 | 2.40E-08 | SSTR4 |
| rna-XM_038176344.1 | 393.4809 | 12.10021 | 1.217381 |  | 9.939541 | 2.80E-23 | 2.10E-19 | SSTR4 |
| rna-XM_038176393.1 | 18.92323 | 4.191463 | 2.071039 |  | 2.023845 | 0.042986 | 0.614633 | FNDC4 |
| rna-XM_038176421.1 | 676.4091 | 1.062372 | 0.442427 |  | 2.401233 | 0.01634 | 0.390048 | FOSL2 |
| rna-XM_038176425.1 | 26.53926 | 4.045011 | 1.962794 |  | 2.060843 | 0.039318 | 0.592771 | GEN1 |
| rna-XM_038176432.1 | 20.26724 | 2.662385 | 1.252948 |  | 2.124897 | 0.033595 | 0.554394 | LOC113842317 |
| rna-XM_038176449.1 | 16.14654 | 7.492922 | 3.534282 |  | 2.120069 | 0.034 | 0.555556 | VASH2 |
| rna-XM_038176514.1 | 5.217755 | -5.82042 | 2.552202 |  | -2.28055 | 0.022575 | NA | FBXL4 |
| rna-XM_038176521.1 | 56.6904 | 2.083862 | 0.756659 |  | 2.754032 | 0.005887 | 0.216564 | TRAF5 |
| rna-XM_038176539.1 | 29.09874 | -8.30009 | 3.238275 |  | -2.56312 | 0.010374 | 0.306995 | REPS1 |
| rna-XM_038176614.1 | 129.1049 | 1.388277 | 0.657376 |  | 2.111847 | 0.0347 | 0.560285 | SPAST |
| rna-XM_038176624.1 | 61.08905 | 9.412805 | 2.959781 |  | 3.180236 | 0.001472 | 0.08852 | LOC101805264 |
| rna-XM_038176659.1 | 53.21845 | -3.34646 | 1.546812 |  | -2.16346 | 0.030506 | 0.534889 | IBTK |
| rna-XM_038176683.1 | 317.7904 | 11.79202 | 2.251073 |  | 5.238399 | 1.62E-07 | 5.15E-05 | SLC29A1 |
| rna-XM_038176710.1 | 178.9348 | 1.103483 | 0.506611 |  | 2.178164 | 0.029394 | 0.528936 | YWHAQ |
| rna-XM_038176718.1 | 127.1285 | -1.40682 | 0.443079 |  | -3.17509 | 0.001498 | 0.089554 | FAT3 |
| rna-XM_038176768.1 | 43.41498 | -8.87731 | 3.0992 |  | -2.86439 | 0.004178 | 0.174149 | MANEA |
| rna-XM_038176769.1 | 48.36421 | -9.03322 | 1.470901 |  | -6.14128 | 8.19E-10 | 4.88E-07 | RNF144A |
| rna-XM_038176817.1 | 57.86955 | 2.465399 | 0.964242 |  | 2.556825 | 0.010563 | 0.309729 | LOC101789505 |
| rna-XM_038176824.1 | 129.8146 | -4.03859 | 1.645964 |  | -2.45363 | 0.014142 | 0.362207 | HMBOX1 |
| rna-XM_038176831.1 | 17.83148 | 7.636958 | 1.977876 |  | 3.861192 | 0.000113 | 0.012564 | CHRM3 |
| rna-XM_038176850.1 | 1074.659 | -3.01093 | 0.94237 |  | -3.19506 | 0.001398 | 0.08544 | LOC110354783 |
| rna-XM_038176891.1 | 14.7417 | 4.906476 | 2.404995 |  | 2.04012 | 0.041338 | 0.604446 | MED23 |
| rna-XM_038176936.1 | 19.93329 | -7.75415 | 3.624576 |  | -2.13933 | 0.032409 | 0.548798 | PROX1 |
| rna-XM_038176990.1 | 4.23844 | -5.52051 | 2.711823 |  | -2.03572 | 0.041778 | NA | LOC101793288 |
| rna-XM_038176991.1 | 10.22153 | 6.833979 | 2.213224 |  | 3.087793 | 0.002016 | 0.107735 | GSG1 |
| rna-XM_038177018.1 | 19.01529 | 6.754215 | 2.779357 |  | 2.430135 | 0.015093 | 0.371635 | EDARADD |
| rna-XM_038177031.1 | 47.96143 | -9.02116 | 1.618303 |  | -5.57446 | 2.48E-08 | 9.61E-06 | GSG1 |
| rna-XM_038177032.1 | 77.13433 | -3.43349 | 1.321237 |  | -2.5987 | 0.009358 | 0.287831 | PLCB4 |
| rna-XM_038177077.1 | 31.41127 | -5.59527 | 1.664031 |  | -3.36248 | 0.000772 | 0.055424 | GSG1 |
| rna-XM_038177116.1 | 198.1582 | 1.66324 | 0.537254 |  | 3.095819 | 0.001963 | 0.10582 | PRDM1 |
| rna-XM_038177125.1 | 4.738434 | 5.724029 | 2.665372 |  | 2.147554 | 0.031749 | NA | GLP1R |
| rna-XM_038177126.1 | 100.281 | 2.719927 | 1.358931 |  | 2.00152 | 0.045336 | 0.626311 | ROS1 |
| rna-XM_038177146.1 | 27.24599 | 8.247888 | 3.264594 |  | 2.526467 | 0.011522 | 0.323131 | MMUT |
| rna-XM_038177178.1 | 2047.775 | -3.00041 | 0.965262 |  | -3.10839 | 0.001881 | 0.103048 | LOC110354782 |
| rna-XM_038177247.1 | 10.53998 | -6.83499 | 2.09645 |  | -3.26027 | 0.001113 | 0.072484 | LOC101802235 |
| rna-XM_038177351.1 | 274.9189 | -1.58246 | 0.773364 |  | -2.0462 | 0.040737 | 0.600533 | NCOA1 |
| rna-XM_038177352.1 | 251.3348 | 2.846308 | 1.316468 |  | 2.162079 | 0.030612 | 0.535533 | NCOA1 |
| rna-XM_038177388.1 | 895.0222 | -2.59135 | 0.8829 |  | -2.93504 | 0.003335 | 0.15151 | PUM2 |
| rna-XM_038177405.1 | 212.6286 | 1.074515 | 0.536037 |  | 2.004553 | 0.045011 | 0.623719 | TTK |
| rna-XM_038177429.1 | 277.1653 | 2.117793 | 0.771158 |  | 2.746248 | 0.006028 | 0.219577 | IL20RA |
| rna-XM_038177503.1 | 51.53721 | -9.12477 | 1.414812 |  | -6.44946 | 1.12E-10 | 7.18E-08 | PRKN |
| rna-XM_038177504.1 | 37.54199 | 8.71095 | 1.533027 |  | 5.682188 | 1.33E-08 | 5.48E-06 | PRKN |
| rna-XM_038177507.1 | 15.04042 | 7.390619 | 3.567151 |  | 2.071855 | 0.038279 | 0.587974 | SYTL2 |
| rna-XM_038177561.1 | 10.53562 | 6.876785 | 2.124716 |  | 3.236566 | 0.00121 | 0.077253 | HTR1E |
| rna-XM_038177603.1 | 589.6772 | 1.466872 | 0.460001 |  | 3.188844 | 0.001428 | 0.087016 | SYNE1 |
| rna-XM_038177604.1 | 83.51767 | -9.82116 | 1.770541 |  | -5.54698 | 2.91E-08 | 1.10E-05 | SYNE1 |
| rna-XM_038177648.1 | 49.3867 | -9.0634 | 1.543082 |  | -5.87357 | 4.27E-09 | 2.03E-06 | ASAP2 |
| rna-XM_038177659.1 | 9.119686 | 5.681297 | 2.210663 |  | 2.569952 | 0.010171 | 0.303159 | XPO5 |
| rna-XM_038177682.1 | 1545.106 | 1.110132 | 0.364403 |  | 3.046439 | 0.002316 | 0.118549 | ASXL2 |
| rna-XM_038177723.1 | 2073.082 | -1.75844 | 0.80716 |  | -2.17855 | 0.029365 | 0.528936 | COL12A1 |
| rna-XM_038177749.1 | 30.47841 | 2.942684 | 1.482444 |  | 1.985022 | 0.047142 | 0.636791 | NCOA7 |
| rna-XM_038177780.1 | 254.5444 | 3.054929 | 0.888143 |  | 3.439683 | 0.000582 | 0.045247 | TLR1-A |
| rna-XM_038177790.1 | 13.2781 | 7.210735 | 3.648151 |  | 1.976545 | 0.048093 | 0.64201 | TLR2 |
| rna-XM_038177815.1 | 152.2231 | 10.73014 | 2.763779 |  | 3.882415 | 0.000103 | 0.011816 | HERC3 |
| rna-XM_038177923.1 | 414.1311 | -12.1312 | 1.580074 |  | -7.6776 | 1.62E-14 | 2.64E-11 | TET2 |
| rna-XM_038177988.1 | 1009.191 | 1.253759 | 0.513811 |  | 2.440118 | 0.014682 | 0.36788 | ARHGAP24 |
| rna-XM_038178041.1 | 121.9962 | 2.281741 | 0.520553 |  | 4.3833 | 1.17E-05 | 0.001967 | ZNF827 |
| rna-XM_038178048.1 | 36.33048 | -1.61079 | 0.748556 |  | -2.15186 | 0.031408 | 0.541913 | OTUD4 |
| rna-XM_038178059.1 | 37.6086 | -4.44297 | 1.759638 |  | -2.52493 | 0.011572 | 0.323335 | MARCHF1 |
| rna-XM_038178097.1 | 183.4289 | -1.78097 | 0.546085 |  | -3.26134 | 0.001109 | 0.072393 | ADAMTS3 |
| rna-XM_038178101.1 | 25.02917 | -8.0827 | 3.29994 |  | -2.44935 | 0.014312 | 0.364164 | PPM1K |
| rna-XM_038178114.1 | 42.20584 | -3.9897 | 1.932159 |  | -2.06489 | 0.038933 | 0.591366 | CXXC4 |
| rna-XM_038178169.1 | 395.3775 | 1.053753 | 0.306925 |  | 3.433252 | 0.000596 | 0.045766 | N4BP2 |
| rna-XM_038178195.1 | 170.828 | 1.19909 | 0.465973 |  | 2.573303 | 0.010073 | 0.301633 | KIAA1109 |
| rna-XM_038178252.1 | 18.76965 | -6.69348 | 2.786022 |  | -2.40252 | 0.016282 | 0.389419 | EVC |
| rna-XM_038178253.1 | 4.857202 | 5.760114 | 2.602671 |  | 2.213154 | 0.026887 | NA | UVRAG |
| rna-XM_038178316.1 | 2324.56 | 1.161345 | 0.238357 |  | 4.872289 | 1.10E-06 | 0.000257 | SEL1L3 |
| rna-XM_038178334.1 | 5.604693 | -5.92422 | 2.56739 |  | -2.30749 | 0.021028 | 0.445651 | CENPU |
| rna-XM_038178335.1 | 159.6423 | -1.45375 | 0.736533 |  | -1.97378 | 0.048407 | 0.644445 | LOC101805095 |
| rna-XM_038178364.1 | 16.13955 | 7.491782 | 2.052474 |  | 3.650123 | 0.000262 | 0.023816 | RNF4 |
| rna-XM_038178366.1 | 23.16156 | -7.9706 | 3.380288 |  | -2.35797 | 0.018375 | 0.41689 | ZGRF1 |
| rna-XM_038178374.1 | 24.78131 | -8.0682 | 3.342935 |  | -2.41351 | 0.0158 | 0.382016 | ZGRF1 |
| rna-XM_038178392.1 | 126.4165 | -2.1334 | 0.503709 |  | -4.23539 | 2.28E-05 | 0.003452 | RGS12 |
| rna-XM_038178402.1 | 5.470029 | -5.88898 | 2.582366 |  | -2.28046 | 0.02258 | 0.463469 | TRIM2 |
| rna-XM_038178416.1 | 93.06186 | 3.192116 | 1.29526 |  | 2.464459 | 0.013722 | 0.357525 | NPNT |
| rna-XM_038178434.1 | 277.254 | 1.533223 | 0.661419 |  | 2.318081 | 0.020445 | 0.439085 | ARAP2 |
| rna-XM_038178463.1 | 113.7313 | -1.98861 | 0.389794 |  | -5.10169 | 3.37E-07 | 9.64E-05 | PTCD3 |
| rna-XM_038178557.1 | 6.247389 | 6.124534 | 2.656872 |  | 2.305167 | 0.021157 | 0.447281 | PURG |
| rna-XM_038178574.1 | 22.78363 | -5.53251 | 1.549987 |  | -3.56939 | 0.000358 | 0.030725 | PROM1 |
| rna-XM_038178590.1 | 4.070409 | 5.504682 | 2.788057 |  | 1.974379 | 0.048339 | NA | BBS12 |
| rna-XM_038178639.1 | 30.00674 | -8.34442 | 3.230768 |  | -2.5828 | 0.0098 | 0.296219 | DYSF |
| rna-XM_038178670.1 | 23.82143 | 8.054002 | 3.338403 |  | 2.412531 | 0.015842 | 0.382793 | C4H4orf48 |
| rna-XM_038178675.1 | 44.65091 | 2.799289 | 1.099975 |  | 2.544866 | 0.010932 | 0.31428 | NDST4 |
| rna-XM_038178689.1 | 42.1125 | -8.83334 | 3.071644 |  | -2.87577 | 0.00403 | 0.171476 | WDFY3 |
| rna-XM_038178713.1 | 6.551512 | 6.190959 | 2.571078 |  | 2.407923 | 0.016044 | 0.385672 | LOC113844757 |
| rna-XM_038178793.1 | 202.6931 | 4.282231 | 1.622212 |  | 2.639748 | 0.008297 | 0.267378 | SORCS2 |
| rna-XM_038178835.1 | 21.98798 | 7.939102 | 1.896588 |  | 4.185992 | 2.84E-05 | 0.004146 | FRYL |
| rna-XM_038178836.1 | 15.70782 | 7.453485 | 1.892866 |  | 3.937672 | 8.23E-05 | 0.009801 | FRYL |
| rna-XM_038178837.1 | 2027.748 | -1.4145 | 0.714603 |  | -1.97942 | 0.047769 | 0.64019 | FRYL |
| rna-XM_038178841.1 | 48.51057 | 9.080219 | 2.162044 |  | 4.199831 | 2.67E-05 | 0.003931 | FRYL |
| rna-XM_038178851.1 | 591.0193 | -3.12346 | 1.098543 |  | -2.84328 | 0.004465 | 0.180559 | RHOG |
| rna-XM_038178861.1 | 162.341 | -5.82882 | 2.630367 |  | -2.21597 | 0.026693 | 0.503351 | PDE5A |
| rna-XM_038178930.1 | 214.5301 | 1.047804 | 0.515745 |  | 2.03163 | 0.042191 | 0.609108 | SCOC |
| rna-XM_038178935.1 | 103.4212 | 4.030491 | 1.389709 |  | 2.90024 | 0.003729 | 0.163305 | EGR4 |
| rna-XM_038178993.1 | 1074.541 | -1.21167 | 0.338079 |  | -3.58398 | 0.000338 | 0.029531 | FAT1 |
| rna-XM_038179114.1 | 7.033166 | 6.293025 | 2.72209 |  | 2.311836 | 0.020787 | 0.443248 | AKAP6 |
| rna-XM_038179130.1 | 44.33828 | 8.950449 | 3.063163 |  | 2.921963 | 0.003478 | 0.155943 | FOXN3 |
| rna-XM_038179164.1 | 22.01329 | 7.940111 | 3.372927 |  | 2.354072 | 0.018569 | 0.418752 | LOC119716992 |
| rna-XM_038179238.1 | 66.79331 | 9.541481 | 1.603257 |  | 5.951313 | 2.66E-09 | 1.39E-06 | MOB2 |
| rna-XM_038179245.1 | 394.9851 | 1.221053 | 0.569 |  | 2.145964 | 0.031876 | 0.545626 | MARK3 |
| rna-XM_038179250.1 | 25.42964 | 8.147824 | 1.924886 |  | 4.232888 | 2.31E-05 | 0.003477 | MARK3 |
| rna-XM_038179257.1 | 16.20119 | -7.45523 | 3.510167 |  | -2.1239 | 0.033679 | 0.554394 | SOX5 |
| rna-XM_038179310.1 | 45.28574 | 8.014571 | 2.58224 |  | 3.103728 | 0.001911 | 0.103777 | PLEKHA7 |
| rna-XM_038179321.1 | 15.46021 | 7.430546 | 3.543551 |  | 2.09692 | 0.036001 | 0.569482 | SOX6 |
| rna-XM_038179340.1 | 59.68826 | -9.33652 | 2.986955 |  | -3.12577 | 0.001773 | 0.099714 | ATXN7L1 |
| rna-XM_038179409.1 | 48.50683 | -5.71701 | 2.709492 |  | -2.10999 | 0.034859 | 0.560548 | PPP6R3 |
| rna-XM_038179411.1 | 92.95834 | 6.549323 | 1.934079 |  | 3.386275 | 0.000708 | 0.052027 | PPP6R3 |
| rna-XM_038179436.1 | 63.43036 | -1.61264 | 0.630149 |  | -2.55914 | 0.010493 | 0.309143 | FBXO34 |
| rna-XM_038179453.1 | 5104.927 | -1.06252 | 0.511299 |  | -2.07808 | 0.037702 | 0.5831 | NID2 |
| rna-XM_038179463.1 | 4.311028 | 5.58843 | 2.768789 |  | 2.018366 | 0.043553 | NA | LOC119717029 |
| rna-XM_038179509.1 | 19.79484 | 2.345432 | 1.08475 |  | 2.162187 | 0.030604 | 0.535533 | LOC119717036 |
| rna-XM_038179533.1 | 144.078 | 5.636134 | 1.811348 |  | 3.111569 | 0.001861 | 0.102244 | LOC101798382 |
| rna-XM_038179553.1 | 15.93628 | 4.519064 | 1.910816 |  | 2.364992 | 0.01803 | 0.412733 | LOC113843744 |
| rna-XM_038179561.1 | 103.3459 | 5.438997 | 1.661047 |  | 3.27444 | 0.001059 | 0.070316 | CEP170B |
| rna-XM_038179563.1 | 24.45001 | 8.091516 | 3.460866 |  | 2.338004 | 0.019387 | 0.427689 | CEP170B |
| rna-XM_038179566.1 | 516.0428 | 12.49161 | 1.346357 |  | 9.278081 | 1.73E-20 | 5.89E-17 | CEP170B |
| rna-XM_038179588.1 | 1244.648 | -1.35033 | 0.419647 |  | -3.21778 | 0.001292 | 0.081338 | KLC1 |
| rna-XM_038179608.1 | 14.05203 | 7.293042 | 3.584797 |  | 2.034436 | 0.041908 | 0.607175 | TRAF3 |
| rna-XM_038179638.1 | 588.7633 | 1.068261 | 0.372896 |  | 2.864768 | 0.004173 | 0.174149 | LOC101804808 |
| rna-XM_038179683.1 | 357.06 | -1.56009 | 0.334103 |  | -4.66948 | 3.02E-06 | 0.000609 | TCIRG1 |
| rna-XM_038179708.1 | 31.25589 | -1.8486 | 0.80311 |  | -2.30181 | 0.021346 | 0.448759 | VPS37C |
| rna-XM_038179719.1 | 474.197 | -2.01807 | 0.641245 |  | -3.14712 | 0.001649 | 0.09593 | LOC101792854 |
| rna-XM_038179758.1 | 587.0021 | -1.31389 | 0.426407 |  | -3.0813 | 0.002061 | 0.109391 | DAAM1 |
| rna-XM_038179782.1 | 47.85663 | 6.591837 | 2.613917 |  | 2.521824 | 0.011675 | 0.324757 | PPFIA1 |
| rna-XM_038179789.1 | 319.9983 | -1.32903 | 0.533647 |  | -2.49046 | 0.012758 | 0.340978 | SSRP1 |
| rna-XM_038179811.1 | 10.19562 | -6.78751 | 2.248847 |  | -3.01822 | 0.002543 | 0.126209 | LOC101800968 |
| rna-XM_038179816.1 | 469.7321 | 1.702393 | 0.435384 |  | 3.910098 | 9.23E-05 | 0.010819 | CD44 |
| rna-XM_038179871.1 | 15.35573 | 7.420237 | 1.974346 |  | 3.758327 | 0.000171 | 0.017301 | CFTR |
| rna-XM_038179945.1 | 395.245 | 1.00404 | 0.305109 |  | 3.290758 | 0.000999 | 0.067039 | HIF1A |
| rna-XM_038179959.1 | 461.7014 | 1.631637 | 0.367426 |  | 4.440725 | 8.97E-06 | 0.001572 | JDP2 |
| rna-XM_038179964.1 | 13.83166 | 7.269867 | 3.609738 |  | 2.01396 | 0.044014 | 0.620089 | LOC101805113 |
| rna-XM_038179965.1 | 34.79298 | 8.600605 | 3.197771 |  | 2.689562 | 0.007155 | 0.247553 | LOC101799876 |
| rna-XM_038180024.1 | 18.75696 | 7.709595 | 3.497352 |  | 2.204409 | 0.027496 | 0.511105 | RGS6 |
| rna-XM_038180032.1 | 186.4212 | -1.03656 | 0.408075 |  | -2.54012 | 0.011081 | 0.316459 | CIPC |
| rna-XM_038180038.1 | 194.6748 | -8.67797 | 2.387206 |  | -3.6352 | 0.000278 | 0.025116 | NUP37 |
| rna-XM_038180074.1 | 7.906672 | 6.462279 | 2.355787 |  | 2.74315 | 0.006085 | 0.220842 | TRIM9 |
| rna-XM_038180118.1 | 21.90407 | 7.933032 | 3.361567 |  | 2.359921 | 0.018279 | 0.415202 | SHANK2 |
| rna-XM_038180124.1 | 160.5359 | -2.15726 | 0.379827 |  | -5.67959 | 1.35E-08 | 5.51E-06 | IRAG1 |
| rna-XM_038180229.1 | 10423.77 | 2.194897 | 0.828202 |  | 2.650195 | 0.008045 | 0.262529 | FOS |
| rna-XM_038180239.1 | 21.04716 | -4.20916 | 1.846006 |  | -2.28014 | 0.022599 | 0.463469 | CCDC85C |
| rna-XM_038180256.1 | 42.81701 | 3.91346 | 1.695786 |  | 2.307755 | 0.021013 | 0.445651 | MUC2 |
| rna-XM_038180263.1 | 110.8535 | 1.598678 | 0.783133 |  | 2.041387 | 0.041212 | 0.603946 | CCDC88C |
| rna-XM_038180282.1 | 60.04113 | -1.62986 | 0.699103 |  | -2.33135 | 0.019735 | 0.431683 | ABCD4 |
| rna-XM_038180294.1 | 63.37993 | -9.4231 | 2.936618 |  | -3.20883 | 0.001333 | 0.083196 | RAB3IL1 |
| rna-XM_038180393.1 | 212.4294 | -1.23773 | 0.375406 |  | -3.29703 | 0.000977 | 0.06618 | KIAA0586 |
| rna-XM_038180411.1 | 87.95281 | 1.7689 | 0.830463 |  | 2.130017 | 0.03317 | 0.551764 | GPR132 |
| rna-XM_038180429.1 | 8.010128 | 6.481934 | 2.26129 |  | 2.866476 | 0.004151 | 0.173833 | LOC101790957 |
| rna-XM_038180505.1 | 78.82031 | -1.18004 | 0.59293 |  | -1.99019 | 0.04657 | 0.632985 | L3HYPDH |
| rna-XM_038180507.1 | 53.58584 | 1.235456 | 0.535581 |  | 2.306759 | 0.021068 | 0.445651 | LOC119713062 |
| rna-XM_038180550.1 | 24.90762 | -8.07564 | 1.922149 |  | -4.20136 | 2.65E-05 | 0.00392 | DNAL1 |
| rna-XM_038180554.1 | 411.7489 | -2.48106 | 0.923942 |  | -2.6853 | 0.007246 | 0.249028 | SLC43A3 |
| rna-XM_038180678.1 | 103.0387 | 3.078385 | 0.628503 |  | 4.897961 | 9.68E-07 | 0.000231 | UBR1 |
| rna-XM_038180698.1 | 87.99334 | 1.095218 | 0.439556 |  | 2.491644 | 0.012715 | 0.340156 | PHF21A |
| rna-XM_038180705.1 | 8.91086 | -6.59232 | 2.4408 |  | -2.70088 | 0.006916 | 0.242508 | ANKS1B |
| rna-XM_038180751.1 | 949.9494 | 1.057161 | 0.254313 |  | 4.156935 | 3.23E-05 | 0.00455 | SPTY2D1 |
| rna-XM_038180782.1 | 313.3003 | 2.858984 | 1.424987 |  | 2.006322 | 0.044822 | 0.623498 | TMEM87A |
| rna-XM_038180784.1 | 237.9694 | 3.904174 | 1.727214 |  | 2.260388 | 0.023797 | 0.474174 | TMEM87A |
| rna-XM_038180796.1 | 969.6728 | -1.04368 | 0.467309 |  | -2.23338 | 0.025524 | 0.493193 | ENTPD5 |
| rna-XM_038180804.1 | 241.0393 | -1.4204 | 0.669496 |  | -2.12159 | 0.033872 | 0.555287 | ZRANB1 |
| rna-XM_038180809.1 | 32.72714 | -8.46951 | 3.1705 |  | -2.67135 | 0.007555 | 0.253343 | CTBP2 |
| rna-XM_038180881.1 | 204.0582 | -11.1098 | 3.906977 |  | -2.84358 | 0.004461 | 0.180559 | ABLIM1 |
| rna-XM_038180908.1 | 17.19202 | 7.583597 | 3.484082 |  | 2.176641 | 0.029507 | 0.528936 | LOC101791050 |
| rna-XM_038180921.1 | 212.9532 | 11.21436 | 3.906969 |  | 2.870348 | 0.0041 | 0.172489 | SLC2A13 |
| rna-XM_038180964.1 | 22.09369 | -7.9028 | 3.388712 |  | -2.3321 | 0.019696 | 0.431661 | BTRC |
| rna-XM_038180991.1 | 17.48794 | 7.607989 | 3.53759 |  | 2.150614 | 0.031507 | 0.542463 | PAPSS2 |
| rna-XM_038181076.1 | 1899.43 | 1.653266 | 0.59215 |  | 2.791974 | 0.005239 | 0.200596 | WDFY4 |
| rna-XM_038181148.1 | 153.834 | 9.781844 | 1.947838 |  | 5.021898 | 5.12E-07 | 0.000137 | FGFR2 |
| rna-XM_038181194.1 | 39.79185 | -8.75138 | 3.251344 |  | -2.69162 | 0.007111 | 0.246604 | GHITM |
| rna-XM_038181246.1 | 34.69691 | 2.740559 | 1.292082 |  | 2.121041 | 0.033918 | 0.555289 | DMBT1 |
| rna-XM_038181259.1 | 54.85704 | -9.21465 | 3.003964 |  | -3.0675 | 0.002159 | 0.112035 | NDST2 |
| rna-XM_038181290.1 | 49.86831 | 9.11983 | 1.45792 |  | 6.255369 | 3.97E-10 | 2.44E-07 | ATRNL1 |
| rna-XM_038181361.1 | 129.7938 | -10.4572 | 2.308049 |  | -4.53073 | 5.88E-06 | 0.001092 | CCAR1 |
| rna-XM_038181382.1 | 33.92839 | 6.161018 | 2.691014 |  | 2.289478 | 0.022052 | 0.457509 | USP54 |
| rna-XM_038181395.1 | 11.39815 | -6.94745 | 2.187562 |  | -3.17589 | 0.001494 | 0.089544 | SORBS1 |
| rna-XM_038181453.1 | 24.76322 | 8.110381 | 3.27645 |  | 2.475356 | 0.01331 | 0.350489 | GPAM |
| rna-XM_038181458.1 | 54.47715 | -1.01732 | 0.513041 |  | -1.98292 | 0.047376 | 0.638348 | NR2C1 |
| rna-XM_038181532.1 | 48.28885 | -1.07562 | 0.509437 |  | -2.11138 | 0.03474 | 0.560548 | COL13A1 |
| rna-XM_038181574.1 | 29.89916 | -1.9631 | 0.818734 |  | -2.39772 | 0.016497 | 0.391814 | BRD1 |
| rna-XM_038181586.1 | 58.45857 | 9.349437 | 2.973911 |  | 3.143819 | 0.001668 | 0.096717 | ZMIZ1 |
| rna-XM_038181595.1 | 765.1487 | 1.075117 | 0.422541 |  | 2.544409 | 0.010946 | 0.31428 | ZMIZ1 |
| rna-XM_038181641.1 | 66.90015 | 1.811499 | 0.798834 |  | 2.267678 | 0.023349 | 0.470045 | ANK3 |
| rna-XM_038181713.1 | 1247.278 | -1.01528 | 0.202773 |  | -5.007 | 5.53E-07 | 0.000145 | SGPL1 |
| rna-XM_038181722.1 | 575.903 | 1.709328 | 0.358274 |  | 4.771005 | 1.83E-06 | 0.000393 | DLG5 |
| rna-XM_038181728.1 | 23.45577 | 8.031198 | 1.994203 |  | 4.027272 | 5.64E-05 | 0.007202 | MAP3K19 |
| rna-XM_038181738.1 | 57.62707 | 9.328646 | 2.975084 |  | 3.135591 | 0.001715 | 0.098558 | MGAT5 |
| rna-XM_038181749.1 | 32.54458 | -8.46154 | 3.192849 |  | -2.65015 | 0.008046 | 0.262529 | BAZ2B |
| rna-XM_038181773.1 | 21.15641 | 7.882778 | 1.7639 |  | 4.468947 | 7.86E-06 | 0.001392 | CLASP1 |
| rna-XM_038181825.1 | 20.19506 | 7.815621 | 1.800038 |  | 4.341919 | 1.41E-05 | 0.002265 | LOC101793492 |
| rna-XM_038181837.1 | 31.07286 | -8.39461 | 3.236573 |  | -2.59367 | 0.009496 | 0.289696 | KANSL1L |
| rna-XM_038181931.1 | 90.04524 | 1.653566 | 0.73692 |  | 2.243889 | 0.02484 | 0.486738 | PARP9 |
| rna-XM_038182011.1 | 1137.474 | 1.090414 | 0.453406 |  | 2.404941 | 0.016175 | 0.387841 | ITGA4 |
| rna-XM_038182044.1 | 30.88741 | -8.38609 | 1.78 |  | -4.71129 | 2.46E-06 | 0.00051 | NEMP2 |
| rna-XM_038182075.1 | 97.75308 | 10.09102 | 2.97736 |  | 3.389251 | 0.000701 | 0.051668 | TTN |
| rna-XM_038182093.1 | 444.3193 | 2.293649 | 1.042599 |  | 2.199935 | 0.027811 | 0.513527 | TTN |
| rna-XM_038182227.1 | 15.97466 | 7.477419 | 3.567968 |  | 2.095708 | 0.036108 | 0.570199 | MRAS |
| rna-XM_038182231.1 | 1779.207 | -13.2834 | 3.545529 |  | -3.74651 | 0.000179 | 0.017943 | MYLK |
| rna-XM_038182253.1 | 84.76055 | 1.863114 | 0.810883 |  | 2.297637 | 0.021582 | 0.451188 | MLPH |
| rna-XM_038182314.1 | 14.04353 | 7.291573 | 3.61867 |  | 2.014987 | 0.043906 | 0.620088 | DPP10 |
| rna-XM_038182328.1 | 265.7309 | 1.319408 | 0.670849 |  | 1.966772 | 0.049209 | 0.648969 | DNAH7 |
| rna-XM_038182337.1 | 94.91629 | -10.0057 | 2.902774 |  | -3.44694 | 0.000567 | 0.044141 | ANKRD44 |
| rna-XM_038182364.1 | 70.00684 | 1.998953 | 0.579727 |  | 3.44809 | 0.000565 | 0.044044 | TLK1 |
| rna-XM_038182386.1 | 500.4059 | 2.157767 | 0.734367 |  | 2.938267 | 0.003301 | 0.150672 | NR4A2 |
| rna-XM_038182404.1 | 42.5245 | -8.84733 | 3.069336 |  | -2.88249 | 0.003945 | 0.169591 | ST3GAL6 |
| rna-XM_038182495.1 | 27.00696 | -1.44216 | 0.667678 |  | -2.15996 | 0.030776 | 0.537641 | RAPH1 |
| rna-XM_038182627.1 | 25.5569 | 8.156379 | 1.859352 |  | 4.386679 | 1.15E-05 | 0.001945 | ZNF644 |
| rna-XM_038182647.1 | 411.0752 | 1.081891 | 0.546139 |  | 1.98098 | 0.047593 | 0.638979 | LRRC8D |
| rna-XM_038182705.1 | 22.7488 | -1.38941 | 0.668971 |  | -2.07693 | 0.037808 | 0.583364 | LOC119717559 |
| rna-XM_038182713.1 | 121.9353 | -3.10931 | 1.478818 |  | -2.10257 | 0.035504 | 0.565716 | LOC119717572 |
| rna-XM_038182746.1 | 25.46485 | 4.384769 | 1.778961 |  | 2.464792 | 0.013709 | 0.357525 | HFM1 |
| rna-XM_038182764.1 | 195.2597 | 1.210825 | 0.566513 |  | 2.13733 | 0.032571 | 0.549611 | TGFBR3 |
| rna-XM_038182862.1 | 15.75664 | -7.41508 | 3.525828 |  | -2.10307 | 0.035459 | 0.565716 | DPYD |
| rna-XM_038182870.1 | 22.86893 | -7.9525 | 3.326118 |  | -2.39093 | 0.016806 | 0.396324 | LOC101803722 |
| rna-XM_038182911.1 | 4.566299 | -5.628 | 2.652931 |  | -2.12143 | 0.033886 | NA | FNDC7 |
| rna-XM_038182915.1 | 98.76668 | 3.382998 | 1.605491 |  | 2.107142 | 0.035105 | 0.563439 | LOC106019440 |
| rna-XM_038182948.1 | 18.36253 | 2.299708 | 1.087618 |  | 2.114444 | 0.034477 | 0.558247 | LOC101800711 |
| rna-XM_038182996.1 | 4.565377 | -5.62772 | 2.682461 |  | -2.09797 | 0.035908 | NA | NPL |
| rna-XM_038183157.1 | 529.3034 | -1.94685 | 0.37724 |  | -5.16078 | 2.46E-07 | 7.30E-05 | MOB3C |
| rna-XM_038183181.1 | 24.79829 | -8.06934 | 3.286788 |  | -2.45508 | 0.014085 | 0.361769 | ELAVL4 |
| rna-XM_038183277.1 | 23.0801 | -7.96575 | 3.321245 |  | -2.39842 | 0.016466 | 0.391356 | LOC101802274 |
| rna-XM_038183359.1 | 227.3331 | -1.95124 | 0.552896 |  | -3.52913 | 0.000417 | 0.034235 | PTPRF |
| rna-XM_038183366.1 | 17.21661 | -7.54266 | 3.499343 |  | -2.15545 | 0.031127 | 0.539815 | ALG6 |
| rna-XM_038183372.1 | 209.5208 | -3.94685 | 1.962912 |  | -2.01071 | 0.044356 | 0.622228 | DDAH1 |
| rna-XM_038183434.1 | 20.60458 | 4.594623 | 2.105261 |  | 2.182449 | 0.029076 | 0.527353 | SENP5 |
| rna-XM_038183449.1 | 98.33059 | 1.450169 | 0.53318 |  | 2.719851 | 0.006531 | 0.232462 | MAP3K13 |
| rna-XM_038183473.1 | 23.89786 | 8.058804 | 3.351429 |  | 2.404587 | 0.016191 | 0.387968 | DLG1 |
| rna-XM_038183476.1 | 71.60531 | 1.852028 | 0.709702 |  | 2.609586 | 0.009065 | 0.282466 | TBL1XR1 |
| rna-XM_038183512.1 | 36.58603 | -8.63039 | 3.173375 |  | -2.71963 | 0.006536 | 0.232462 | PIK3CB |
| rna-XM_038183538.1 | 19.30275 | 7.750297 | 1.901516 |  | 4.075852 | 4.58E-05 | 0.006079 | MECOM |
| rna-XM_038183541.1 | 1126.88 | 1.041035 | 0.253966 |  | 4.099109 | 4.15E-05 | 0.005558 | MECOM |
| rna-XM_038183556.1 | 211.16 | 1.128864 | 0.540676 |  | 2.087875 | 0.036809 | 0.57576 | NAALADL2 |
| rna-XM_038183597.1 | 22.43041 | 7.967363 | 3.374376 |  | 2.361136 | 0.018219 | 0.414848 | PXYLP1 |
| rna-XM_038183598.1 | 133.2025 | -10.4948 | 1.447761 |  | -7.249 | 4.20E-13 | 4.77E-10 | PXYLP1 |
| rna-XM_038183629.1 | 74.5711 | 9.700512 | 2.908079 |  | 3.335711 | 0.000851 | 0.060013 | RPL35A |
| rna-XM_038183640.1 | 31.52636 | 2.049902 | 0.786849 |  | 2.605203 | 0.009182 | 0.283583 | LOC110354120 |
| rna-XM_038183654.1 | 33.73423 | -1.62853 | 0.804625 |  | -2.02396 | 0.042974 | 0.614633 | VEPH1 |
| rna-XM_038183678.1 | 47.89669 | 9.061971 | 3.105834 |  | 2.917726 | 0.003526 | 0.157701 | EIF4G1 |
| rna-XM_038183707.1 | 6.844436 | 5.255015 | 2.606609 |  | 2.016035 | 0.043796 | 0.619237 | SI |
| rna-XM_038183716.1 | 9.05275 | -6.61562 | 2.238474 |  | -2.95542 | 0.003122 | 0.145917 | LEKR1 |
| rna-XM_038183758.1 | 18.76973 | 7.709933 | 2.124345 |  | 3.629323 | 0.000284 | 0.025573 | SPSB4 |
| rna-XM_038183765.1 | 36.73803 | -3.97989 | 1.860499 |  | -2.13915 | 0.032423 | 0.548798 | SPSB4 |
| rna-XM_038183768.1 | 10.31268 | -6.8037 | 2.174745 |  | -3.1285 | 0.001757 | 0.099714 | LMO7 |
| rna-XM_038183892.1 | 14.26588 | -7.27138 | 3.647447 |  | -1.99355 | 0.046201 | 0.63158 | DGKD |
| rna-XM_038183900.1 | 368.7402 | 1.060549 | 0.460213 |  | 2.304475 | 0.021196 | 0.447849 | LOC101801425 |
| rna-XM_038183908.1 | 32.31024 | 6.615939 | 1.5365 |  | 4.30585 | 1.66E-05 | 0.002612 | ABCC9 |
| rna-XM_038183912.1 | 323.6434 | -1.94026 | 0.76546 |  | -2.53476 | 0.011252 | 0.319391 | LOC101797556 |
| rna-XM_038183916.1 | 48.48025 | -9.03646 | 3.024456 |  | -2.9878 | 0.00281 | 0.134667 | LOC101797556 |
| rna-XM_038183917.1 | 227.1183 | -1.64685 | 0.550028 |  | -2.99411 | 0.002752 | 0.132862 | LOC101797556 |
| rna-XM_038183926.1 | 220.4875 | -1.1032 | 0.268666 |  | -4.10619 | 4.02E-05 | 0.00541 | LOC101797556 |
| rna-XM_038183993.1 | 316.2098 | 10.82224 | 3.318017 |  | 3.261659 | 0.001108 | 0.072393 | SEPTIN2 |
| rna-XM_038184020.1 | 72.05708 | -1.15468 | 0.513496 |  | -2.24866 | 0.024534 | 0.482514 | OPHN1 |
| rna-XM_038184037.1 | 137.9764 | -1.04298 | 0.476119 |  | -2.1906 | 0.028481 | 0.521087 | POU2F1 |
| rna-XM_038184054.1 | 106.4741 | -10.1714 | 1.534594 |  | -6.62804 | 3.40E-11 | 2.41E-08 | AMER1 |
| rna-XM_038184069.1 | 191.2193 | 11.05906 | 1.265158 |  | 8.741249 | 2.31E-18 | 5.41E-15 | ARHGEF9 |
| rna-XM_038184091.1 | 1269.09 | -1.85337 | 0.478666 |  | -3.87196 | 0.000108 | 0.012203 | SH3BGRL |
| rna-XM_038184097.1 | 265.2477 | -1.53012 | 0.709938 |  | -2.15528 | 0.03114 | 0.539815 | LOC113840518 |
| rna-XM_038184110.1 | 41.20577 | -8.80173 | 2.279309 |  | -3.86158 | 0.000113 | 0.012564 | LOC113840518 |
| rna-XM_038184130.1 | 448.8537 | -1.08049 | 0.430624 |  | -2.50914 | 0.012103 | 0.331853 | LOC113841786 |
| rna-XM_038184131.1 | 41.33143 | -1.51062 | 0.730765 |  | -2.06718 | 0.038717 | 0.590353 | LOC113841786 |
| rna-XM_038184148.1 | 22.95748 | 8.000764 | 3.33737 |  | 2.397326 | 0.016515 | 0.39188 | DOCK11 |
| rna-XM_038184204.1 | 109.8135 | 10.25899 | 2.804408 |  | 3.658166 | 0.000254 | 0.023306 | LOC119717843 |
| rna-XM_038184217.1 | 29.02377 | 2.49785 | 1.129235 |  | 2.211984 | 0.026968 | 0.504973 | LOC119717844 |
| rna-XM_038184220.1 | 53.19326 | -3.38065 | 1.397312 |  | -2.41939 | 0.015546 | 0.378328 | LOC119717844 |
| rna-XM_038184223.1 | 120.8412 | 2.770813 | 0.528305 |  | 5.244717 | 1.57E-07 | 5.06E-05 | LMO7 |
| rna-XM_038184236.1 | 99.81899 | -2.50576 | 0.923019 |  | -2.71475 | 0.006633 | 0.235071 | LOC113843518 |
| rna-XM_038184315.1 | 40.58841 | 8.822788 | 1.615354 |  | 5.46183 | 4.71E-08 | 1.70E-05 | DLG3 |
| rna-XM_038184328.1 | 21.68733 | -4.53357 | 1.889274 |  | -2.39964 | 0.016411 | 0.391005 | LOC101793647 |
| rna-XM_038184384.1 | 49.97923 | 4.445444 | 2.142932 |  | 2.074468 | 0.038036 | 0.585677 | PTGFRN |
| rna-XM_038184409.1 | 16.79024 | -7.50651 | 3.504441 |  | -2.142 | 0.032194 | 0.546883 | PTGFRN |
| rna-XM_038184451.1 | 578.3464 | 12.65593 | 1.836198 |  | 6.892465 | 5.48E-12 | 4.68E-09 | STAG2 |
| rna-XM_038184455.1 | 59.91546 | -9.34191 | 1.590353 |  | -5.87411 | 4.25E-09 | 2.03E-06 | STAG2 |
| rna-XM_038184460.1 | 156.2079 | -1.54775 | 0.443295 |  | -3.49146 | 0.00048 | 0.038274 | EDA |
| rna-XM_038184476.1 | 1928.093 | 1.07664 | 0.285455 |  | 3.771659 | 0.000162 | 0.016536 | WDR44 |
| rna-XM_038184479.1 | 1067.886 | -1.67305 | 0.654145 |  | -2.55762 | 0.010539 | 0.309453 | SPRY3 |
| rna-XM_038184501.1 | 12.50425 | 7.124245 | 2.315928 |  | 3.076194 | 0.002097 | 0.110298 | PASD1 |
| rna-XM_038184512.1 | 240.0294 | 1.590748 | 0.638063 |  | 2.493089 | 0.012664 | 0.340143 | LOC119717898 |
| rna-XM_038184516.1 | 1263.305 | 1.564377 | 0.623986 |  | 2.507069 | 0.012174 | 0.332885 | LOC119717899 |
| rna-XM_038184605.1 | 14.94397 | -7.33856 | 3.564989 |  | -2.05851 | 0.039541 | 0.593752 | NRK |
| rna-XM_038184621.1 | 336.8813 | -5.62248 | 1.480212 |  | -3.79843 | 0.000146 | 0.015179 | BCL9 |
| rna-XM_038184712.1 | 68.52503 | 3.345312 | 1.484485 |  | 2.253517 | 0.024227 | 0.478475 | OTUD7A |
| rna-XM_038184749.1 | 47.64768 | -9.01163 | 1.470163 |  | -6.12968 | 8.81E-10 | 5.01E-07 | VGLL3 |
| rna-XM_038184750.1 | 164.2003 | -10.7964 | 2.724409 |  | -3.96285 | 7.41E-05 | 0.009052 | TPM1 |
| rna-XM_038184785.1 | 179.6491 | -10.9261 | 2.814427 |  | -3.88218 | 0.000104 | 0.011816 | TCF12 |
| rna-XM_038184788.1 | 71.51804 | -9.59725 | 3.069228 |  | -3.12693 | 0.001766 | 0.099714 | TCF12 |
| rna-XM_038184896.1 | 10.1509 | -5.79649 | 2.730191 |  | -2.12311 | 0.033745 | 0.554394 | GATM |
| rna-XM_038184898.1 | 13.25213 | -7.16544 | 1.975823 |  | -3.62656 | 0.000287 | 0.025777 | NUDT5 |
| rna-XM_038184906.1 | 233.913 | -5.65756 | 1.710538 |  | -3.30748 | 0.000941 | 0.064896 | ANXA2 |
| rna-XM_038184990.1 | 48.75296 | 9.08737 | 3.045732 |  | 2.98364 | 0.002848 | 0.135453 | HMG20A |
| rna-XM_038185105.1 | 20.48284 | -7.79335 | 3.439563 |  | -2.2658 | 0.023464 | 0.471855 | TP53BP1 |
| rna-XM_038185210.1 | 146.2629 | -1.1305 | 0.493515 |  | -2.29071 | 0.02198 | 0.456959 | AKAP13 |
| rna-XM_038185284.1 | 35.95845 | -8.60527 | 3.140732 |  | -2.73989 | 0.006146 | 0.221996 | SALL1 |
| rna-XM_038185304.1 | 42.13855 | 4.118516 | 1.907201 |  | 2.159456 | 0.030815 | 0.537696 | WDR59 |
| rna-XM_038185349.1 | 116.9356 | 4.186634 | 1.40092 |  | 2.98849 | 0.002804 | 0.134533 | DPY19L3 |
| rna-XM_038185390.1 | 22.48967 | 7.971607 | 1.711829 |  | 4.656777 | 3.21E-06 | 0.000641 | NUP93 |
| rna-XM_038185462.1 | 327.885 | -9.96728 | 1.383721 |  | -7.20324 | 5.88E-13 | 6.49E-10 | PLEKHG4 |
| rna-XM_038185476.1 | 24.00952 | 8.065723 | 3.324002 |  | 2.42651 | 0.015245 | 0.374179 | ZDHHC1 |
| rna-XM_038185487.1 | 16.30864 | 7.508148 | 2.44111 |  | 3.07571 | 0.0021 | 0.110298 | ACSF3 |
| rna-XM_038185519.1 | 79.27266 | -8.78067 | 1.488376 |  | -5.8995 | 3.65E-09 | 1.85E-06 | ROBO1 |
| rna-XM_038185630.1 | 187.8031 | 1.98722 | 0.887567 |  | 2.238952 | 0.025159 | 0.489443 | CARMIL2 |
| rna-XM_038185655.1 | 1497.314 | -9.68499 | 2.51984 |  | -3.8435 | 0.000121 | 0.01327 | NFAT5 |
| rna-XM_038185661.1 | 56.31466 | 9.295375 | 2.995322 |  | 3.103297 | 0.001914 | 0.103778 | PLCG2 |
| rna-XM_038185663.1 | 40.7602 | 8.829101 | 3.118971 |  | 2.830773 | 0.004644 | 0.185442 | PLCG2 |
| rna-XM_038185666.1 | 7.528297 | -6.34974 | 2.684823 |  | -2.36505 | 0.018028 | 0.412733 | SDR42E1 |
| rna-XM_038185694.1 | 333.1576 | 2.128522 | 0.588518 |  | 3.61675 | 0.000298 | 0.026465 | LRIG1 |
| rna-XM_038185728.1 | 14.26225 | 7.31437 | 3.60342 |  | 2.029841 | 0.042373 | 0.610613 | ATXN7 |
| rna-XM_038185814.1 | 201.9036 | -1.46925 | 0.710969 |  | -2.06654 | 0.038777 | 0.590553 | PHF2 |
| rna-XM_038185816.1 | 19.74753 | -7.74073 | 3.407228 |  | -2.27186 | 0.023095 | 0.467952 | PLXNB1 |
| rna-XM_038185912.1 | 28.3953 | -8.26474 | 3.226739 |  | -2.56133 | 0.010427 | 0.308098 | LOC119718225 |
| rna-XM_038185920.1 | 408.5984 | -4.67369 | 1.278402 |  | -3.65588 | 0.000256 | 0.023458 | LOC119718228 |
| rna-XM_038185948.1 | 22.67995 | 7.983545 | 3.344529 |  | 2.387046 | 0.016984 | 0.398586 | CACNA2D2 |
| rna-XM_038186050.1 | 902.6078 | -1.22557 | 0.207984 |  | -5.8926 | 3.80E-09 | 1.90E-06 | QARS1 |
| rna-XM_038186087.1 | 39.35306 | 8.778348 | 3.105281 |  | 2.826909 | 0.0047 | 0.186734 | FOXP1 |
| rna-XM_038186131.1 | 169.4343 | 1.191999 | 0.482098 |  | 2.472525 | 0.013416 | 0.351813 | SFMBT1 |
| rna-XM_038186141.1 | 66.15686 | -9.48494 | 2.930575 |  | -3.23654 | 0.00121 | 0.077253 | LOC119718292 |
| rna-XM_038186210.1 | 19.55311 | -7.72627 | 3.428509 |  | -2.25354 | 0.024225 | 0.478475 | TWF2 |
| rna-XM_038186235.1 | 27.01821 | -8.19309 | 3.321074 |  | -2.467 | 0.013625 | 0.356293 | C2CD5 |
| rna-XM_038186254.1 | 40.46109 | 8.818472 | 3.087656 |  | 2.856041 | 0.00429 | 0.176306 | PPARGC1B |
| rna-XM_038186268.1 | 27.85562 | 8.280168 | 3.246651 |  | 2.550372 | 0.010761 | 0.312979 | WWC1 |
| rna-XM_038186275.1 | 545.6076 | 1.693187 | 0.840516 |  | 2.014462 | 0.043961 | 0.620089 | LOC101800452 |
| rna-XM_038186296.1 | 1045.353 | 1.002045 | 0.420131 |  | 2.385075 | 0.017076 | 0.399478 | CYFIP2 |
| rna-XM_038186302.1 | 161.6567 | -1.11405 | 0.557961 |  | -1.99665 | 0.045864 | 0.629952 | LOC101797191 |
| rna-XM_038186315.1 | 14.23122 | 7.310855 | 3.602031 |  | 2.029648 | 0.042392 | 0.610661 | MAPK9 |
| rna-XM_038186319.1 | 19.3343 | 7.75293 | 3.425585 |  | 2.263242 | 0.023621 | 0.47273 | JAKMIP2 |
| rna-XM_038186321.1 | 127.9025 | -1.71469 | 0.664321 |  | -2.58111 | 0.009848 | 0.29731 | LOC101795967 |
| rna-XM_038186328.1 | 178.4728 | -2.09668 | 0.496872 |  | -4.21976 | 2.45E-05 | 0.003642 | FAM114A2 |
| rna-XM_038186373.1 | 14.7701 | -7.32202 | 2.06372 |  | -3.54797 | 0.000388 | 0.032443 | RANBP17 |
| rna-XM_038186389.1 | 169.15 | 2.011139 | 0.771656 |  | 2.606263 | 0.009154 | 0.28294 | FOXI1 |
| rna-XM_038186402.1 | 21.2035 | 3.924725 | 1.866161 |  | 2.103101 | 0.035457 | 0.565716 | PRELID2 |
| rna-XM_038186472.1 | 16.46616 | 7.521437 | 2.032007 |  | 3.701481 | 0.000214 | 0.020571 | CELF2 |
| rna-XM_038186480.1 | 97.4306 | 1.780254 | 0.74662 |  | 2.384419 | 0.017106 | 0.399942 | YIPF5 |
| rna-XM_038186500.1 | 161.435 | 10.81488 | 2.074037 |  | 5.214412 | 1.84E-07 | 5.72E-05 | SBF1 |
| rna-XM_038186520.1 | 483.0353 | 11.43373 | 3.366265 |  | 3.396564 | 0.000682 | 0.050605 | SBF1 |
| rna-XM_038186525.1 | 1035.895 | 1.241799 | 0.232861 |  | 5.332788 | 9.67E-08 | 3.21E-05 | PPP6R2 |
| rna-XM_038186528.1 | 39.89453 | 8.797948 | 3.348806 |  | 2.627189 | 0.008609 | 0.27309 | GFPT2 |
| rna-XM_038186538.1 | 54.42883 | 2.212067 | 0.988059 |  | 2.238801 | 0.025169 | 0.489443 | LOC101801606 |
| rna-XM_038186539.1 | 23.46625 | 8.03246 | 3.33492 |  | 2.408591 | 0.016014 | 0.385461 | LOC101801606 |
| rna-XM_038186548.1 | 62.52207 | -1.81696 | 0.849933 |  | -2.13776 | 0.032536 | 0.549611 | LOC106019744 |
| rna-XM_038186559.1 | 136.5659 | 10.57348 | 2.75905 |  | 3.832291 | 0.000127 | 0.01369 | NSD1 |
| rna-XM_038186564.1 | 14.07178 | 7.29439 | 3.658152 |  | 1.994009 | 0.046151 | 0.631359 | RCSD1 |
| rna-XM_038186626.1 | 902.3543 | -1.17126 | 0.362763 |  | -3.22871 | 0.001244 | 0.078822 | LOC119718385 |
| rna-XM_038186764.1 | 47.50265 | 9.050121 | 2.226366 |  | 4.064974 | 4.80E-05 | 0.006303 | GLYR1 |
| rna-XM_038186792.1 | 49.33817 | 9.104329 | 1.57695 |  | 5.773379 | 7.77E-09 | 3.43E-06 | PDGFA |
| rna-XM_038186843.1 | 70.88423 | -1.60141 | 0.711943 |  | -2.24935 | 0.024491 | 0.481912 | USP22 |
| rna-XM_038186853.1 | 418.6577 | 12.18968 | 1.267967 |  | 9.613564 | 7.01E-22 | 2.92E-18 | SDK1 |
| rna-XM_038186856.1 | 962.1394 | -2.68444 | 0.575866 |  | -4.66157 | 3.14E-06 | 0.00063 | SDK1 |
| rna-XM_038186897.1 | 14.89872 | 7.377481 | 3.566417 |  | 2.068598 | 0.038584 | 0.590158 | KATNIP |
| rna-XM_038186947.1 | 13.51922 | 7.237722 | 2.036882 |  | 3.553335 | 0.00038 | 0.032076 | CACNA1H |
| rna-XM_038186960.1 | 1810.104 | 1.045193 | 0.523934 |  | 1.994895 | 0.046054 | 0.630726 | PRKCB |
| rna-XM_038187006.1 | 44.38427 | -3.4478 | 1.410294 |  | -2.44474 | 0.014496 | 0.366395 | GRIN2A |
| rna-XM_038187023.1 | 47.64955 | -9.01185 | 1.560175 |  | -5.77618 | 7.64E-09 | 3.41E-06 | SREBF1 |
| rna-XM_038187028.1 | 183.9811 | 1.128927 | 0.465269 |  | 2.426395 | 0.01525 | 0.374179 | TOM1L2 |
| rna-XM_038187029.1 | 43.22549 | 7.946704 | 2.767484 |  | 2.871455 | 0.004086 | 0.172272 | ELFN1 |
| rna-XM_038187095.1 | 94.07438 | -9.9927 | 1.46199 |  | -6.835 | 8.20E-12 | 6.84E-09 | CLEC16A |
| rna-XM_038187122.1 | 147.4093 | -1.48114 | 0.53537 |  | -2.76657 | 0.005665 | 0.211099 | ZC3H7A |
| rna-XM_038187140.1 | 146.0821 | 1.540871 | 0.588975 |  | 2.616193 | 0.008892 | 0.278513 | WDR90 |
| rna-XM_038187185.1 | 569.7834 | 1.331485 | 0.319587 |  | 4.166269 | 3.10E-05 | 0.004418 | RILPL1 |
| rna-XM_038187191.1 | 78.62845 | -4.22136 | 1.89765 |  | -2.22452 | 0.026114 | 0.498865 | LOC113845344 |
| rna-XM_038187192.1 | 41.26553 | 8.847229 | 2.283771 |  | 3.873956 | 0.000107 | 0.01214 | LOC113845344 |
| rna-XM_038187232.1 | 49.93349 | 9.122439 | 1.892706 |  | 4.819788 | 1.44E-06 | 0.000321 | CLIP1 |
| rna-XM_038187263.1 | 19.34642 | 7.753399 | 1.962959 |  | 3.949852 | 7.82E-05 | 0.009435 | LOC101801322 |
| rna-XM_038187266.1 | 9.711424 | 6.760097 | 2.64625 |  | 2.554595 | 0.010631 | 0.310212 | LOC101804932 |
| rna-XM_038187290.1 | 19.38001 | -7.71352 | 3.636933 |  | -2.12089 | 0.033931 | 0.555289 | RNFT2 |
| rna-XM_038187322.1 | 26.76344 | -2.83868 | 1.445105 |  | -1.96434 | 0.04949 | 0.65071 | DAO |
| rna-XM_038187334.1 | 166.1673 | 1.657442 | 0.651406 |  | 2.544405 | 0.010946 | 0.31428 | ARVCF |
| rna-XM_038187340.1 | 424.7403 | 1.244355 | 0.427145 |  | 2.91319 | 0.003578 | 0.159062 | ARVCF |
| rna-XM_038187385.1 | 26.92255 | 8.231168 | 1.682135 |  | 4.893288 | 9.92E-07 | 0.000236 | TANGO2 |
| rna-XM_038187470.1 | 25.89317 | 5.691906 | 2.310387 |  | 2.463615 | 0.013754 | 0.357676 | VPREB3 |
| rna-XM_038187472.1 | 8.722546 | 6.603553 | 2.666228 |  | 2.47674 | 0.013259 | 0.349641 | LOC101803831 |
| rna-XM_038187477.1 | 42.6984 | 8.895818 | 1.652817 |  | 5.382217 | 7.36E-08 | 2.53E-05 | SRRM4 |
| rna-XM_038187516.1 | 14.30678 | -3.74571 | 1.901243 |  | -1.97014 | 0.048823 | 0.646687 | TPST2 |
| rna-XM_038187634.1 | 194.1269 | -11.038 | 2.167868 |  | -5.09164 | 3.55E-07 | 9.94E-05 | ATXN2 |
| rna-XM_038187661.1 | 96.01818 | -10.0225 | 1.554221 |  | -6.44856 | 1.13E-10 | 7.18E-08 | ZNRF3 |
| rna-XM_038187732.1 | 107.9526 | -3.37702 | 1.41876 |  | -2.38026 | 0.0173 | 0.401481 | LOC113845379 |
| rna-XM_038187735.1 | 240.3939 | -1.92232 | 0.658195 |  | -2.9206 | 0.003494 | 0.156442 | LOC119718652 |
| rna-XM_038187754.1 | 23.93334 | 5.218128 | 1.86857 |  | 2.792578 | 0.005229 | 0.200427 | LOC110354662 |
| rna-XM_038187787.1 | 16.04415 | -7.44099 | 2.218929 |  | -3.35342 | 0.000798 | 0.057053 | LOC119713003 |
| rna-XM_038187795.1 | 11.78184 | 3.774488 | 1.791875 |  | 2.106446 | 0.035166 | 0.563816 | LOC110351189 |
| rna-XM_038187799.1 | 40.40015 | 2.037243 | 0.716256 |  | 2.844292 | 0.004451 | 0.180559 | LOC101799700 |
| rna-XM_038187883.1 | 37.40714 | 2.745902 | 0.934726 |  | 2.937655 | 0.003307 | 0.150786 | LOC113845401 |
| rna-XM_038187901.1 | 18.1676 | -6.644 | 3.026057 |  | -2.1956 | 0.028121 | 0.516561 | LOC119718672 |
| rna-XM_038187912.1 | 20.64334 | 4.323221 | 1.886331 |  | 2.291868 | 0.021913 | 0.455817 | LOC119718673 |
| rna-XM_038187913.1 | 210.0975 | 1.702992 | 0.829961 |  | 2.051894 | 0.04018 | 0.596426 | PRKCQ |
| rna-XM_038187936.1 | 28.15578 | -8.25253 | 3.229039 |  | -2.55572 | 0.010597 | 0.309729 | LOC119718679 |
| rna-XM_038187972.1 | 960.65 | -2.41845 | 0.735543 |  | -3.28798 | 0.001009 | 0.067498 | LOC119713021 |
| rna-XM_038187976.1 | 1749.066 | -1.4348 | 0.590301 |  | -2.43063 | 0.015073 | 0.371635 | LOC113845378 |
| rna-XM_038188040.1 | 10.72661 | 6.903163 | 2.194158 |  | 3.146156 | 0.001654 | 0.096096 | EXD3 |
| rna-XM_038188077.1 | 298.1826 | 1.039084 | 0.388894 |  | 2.671896 | 0.007542 | 0.253157 | ABCA2 |
| rna-XM_038188086.1 | 170.9084 | 3.597326 | 1.070997 |  | 3.358857 | 0.000783 | 0.056048 | TSC1 |
| rna-XM_038188124.1 | 29.42664 | 8.3593 | 3.20546 |  | 2.607832 | 0.009112 | 0.282466 | RAPGEF1 |
| rna-XM_038188127.1 | 701.3386 | -1.42814 | 0.670443 |  | -2.13015 | 0.03316 | 0.551764 | RAPGEF1 |
|  |  |  |  |  |  |  |  |  |
