# Supplementary material for: In-depth transcriptome profiling of Cherry Valley duck lungs exposed to chronic heat stress
Source: Front Vet Sci. 2024 Jul 22;11:1417244. doi: 10.3389/fvets.2024.1417244 (PMC11298465; doi:10.3389/fvets.2024.1417244)

Figure S2 | Growth Performance Metrics at 43 Days Reflecting Cumulative Effects of CHS. (a) Body weight; (b) Leg muscle weight; (c) Liver weight; (d) Spleen weight; (e) Abdominal fat weight; (f) Breast muscle weight. Asterisks indicate levels of statistical significance (*p-value < 0.05, **p-value < 0.01).

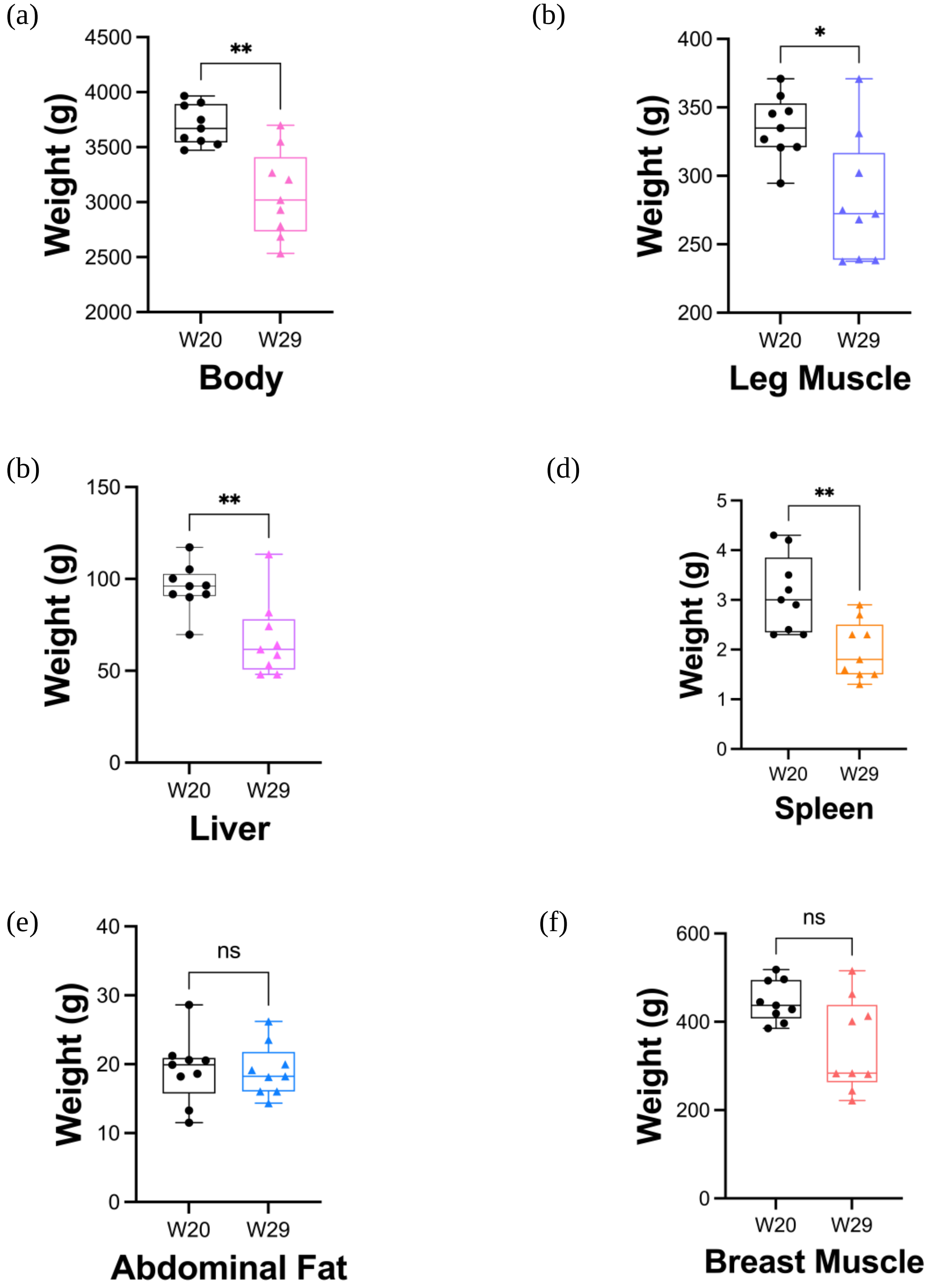

Supplement: Supplementary file 3 [file Data_Sheet_1.pdf]
