# Supplementary material for: In-depth transcriptome profiling of Cherry Valley duck lungs exposed to chronic heat stress
Source: Front Vet Sci. 2024 Jul 22;11:1417244. doi: 10.3389/fvets.2024.1417244 (PMC11298465; doi:10.3389/fvets.2024.1417244)

Figure S1 | Cherry Valley Ducks in Controlled Environmental Chambers. (a) Ducks under sustained temperature regulation; (b) Ducks subjected to fluctuating ambient temperatures.

(a)

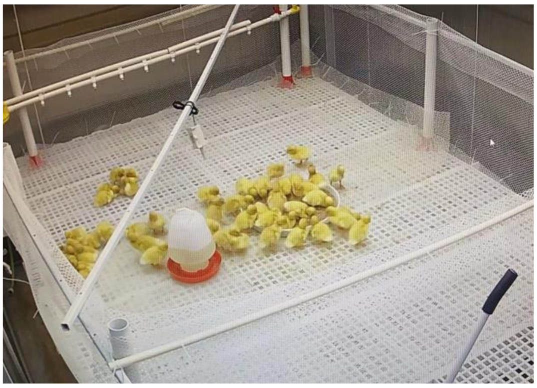

(b)

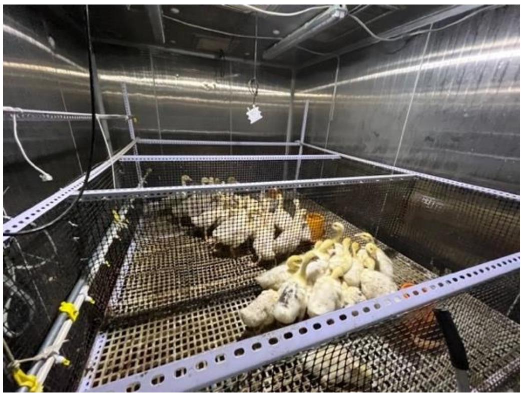

Supplement: Supplementary file 4 [file Image_1.pdf]
